# Genome-Wide Locations of Potential Epimutations Associated with Environmentally Induced Epigenetic Transgenerational Inheritance of Disease Using a Sequential Machine Learning Prediction Approach

**DOI:** 10.1371/journal.pone.0142274

**Published:** 2015-11-16

**Authors:** M. Muksitul Haque, Lawrence B. Holder, Michael K. Skinner

**Affiliations:** 1 Center for Reproductive Biology, School of Biological Sciences, Washington State University, Pullman, Washington, 99164–4236, United States of America; 2 School of Electrical Engineering and Computer Science, Washington State University, Pullman, Washington, 99164, United States of America; Massachusetts General Hospital, UNITED STATES

## Abstract

Environmentally induced epigenetic transgenerational inheritance of disease and phenotypic variation involves germline transmitted epimutations. The primary epimutations identified involve altered differential DNA methylation regions (DMRs). Different environmental toxicants have been shown to promote exposure (i.e., toxicant) specific signatures of germline epimutations. Analysis of genomic features associated with these epimutations identified low-density CpG regions (<3 CpG / 100bp) termed CpG deserts and a number of unique DNA sequence motifs. The rat genome was annotated for these and additional relevant features. The objective of the current study was to use a machine learning computational approach to predict all potential epimutations in the genome. A number of previously identified sperm epimutations were used as training sets. A novel machine learning approach using a sequential combination of Active Learning and Imbalance Class Learner analysis was developed. The transgenerational sperm epimutation analysis identified approximately 50K individual sites with a 1 kb mean size and 3,233 regions that had a minimum of three adjacent sites with a mean size of 3.5 kb. A select number of the most relevant genomic features were identified with the low density CpG deserts being a critical genomic feature of the features selected. A similar independent analysis with transgenerational somatic cell epimutation training sets identified a smaller number of 1,503 regions of genome-wide predicted sites and differences in genomic feature contributions. The predicted genome-wide germline (sperm) epimutations were found to be distinct from the predicted somatic cell epimutations. Validation of the genome-wide germline predicted sites used two recently identified transgenerational sperm epimutation signature sets from the pesticides dichlorodiphenyltrichloroethane (DDT) and methoxychlor (MXC) exposure lineage F3 generation. Analysis of this positive validation data set showed a 100% prediction accuracy for all the DDT-MXC sperm epimutations. Observations further elucidate the genomic features associated with transgenerational germline epimutations and identify a genome-wide set of potential epimutations that can be used to facilitate identification of epigenetic diagnostics for ancestral environmental exposures and disease susceptibility.

## Introduction

Epigenetics is defined as “molecular factors and processes around DNA that regulate genome activity independent of DNA sequence and are mitotically stable” [1]. In 1942 Conrad Waddington was the first to coin the term epigenetics related to how environment influences development in conjunction with genotype and phenotype [2]. The molecular factors currently known to be epigenetic processes include DNA methylation, histone modifications, chromatin structure and selected non-coding RNA [1, 3–7]. Epigenetics has been shown to be a critical factor in normal biology, disease etiology and evolution [1, 8]. A combination of epigenetic and genetic molecular mechanisms will be essential for nearly all biological processes. However, genetics has been the primary molecular component considered for nearly all aspects of biology. For example, DNA sequence and genetics has been considered the primary form of inheritance. More recently, environmentally induced epigenetic transgenerational inheritance has been described in species from plants to humans [1]. This provides an additional epigenetic mechanism for inheritance to consider [9] and helps explain forms of familial inheritance not easily explained with classic genetics.

Epigenetic transgenerational inheritance is defined as “germline transmission of epigenetic information between generations in the absence of direct environmental exposure” [1]. A growing number of environmental factors have been shown to promote the epigenetic transgenerational inheritance of disease and phenotypic variation from nutrition, stress or toxicants [1, 10]. The environmental chemicals shown to promote transgenerational inheritance of disease and sperm epimutations include the agricultural fungicide vinclozolin [11], pesticide permethrin and insect repellent N,N-diethyl-meta-toluamide (DEET) [12], pesticides methoxychlor [13] and dichlorodiphenyltrichloroethane (DDT) [14], plastic derived compounds bisphenol A (BPA) and phthalates [15], and hydrocarbon mixtures (jet fuel, JP8) [16]. The F0 generation gestating female rats were transiently exposed during fetal gonadal development and then the F1, F2 and F3 generations generated [1, 11]. The transgenerational F3 generation (i.e., no direct exposure) was found to have a large number of high frequency disease states including testis, ovary, prostate, mammary and kidney disease [17]. Analysis of the F3 generation male sperm demonstrated differential DNA methylation regions (DMRs) that were highly reproducible and exposure specific [18, 19]. These DMRs were termed epimutations and ranged in number for genome-wide promoter regions from 30 to 300 depending on the specific exposure [13, 14, 18]. Each transgenerational set of epimutations was found to be exposure specific with negligible overlap between exposures [1, 18]. In addition to the transgenerational sperm epimutations, somatic cell transgenerational epimutations for the agricultural vinclozolin lineage F3 generation testicular Sertoli cells and ovarian granulosa cells were utilized in a similar analysis [20, 21]. As found with the exposure specific sperm epimutations, the somatic cell epimutation sets were cell specific with negligible overlap. These somatic cell transgenerational epimutation data sets were also used independently in the current study as training sets for machine learning predictions for somatic cells versus germ cells.

These transgenerational epimutations were used to identify common genomic features associated with the epimutations. The first genomic feature found associated with all epimutations [18] was a low CpG density of less than 10 CpG per 100 bp which were characterized as “CpG deserts” containing small CpG clusters with differential DNA methylation [22]. The second set of genomic features identified were unique DNA sequences generally within a few hundred base pair of the differential DNA methylation region [23]. These DNA sequence motifs were previously shown to associate with binding proteins that bend DNA [19, 23]. In addition to these genomic features, a number of other genomic features previously shown to associate with epigenetic sites were also selected for the analysis [24].

The current study was designed to use a machine learning analysis to further study the genomic features of the transgenerational germline epimutations and predict genome-wide sites that may be susceptible to become environmentally modified epimutations. A previous study used known imprinted genes and associated genomic features in both mouse and humans to predict additional imprinted genes [25, 26]. This study identified critical genomic features and demonstrated approximately 600 new potential imprinted genes [25]. Although this previous analysis investigated a distinct epigenetic process (i.e., imprinting), a similar rationale was used in the current study. The approach used known transgenerational sperm epimutation data sets from a variety of exposures as a training set for a machine learning analysis. A similar approach was used with transgenerational somatic cell epimutation data sets to determine differences and similarities between the germline and somatic cell epimutations. The genomic features previously identified and additional features were used to identify genome-wide regions susceptible to become transgenerational epimutations.

A machine learning analysis uses a known training set(s) of data to construct a classifier based on known features to classify larger unknown data sets [27]. Generally an issue with machine learning analysis is that a relatively small set of positive traits are used in reference to a much larger set (i.e., volume) of data with negative (non-relevant) traits. This introduces significant bias in the results due to the imbalance between data sets [28]. In addition, often large sets of predicted features are used in machine learning analysis such that only a small number of critical features are relevant [29]. This can also reduce the efficiency and bias the machine learning analysis. Two different machine learning techniques can be used to address these issues. Active learning (ACL) is the selection of important features and examples for an oracle to classify. The addition of generalized query to the ACL allows selection of the optimal features in these examples which the oracle can classify. These two techniques facilitate the training for the machine learning classifier [30]. ACL can also be used to select the most important features and provide insights into the critical features identified. Imbalance class learners (ICL) can be used to reduce the data set imbalance bias and allow for a more accurate analysis [31]. The current study developed a novel two-step (sequential) machine learning analysis involving a combination of an initial active learning step followed by an imbalance class learner (ACL-ICL) protocol. This newer technique provides a more tightly integrated approach for a more efficient and accurate machine learning analysis.

The objective of the current study is to utilize the novel machine learning approach with known transgenerational sperm epimutations and associated genomic features to predict genome-wide regions that have a susceptibility to develop into transgenerational epimutations. Observations provide insights into the genomic features associated with epimutations and help understand why these sites may be transgenerationally programmed. Previous studies [1, 18] have suggested exposure specificity in epimutations, as well as disease susceptibility later in life. Therefore, genome-wide transgenerational epimutation data sets for germ cells and somatic cells will be invaluable in future identification of diagnostics for environmental exposures and later life disease susceptibility.

## Results

**Fig 1:**
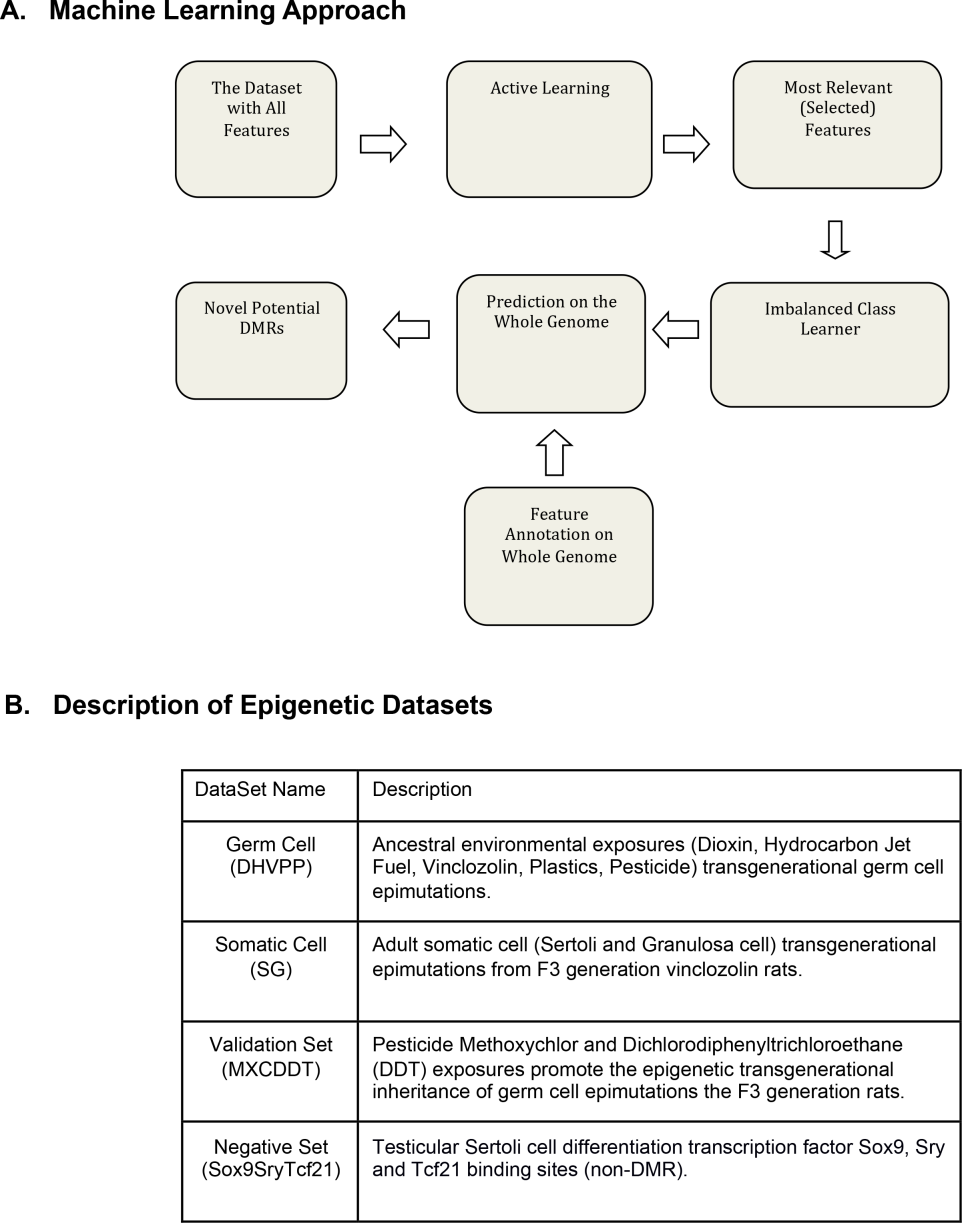
Machine learning approach and training set description. (A) Two-step machine learning framework for DMR identification. (B) Description of datasets: germ cell DHVPP; somatic cell (SG); MXC-DDT; and non-DMR Sox9SryTcf21.

**Fig 2:**
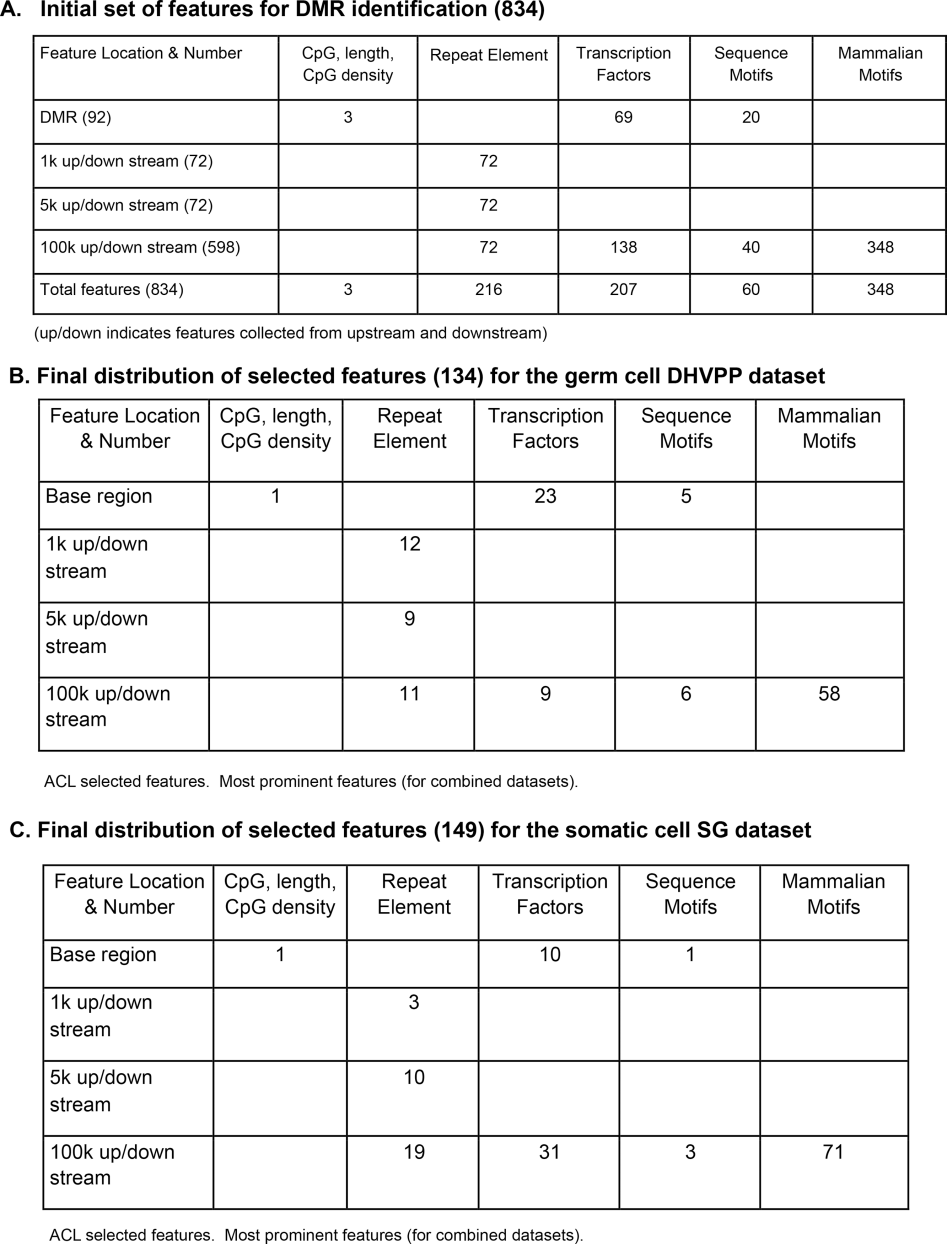
DMR genomic features. (A) Entire list of features. Number Initial set of extracted features for DMR identification (up/down indicates features collected from upstream and downstream). (B) Reduced selected features for germ cell combined datasets. DHVPP (Dioxin, Hydrocarbon Jet Fuel, Vinclozolin, Plastics, Pesticide) final feature list (134). Up denotes upstream, Dn denotes downstream, features without Up and Dn initial have been extracted from the DMR base region itself. (C) Reduced selected features for somatic cell combined datasets. SG (Sertoli-Granulosa) final feature list (149). Up denotes upstream, Dn denotes downstream, features without Up and Dn initial have been extracted from the DMR base region itself.

The machine learning approach used in this study (Fig 1A) uses the generalized query based ACL method to find the most important samples and features for the epigenetic datasets. Initially the number of features collected for the epigenetic dataset was 834 for each of the two transgenerational datasets. The germ cell (sperm) dataset was Dioxin-Hydrocarbons (Jet Fuel)-Vinclozolin-Plastics-Pesticides (DHVPP), and somatic cell dataset was Sertoli-Granulosa (SG). Fig 1B contains descriptions of the different epimutation datasets. The selected 834 genomic features can be grouped into four sub-groups (Fig 2A). They are CpG density and related information (3 total features), repeat elements (216 total features), transcription factors (207 total features) and DNA sequence motifs (60 total features). The sequence motif group has a subgroup called mammalian motifs (348 total features) as these features were collected from the online JASPER dataset [32]. All these features were annotated for the epimutation regions (the identified DMR regions), as well as for sequences 1k, 5k, and 100k upstream and downstream of the DMRs. ACL was run on the DHVPP and SG datasets separately and only those features that appeared greater than 5 times, as well as some manually selected important features were chosen as the most relevant features for further analysis (Fig 2B & 2C). This information for each of these datasets was combined and ACL trained on these feature sets. Once ACL training was complete ICL training was used for prediction across the whole genome for each germ cell and somatic cell data set separately (Fig 1A).

Since most of the DMR locations are found within 600bp to 1500bp windows, a non-overlapping sliding window of 1000bp was used on each chromosome to identify potential DMR candidate sites. The original 834 selected genomic features were extracted/annotated for the entire rat genome DNA sequence. The number of initial extracted/annotated feature sets is shown in Fig 2A. For each of the 21 rat chromosomes (autosomes and X chromosome) a sliding non-overlapping window size of 1000 bases was used to create a total of 2,630,424 sites. In the same manner as the training dataset, FASTA files were created. RepeatMasker was run and finally a list of 834 features was extracted from each of these sites. This is the test set used for prediction. Once the training was complete a prediction on the whole genome was made. The proposed approach to find potential new DMRs is the first to construct a robust classifier (using both imbalanced class and active learning approach) which minimizes false positives, and then scan the genome for locations which are highly likely to be DMRs, Fig 1A.

Once these features were identified, annotated and extracted from the training datasets, active learning was used to find the most relevant features. The features which appeared 5 or less times were considered don't care attributes (irrelevant features) and a set of manually selected features was taken as the list of most relevant features. The most relevant features for the two training datasets are presented in Fig 2B & 2C and S1 Table. The list of features include the following categories: (a) CpG information (b) repeat elements (c) transcription factors (d) sequence motifs and (e) mammalian motifs. The CpG Information contains three features: length of the sites in base pair, number of CpG sites, and CpG density (number of CpG sites per 100 bases). The transgenerational epimutations have been found in low CpG density regions (termed CpG deserts) [22]. The genomic feature of low CpG density was found to be one of the most important features for both the somatic and germ cell prediction datasets. The repeat elements original list contained a total of 216 repeat features. Both the somatic and sperm datasets had 32 repeat elements (with significant overlaps) in their final list of somatic 134 and sperm 149 features (Fig 2). The original transcription factor group contained 207 features. In the final list for sperm (DHVPP) there were 32 transcription factor features and for the somatic cells (SG) there were 41 features. The DNA sequence motifs [33, 34] had 60 original features selected for this study. For the sperm (DHVPP) dataset there are 11 sequence motif features and for the somatic (SG) dataset there are 4 sequence motifs critical features. Mammalian motifs originally considered involved 348 features from the JASPER dataset [32]. For the sperm (DHVPP) there were 58 mammalian motif features while for the somatic cell (SG) there were 71 of them (Fig 2B & 2C).

Once the final list of features was selected for the two datasets they were used for training in the ICL, and used for the genome wide prediction. The sperm and somatic cell analysis was done separately with the relevant list for each. The initial number of predicted epimutation sites identified was 48,557 sites for the sperm (DHVPP) and 28,564 sites for the somatic cells (SG). However, after an initial number of individual sites were found, only those with three or more consecutive sites were merged to create the most stringent list of potential susceptible DMR sites. The reason for focusing on three or more consecutive sites is that single predicted sites have a lower statistical significance and a higher potential for false positives. Although the single sites are viable potential DMR to consider, a more stringent analysis of DMR was used of three or more consecutive probes being present to further investigate the potential differential DNA methylation regions. These three or more consecutive sites were merged to create the list of potential susceptible DMR sites. The final list of potential DMR for the sperm DHVPP analysis was 3,233 sites and for the somatic cell SG analysis was 1,503 sites. The entire list of these potential DMR sites are presented in A & B of S2 Table.

**Fig 3:**
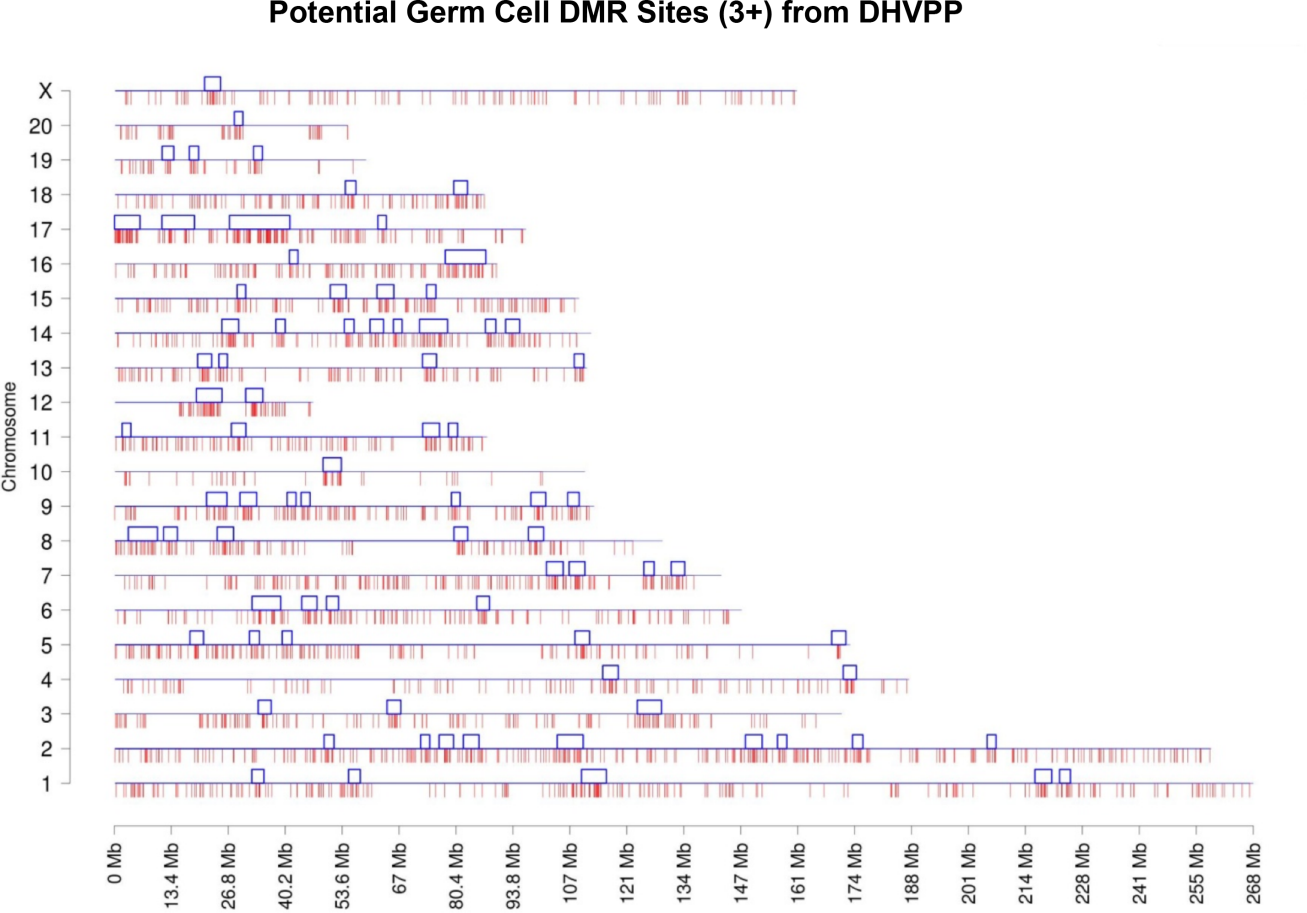
Chromosomal plot of germ cell dataset DHVPP shows the predicted 3+ sites and the clusters. Predicted potential DMR sites (3,233) when DHVPP is used as the training set with red lines in the bottom and clusters (80) with blue boxes on the top for each chromosome line. X-axis shows each of the 21 chromosomes while Y-axis shows the length of the chromosome with predicted potential DMR locations. The clusters are regions which indicate over-representations of the sites within the small sub-section of the genome.

**Fig 4:**
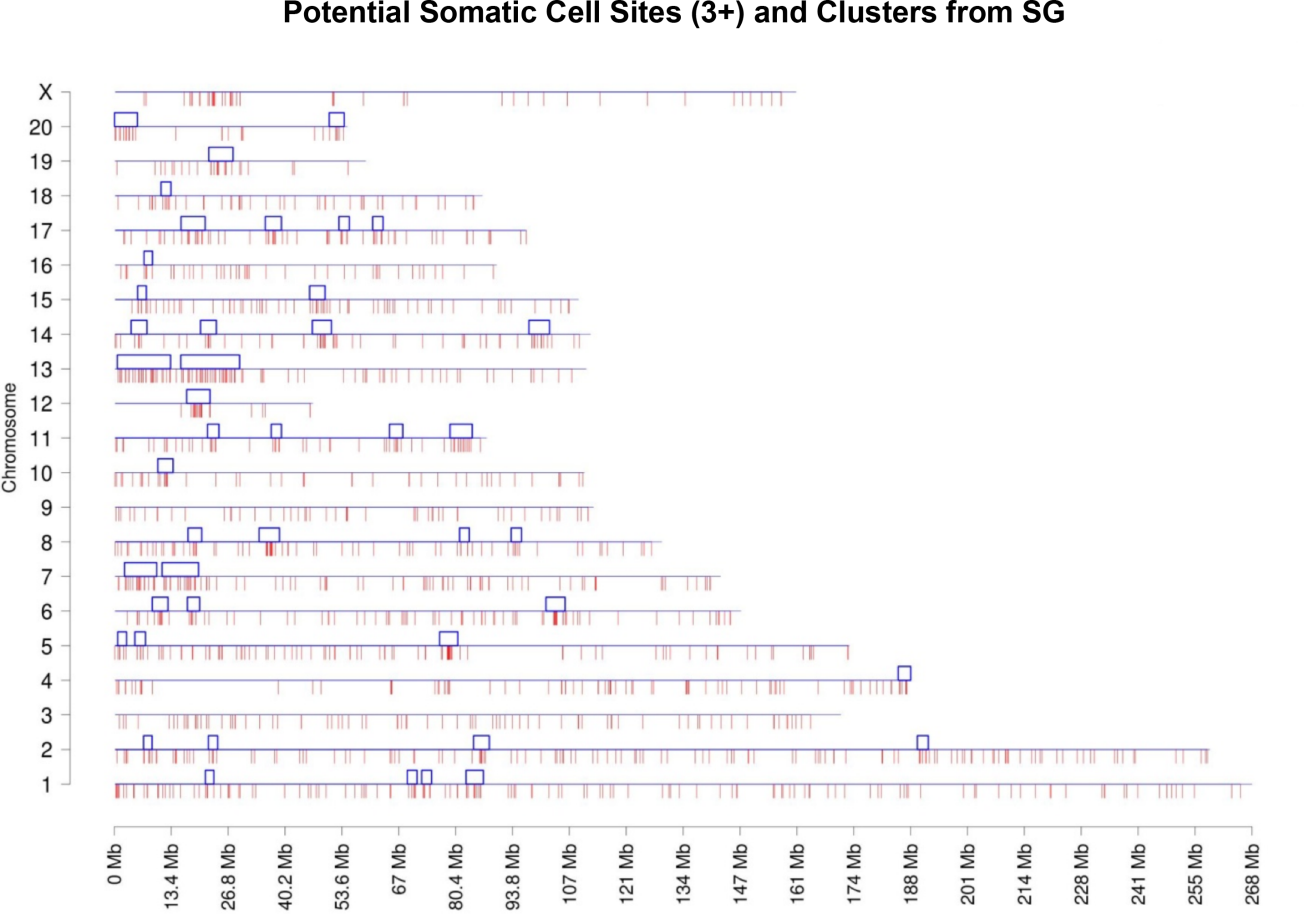
Chromosomal plot of somatic cell dataset SG shows the predicted 3+ sites and the clusters. Potential predicted DMR sites (1,503) when SG is used as the training set to predict on the rest of the genome. X-axis shows each of the 21 chromosomes while Y-axis shows the length of the chromosome with predicted potential DMR locations. Red lines in the bottom are shown as potential DMR sites and clusters (44) with blue boxes are shown on the top of each chromosomes.

The chromosome plots for the datasets DHVPP (Fig 3) and SG (Fig 4) are presented and the predicted DMR/epimutation regions are shown on all chromosomes. Once the three or more consecutive sites were identified a cluster analysis was performed to identify DMR co-localization. The methods section describes the cluster construction procedure for the identification of statistically significant over-represented within the regions DMR. A total of 80 clusters were formed from the predicted 3,233 DMR sites for the germline (DHVPP) dataset as shown in Fig 3. The average size of the germline DMR clusters were 3,574,375 bases and 32% of the total sites fall within those clusters. For the somatic cell (SG) dataset a total of 44 DMR clusters were identified from the predicted 1508 DMR sites. Average cluster sizes are 4,046,591 bases long and 27% of the total sites fall within these clusters, Fig 4. The list of predicted cluster regions is presented in S3 Table and shown in Figs 3 & 4. These DMR clusters demonstrate that the potential DMRs are in part localized in certain regions of the genome. These clusters of potential DMRs are speculated to act as Epigenetic Control Regions (ECR) to regulate gene expression within the clusters [35].

**Fig 5:**
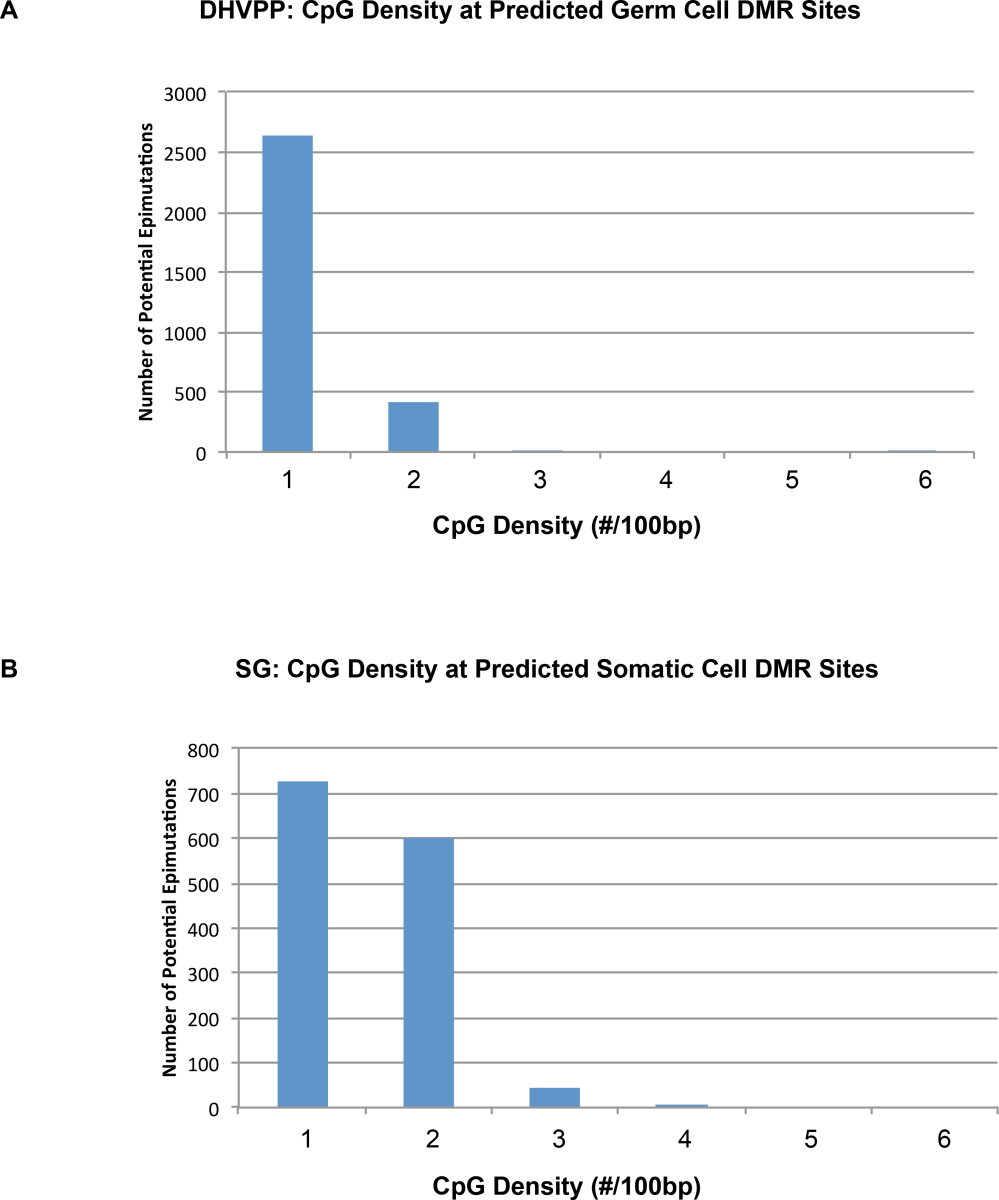
CpG density plot showing number of predicted DMR sites correlated with CpG density. (A) CpG density from the potential predicted germ cell DMR sites (3,234) when DHVPP is used as the training set to predict genome-wide. (B) CpG density from the potential predicted somatic cell DMR sites (1,502) when SG is used as the training set to predict genome-wide. X-axis shows the number of CpG's per 100bases on average while Y-axis shows the number of sites.

**Fig 6:**
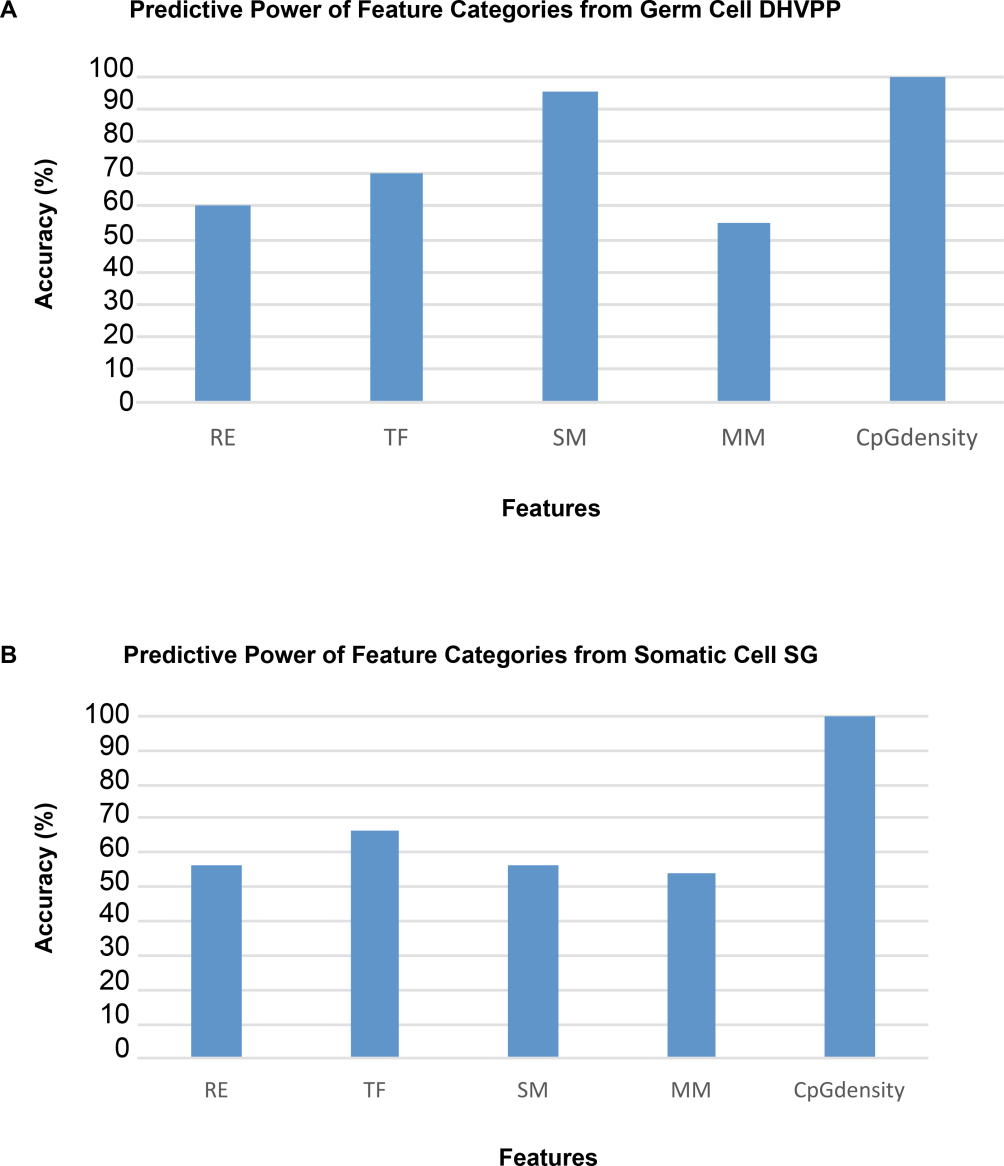
Predictive power of specific features. (A) Groups of features with their predictive power (percent accuracy) for the DHVPP dataset. (B) Groups of features with the predictive power (percent accuracy) for the SG dataset. The features include RE—Repeat Elements, TF- Transcription Factors, SM- Sequence Motifs, MM-Mammalian Motifs with their predictive power indicated.

The following analyses investigated the genomic features of the predicted DMR/epimutations. The initial analysis was to check the CpG density of the regions which were identified as potential DMRs. The predicted DMR CpG density (number of CpG in each 100 bases) distribution was determined and shown in Fig 5. Interestingly, all the predicted DMR sites had densities of <2 CpG/100bp. This observation supports the fact that most DMRs are found in low CpG density regions (termed CpG deserts) [22] instead of regions of high CpG density (called CpG islands or shores) [36]. Prediction power refers to the number of DMR that contain a specific feature. The percentage of predicted DMR that had the CpG density feature (i.e., prediction power) was 100% for both the germ cell and somatic cell predicted DMR data sets, Fig 6.

Transcription factor binding sequence motifs and mammalian sequence motifs were the next features investigated. These features were collected from the DMR region and upstream and downstream of the DMR. Features were extracted from 1k, 5k and 100k upstream and downstream regions of the DMR region. The consensus sequence correlations to the prediction of DMRs are shown in Fig 6. For the predicted DMR in DHVPP that had the sequence motif features, the prediction power was high (above 90%) while for SG the prediction power of transcription factor features was above 60%. This was compared to the 100% predictive ability of CpG density.

**Fig 7:**
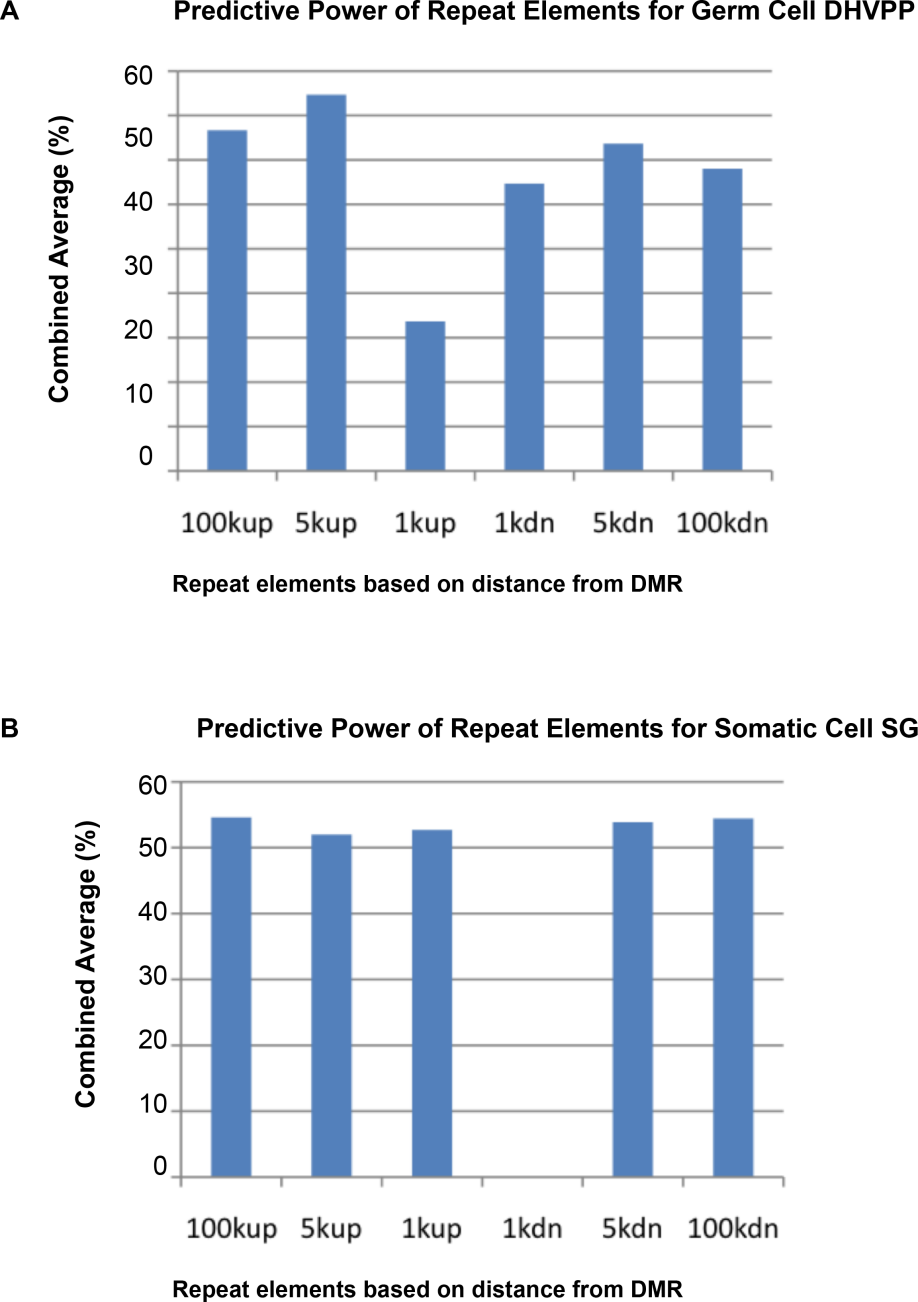
Predictive power of repeat elements accuracy based on genomic location of 1k, 5k, 100k from the DMR. (A) Combined average when each group of repeat elements are used for prediction for DHVPP dataset. (B) Combined average when each group of repeat elements are used for prediction for SG dataset. Shows combined repeat elements in the 100k, 5k and 1k upstream and downstream regions.

The repeat elements were chosen as a group of features (based on their location and distance from the DMR region) and for the predicted DMR that had the feature, prediction power was calculated to see which repeat elements gave the highest accuracy. All the repeat elements were grouped into 1k, 5k, 100k upstream and downstream. The predictive power of repeat elements for DHVPP and SG is shown in Fig 7. The repeat elements in the 100k upstream region had a slightly higher predictive power for the SG dataset. The repeat elements in the 5k upstream had higher predictive power among the germ cell groups. The average DMR sites for DHVPP had a 3564 base length and for SG had a 4213 base length. The details are given in Fig 2B & 2C.

**Fig 8:**
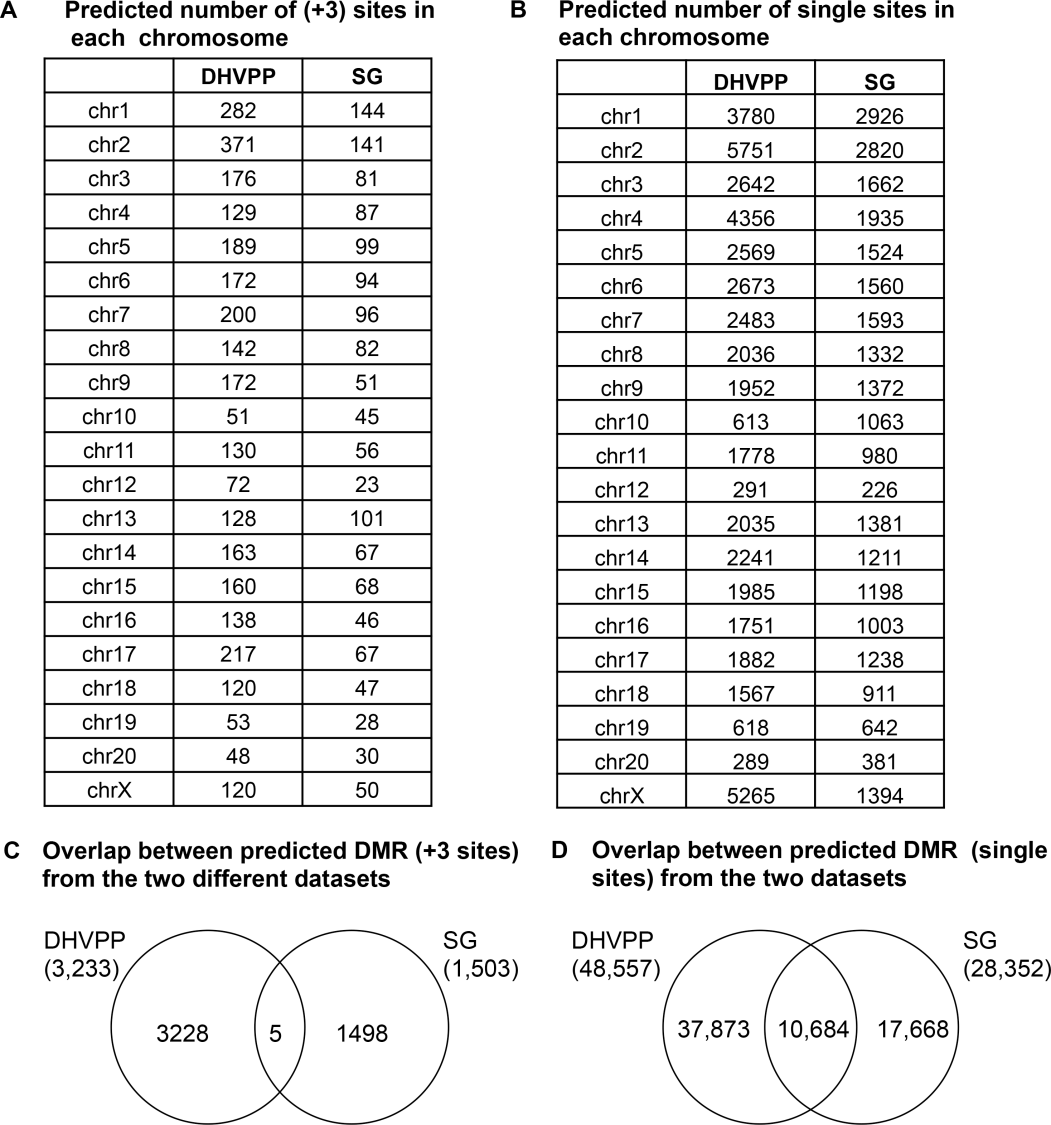
Genomic chromosome locations of predicted DMR and overlap between germ cell and somatic cell predicted sites. (A) Germ cell DHVPP and somatic cell SG predicted number of (+3) sites in each chromosome. (B) Germ cell DHVPP and somatic cell SG predicted number of single sites in each chromosome. (C) Overlap between predicted DMR (sites) from the two different datasets. (D) Overlap between predicted DMR (sites) from the two different datasets.

A comparison was made between the genome-wide predicted DMR/epimutation in the germ cell data sets and somatic cell data sets. The distribution of the predicted DMR on the various chromosomes is shown in Fig 8A & 8B. Overlap between the potential predicted DMR sets derived from the germline DHVPP and somatic SG datasets showed only five common predicted sites, Fig 8C. In addition, the overlap with the single predicted DMR sites identified 10K sites with overlap, Fig 8D. This shows that the germline (sperm) predicted DMR and somatic (SG) cells predicted DMR are generally distinct. The sperm and somatic cell predicted DMR were obtained with different feature sets and independently. Therefore, the learned classifiers from the germline (DHVPP) and the somatic cell (SC) datasets are also distinct. This corresponds to the differences in contributions in the various genomic features. Since the original DMR somatic SG and germline DHVPP DMR sites had no overlap between them in the training data, it was not surprising very little overlap was observed among the predicted DMRs. These overlapped sites are identified in S2 Table and shown in Venn diagrams in Fig 8C & 8D.

**Fig 9:**
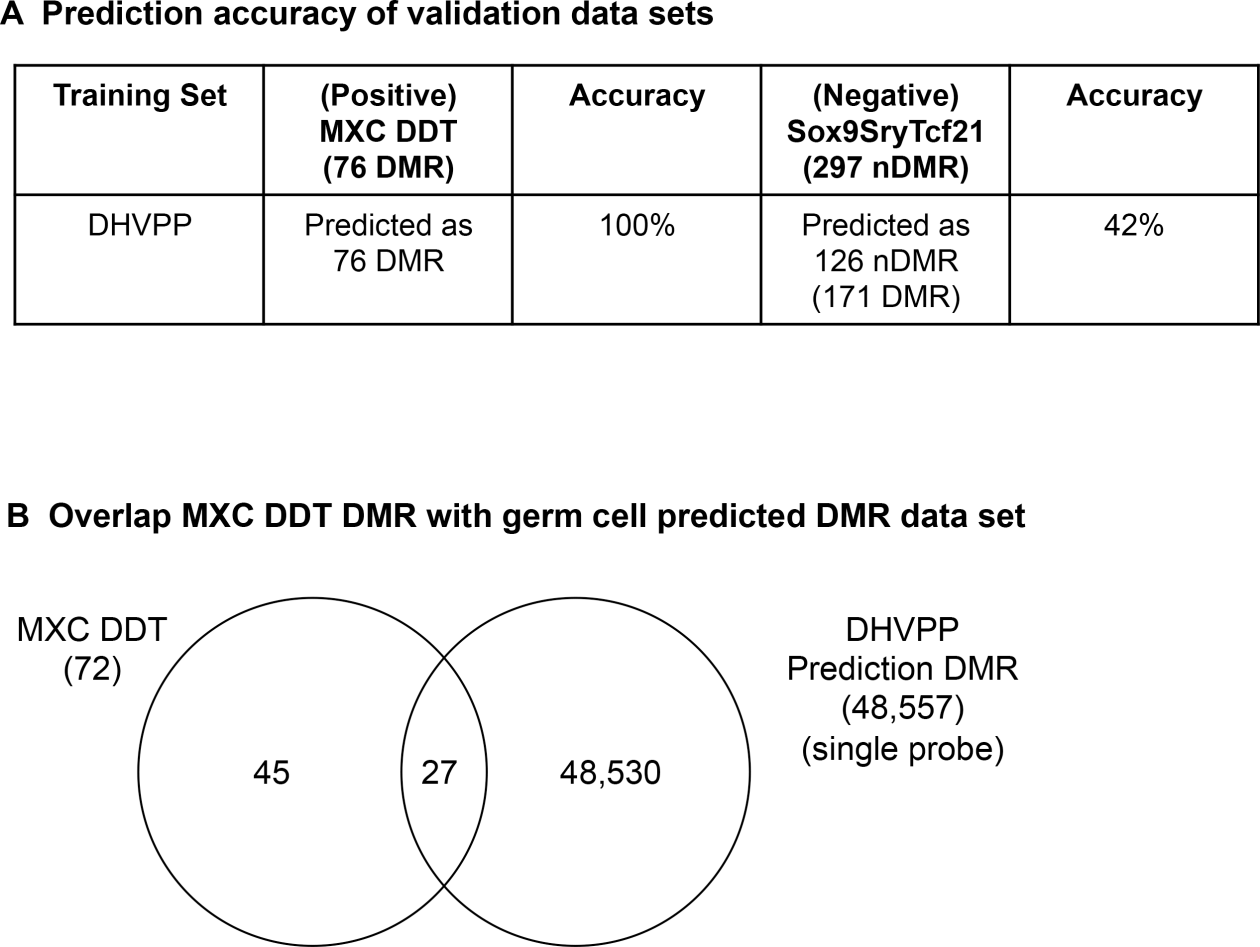
Validation of the germ cell DMR data set. MXC-DDT used as positive testing set and Sox9SryTcf21 as non-DMR negative testing set. (A) Prediction of the training set DHVPP with the positive MXC-DDT and negative Sx9SryTcf21 validation data set. (B) Overlap of germ cell validation set MXC-DDT with predicted DHVPP single probe data set.

In order to help validate the machine learning results for the predicted germ cell DMR data set a positive validation analysis was performed. For the positive validation analysis the predicted DMR datasets were compared to two more recently developed sperm DMR datasets which were not used as test sets in the machine learning analysis. The first was a DDT transgenerational sperm DMR set [14] and second a methoxychlor (MXC) data set [13]. The two DMR positive control data sets were combined and termed the sperm MXC-DDT DMR data set. The description of the datasets is given in Fig 1B. The germ cell learned classifier accurately predicted all the DMRs in the sperm MXC-DDT dataset, 100% prediction accuracy, Fig 9A. Prediction accuracy is defined as the number of previously identified DMR that were identified by the computational tool. In addition, a comparison of the MXC-DDT DMR with the predicted genome-wide sperm DMR showed 38% overlap with the single site comparison, Fig 9B. Therefore, this positive validation sperm transgenerational DMR dataset was accurately predicted and had partial overlap, helping to validate the approach and predicted germ cell DMR dataset. Alternately, a negative validation analysis used a negative non-DMR (nDMR) data set involving transcription factor binding sites for SOX9, SRY and TCF21 [37, 38] termed Sox9SryTcf21 with a total of 297 nDMR. This negative dataset was obtained with similar technology as the DMR sets. This involved a chromatin immunoprecipitation (ChIP) followed by a promoter tiling array (ChIP-Chip) analysis for this nDMR set versus the methylated DNA immunoprecipitation (MeDIP) followed by the tiling array (MeDIP-Chip). Using the negative nDMR data set and the machine learning algorithm only a 47% prediction accuracy (SG) and 42% prediction accuracy (DHVPP) was obtained while predicting all nDMR in Sox9SryTcf21 dataset, Fig 9A. A prediction accuracy of 50% or less is neutral with no prediction potential. Therefore, the negative validation with the nDMR demonstrated negligible overlap with the predicted DMR dataset and poor accuracy in the machine learning analysis.

## Discussion

Previous studies have demonstrated a variety of environmental factors from abnormal nutrition [39–45] to toxicant exposures can promote the epigenetic transgenerational inheritance of disease susceptibility and germline (e.g., sperm) epimutations [1]. Examples include the agricultural fungicide vinclozolin [11, 17], the industrial contaminant dioxin [46, 47], a hydrocarbon mixture jet fuel (JP8) [16], the plastic derived compounds bisphenol A (BPA) and phthalates [15, 48, 49], the pesticides methoxychlor [11, 13] and dichlorodiphenyltrichloroethane (DDT) [14], and permethrin and N,N-Diethyl-meta-toluamide (DEET) [12]. All these environmental exposures of a gestating female (F0 generation) during the period of fetal gonadal sex determination promoted the epigenetic transgenerational (i.e., F3 generation) inheritance of disease. The transgenerational disease observed varied between the exposures, but generally involved abnormalities in the testis (spermatogenic cell apoptosis), ovary (polycystic ovarian disease), kidney (cyst development), prostate (epithelial cell atrophy), and behavioral abnormalities including mate preference changes and anxiety [1]. Interestingly, the chromosomal locations of the transgenerational sperm epimutations were generally distinct between the different exposure lineages [18]. Therefore, the sperm were found to have an exposure specific set of epimutations [1] and the epimutations all had common genomic features of a low CpG (<10 CpG / 100 bp) density (i.e., CpG deserts) [22] and unique DNA sequence motifs [23].

The current study was designed to use these various transgenerational epimutation datasets as training sets in a novel sequential machine learning approach to identify the potential genome-wide locations of transgenerational epimutations. Although previous machine learning approaches applied active learning or imbalance class learning independently, the sequential use for a biological data set is novel. The training datasets from the epigenetic transgenerational (F3 generation) inheritance of sperm epimutations from various exposure lineages included; dioxin [46], jet fuel [16], vinclozolin [16, 18, 19, 46], plastics (BPA phthalates) [15] and pesticide (permethrin and DEET) [12, 15]. These exposure specific sperm epimutation datasets were used to develop the machine learning algorithm to predict the genome-wide locations of sperm epimutations. In addition, transgenerational somatic cell epimutation datasets were used to predict genome-wide locations of potential somatic epimutations. The testicular Sertoli cell and ovarian granulosa cells were purified from adult vinclozolin lineage F3 generation tissues and these cell specific epimutations identified [20, 21]. These transgenerational somatic cells epimutation datasets were then used independently as training sets in the machine learning approach to develop the algorithm for transgenerational somatic cell epimutations and compare to that of transgenerational germline epimutation predictions.

In a previous research study that looked into finding potential imprinted genes in human and mouse genomes, Jirtle and colleagues mined the mouse genome and found thousands of relevant features for machine learning prediction of potential imprinted genes [25]. Imprinted genes are parent of origin monoallelic expressed genes with critical developmental functions [50]. Mining the DNA sequence characteristics up to 100kb upstream and downstream around known imprinted genes developed genomic features and training sets to develop a prediction algorithm [25]. They used the Equbits Foresight (http://www.equbits.com) classifier and predicted 722 new potential imprinted gene sites. Their study examined 23,788 annotated autosomal mouse genes and identified 600 potential mouse imprinted genes [25]. The same group later mined the human genome for new imprinted sites [26]. They again used the Equbits Foresight which uses the Support Vector Machine (SVM) classifier and 622 features and used their own SMLR (sparse multinomial logistic regression) [51] classifier with 820 features to predict novel human imprinted genes [26]. A second study by another group looked into the correlation of different genomic features in DNA methylation of CpG islands [52]. They mined features from 190 CpG islands from human chromosome 21 and tested it on the rest of the CpG islands in the genome for finding potential methylated CpG islands. A correlation among different features identified potential different methylation profiles for different tissue types and for different diseases [52]. The main difference of the proposed approach with the imprinted gene research is that active learning is used to identify a sub-group of features for each queried training example instead of using a global feature reduction [25, 26]. For the second study the main difference is that their approach looks into DNA methylation in CpG islands while the current study looks into genome wide methylation patterns including low density CpG regions, unlike dense CpG regions in CpG islands [52].

The purpose and advantages of using active learning and imbalanced class learning in a combined approach over traditional machine learning classification needs to be clarified. Biological datasets come with a set of inherent problems. Most data that researchers are interested in (e.g., positive cases) are rare (i.e., imbalanced) in contrast to all other characteristics or features. Efficient learning can be performed only when target concepts from both the classes (e.g., differential DNA methylation regions (DMR) and non-DMR) are learned well to distinguish them separately while learning from only the relevant features. Such interesting computational problems can be approached using specific machine learning techniques. Computational goals of the proposed study were to identify the most relevant features and address the class imbalance problem. The genomic characteristics of the DMRs are used as features for the learners. Active learning intelligently chooses the best instances / features to learn from [53, 54]. The approach uses Generalized Query Based Active Learning (GQAL) which not only can choose the best features to learn from, but also select the most relevant features for this instance for learning. This is accomplished by constructing intelligent queries by removing irrelevant features from the query which an Oracle (e.g., a human expert) can answer easily. This approach allows the learner to label multiple instances at the same time instead of labeling one instance per query. In addition, instead of using a global feature reduction (where a set of features are removed in the beginning of the training) GQAL uses a subset of features at each iteration by using local feature selection. This makes use of the most potential power of the features and it maximizes the use of a subset of features for learning. The GQAL approach has been tested on 13 datasets besides epigenetics and compared with 3 other classifiers (KNN, SVM and NB) [30] and later with (AdaBoost, Decision Trees, RandomForest and Logistics) and the GQAL was found to be the most efficient for the epigenetic dataset. The current study combines these two approaches into a single sequential computational tool.

A number of approaches have been used to address the imbalanced class problem [55–57]. Instead of using an under-sampling or an oversampling technique [55, 56] to reduce or increase the size of each of the classes to make them balanced, the current approach uses a boosting technique called AdaBoost or “Adaptive Boosting” [58, 59]. Boosting is a popular method to increase weights of certain examples while decreasing the weights of other examples for efficient balanced learning. This approach allows the learning algorithm to learn target concepts well from both the classes. This addresses the imbalance class problem. For the AdaBoost algorithm a weak classifier called Tree Augmented Bayesian Network (TAN) [60] was chosen as the classifier. This is a restrictive Bayesian learner which performs better than the Naïve Bayes Classifier (NBC) [61]. The TAN boosted imbalances class learner has been tested on 5 datasets including 2 epigenetic datasets and compared with 2 other imbalanced class learners (Subset Sampling Optimization and EasySensemble) and 5 other regular classifiers (SVM, Logistics, Decision Trees, RandomForest and AdaBoost) [31] and the TAN AdaBoost was found to be the most efficient in the epigenetic dataset. Currently there are no available approaches applied to epigenetics data that look into either of these problems from the machine learning point of view.

Active learning using the GQAL approach on the transgenerational sperm DHVPP epimutation was done over a 10 fold cross validation. During training GQAL found 36% of the features to be redundant and used 245 samples averaged over all iterations. Once training was complete the learning algorithm was tested on an independent test set and an accuracy of 99.2% was achieved. In contrast, for the somatic cell (SC) dataset GQAL removed 14% of the features as redundant and used 290 samples averaged over all iterations. Again after completion of training the learned classifier was tested on an independent test set and achieved an accuracy of 97.7%. This shows the power of the GQAL approach [30]. While Active Learning removes redundant features, boosting performed balanced learning on the epigenetic datasets.

Additional analysis was done to determine the predictive power of specific groups and individual genomic features. The percentage of predicted DMR that contained a feature was used for “prediction power”. For the final prediction the combined groups of features had the highest impact with 100% accuracy compared to individual features. As observed for individual features, Fig 6, it can be seen that SG transcription factors have above 60% prediction power which is not that high compared to the neutral impact of 50%. However, for DHVPP sequence motifs have over 90% power followed by 70% for transcription factors. When only single features are used for training, their power of prediction is generally lower than when combined. For both the datasets CpG density had a high prediction power rate of 99%. For DHVPP a number of features for example, MOTIF CCGG and GCGC have higher than 90% prediction power, followed by TCGG which has higher than 80% prediction power. All of these motifs were constructed by running the predicted initial DMR sites through a number of motif finding algorithms to find new motif sequences which were used for prediction [23]. Among those highly selected motifs these few performed well and were chosen for the final 134 features for DHVPP sperm dataset.

Once the two step training was completed the trained model was used for a genome-wide prediction. The rat genome was annotated with all the genomic features selected and the learned classifier was applied. Among the initial list of predicted 48K sites for the sperm DHVPP and 28K for somatic SG sites, after selecting only the three or more consecutive sites a final list of 3,233 sites for DHVPP germline cell and 1,502 sites for somatic cell SG remained. There are more sites in the DHVPP in part since this is a combination of five different experiments. In contrast, somatic cell SG datasets involved two individual cell types from the testis and ovaries only and the number of epimutations was less than the germ cell datasets.

The number of specific DMR that localized onto each chromosome for the somatic cell 1,502 sites and germ cell 3,233 sites was found to be comparable between chromosomes, Fig 8A. Chromosome 1 and 2 for both datasets show higher numbers of sites in part due to the size of these chromosomes. A cluster analysis for genomic regions with a statistically significant over-representation of predicted DMR identified a number of clusters on each chromosome, Figs 3 & 4. Previously over-represented differential gene expression near DMR were identified as Epigenetic Control Regions (ECR) [35], similar to Imprinting Control Regions (ICR) [62]. The speculation is these clustered DMR have a role in the epigenetic regulation of gene expression in large regions of 2–5 megabases (S3 Table) [35]. Further work on the functional role of these predicted DMR and the clusters is needed.

Interestingly, the predicted germ cell DMR and somatic DMR were distinct with negligible overlap, Fig 8. In addition, the learned classifiers and the critical genomic features also were different between germ cell and somatic cell DMR. However, the CpG desert feature was common between the predicted DMR datasets. Observations suggest the molecular elements and characteristics of the somatic cell and germ cell DMR are distinct. As different feature sets were used for training for both germ cells and somatic cells the predicted DMR have negligible overlap. Although the CpG density was common and critical for both, the other features were more variable. Since the germ cell DMR are important for the epigenetic transgenerational inheritance of disease and phenotypic variation [1], while the somatic cell DMR are relevant to the gene regulation with specific cell types, it is not surprising that the molecular characteristic of the DMR are distinct. Further work and examples will help elucidate the biological importance of the differences.

A partial validation of the novel machine learning approach and predicted genome-wide germ cell DMR used recently identified sperm DMR not used as training data sets. The transgenerational sperm epimutations from DDT [14] and methoxychlor [13] lineage F3 generation animals were combined and used as a positive validation DMR data set termed MXC-DDT. Since these are independently identified transgenerational sperm DMR they should appear in the transgenerational machine learning predicted genome-wide sperm DMR data set. The analysis showed 100% prediction accuracy of the MXC-DDT DMR being selected by the machine learning algorithm when used as a training set. The MXC-DDT DMR were found to have a 38% overlap with the single sites in comparison with the predicted sperm DMR dataset (Fig 9). This observation helps validate the machine learning approach and predicted genome-wide datasets obtained. However, the incomplete overlap indicates future studies are needed that involve additional training sets to optimize the genome-wide predicted epimutation data set. In contrast, a negative validation data set used a set of transcription factor binding sites that are irrelevant to DMR and had negligible overlap nor selection. For example, the negative validation data set sites generally had high density CpG (less than 42% had low density CpG sites). Although clearly identified non-DMR data sets are difficult to obtain, this negative validation data set used helps support the prediction power and accuracy of the current study. Further validation of the machine learning approach and predicted DMR data set is required, but the initial observations support the utility of the approach and dataset.

## Conclusion

The novel machine learning approach utilized a sequential generalized query based active learning and imbalance class learning on epigenetic data sets. Some studies have applied machine learning to epigenetics [25, 26]. However, the machine learning approach developed can be used to increase the accuracy and efficiency of the prediction of machine learning with any biological dataset or any dataset for that matter. The advantage to this novel sequential machine learning approach is better accuracy through balancing the datasets and then using optimal features to train the classifier and increase efficiency. The current approach used a tandem sequential process, but future improvement could be to combine the active and imbalance learning into a single process. Broader use of this approach is anticipated to improve the specific machine learning tool developed and enhance machine learning applications.

A variety of different environmental exposures [1] have been shown to induce the epigenetic inheritance of disease and phenotypic variation in species ranging from plants, flies, worms, fish, rodents, pigs and humans [1, 11, 43, 63–67]. The germline transmission of altered epigenetic information is the mechanism behind this non-genetic form of inheritance [9]. Differential DNA methylated regions (DMRs) are in part the epigenetic mechanism of epigenetic inheritance [1]. Previous studies have demonstrated the DMRs termed epimutations identified are exposure specific [18] and correlate to later life disease susceptibility [1]. A variety of different disease conditions, behavioral alterations and phenotypic variation is associated with the epigenetic transgenerational inheritance phenomenon [1]. Identification of DMR or epimutations associated with ancestral or early life exposures correlates to later life disease [18]. A number of studies have demonstrated the feasibility of these epigenetic biomarkers that could be used as early stage diagnostics for disease susceptibility [1]. The current study used a novel sequential machine learning approach to predict the potential susceptible DMR and epimutation sites in the genome. This information and datasets can now be used to more effectively identify the patterns or signatures of DMR associated with specific exposures and disease conditions.

In addition to the prediction of the genome-wide DMR and potential epimutations, the novel machine learning tool also provides critical information regarding the essential genomic molecular features of the DMR. The most important was the low density CpG regions or CpG deserts (Fig 5). The evolutionary significance and regulatory role of such regions has been previously discussed [8, 22]. The assumption is the genomic features identified will be highly conserved among species, in particular mammals. Therefore, the developed machine learning tool may be applicable to many species including humans. Although no epigenetic DMR training sets are available yet in humans, the tool may provide a predicted DMR dataset that can be used to facilitate future human epigenetic biomarker identification. This is an important extension of the current study for the future. Therefore, the observations have provided a useful new machine learning approach and tool for computational biology. In addition, valuable new molecular insights and datasets have been provided to help elucidate the environmentally induced epigenetic transgenerational inheritance phenomenon.

## Methods

### Epigenetic Datasets

The epigenetics datasets are from epigenetic transgenerational inheritance experiments and F3 generation sperm or somatic cells from various exposure lineages, including Dioxin [46], Hydrocarbon Jet Fuel [16], Vinclozolin [16, 18, 19, 46], Plastics [15], and Pesticide [12, 15]. The somatic Sertoli cells and Granulosa cell datasets [20, 21] are derived from adult vinclozolin lineage F3 generation somatic cells that influence the onset of testis and ovarian disease, respectively. The datasets for the germ cell and somatic cell DMR sites [54] have differential DNA methylation changes between the F3 generation exposure and control lineages rat cells. These epigenetic data come from investigations of the actions of environmental exposures during fetal gonadal development that induce epigenetic change in the germ line and promote the epigenetic transgenerational inheritance of adult-onset diseases [3]. The Dioxin, Jet Fuel, Vinclozolin, Plastics and Pesticide datasets consist of ancestral environmental exposures of these five compounds individually and are associated with the epigenetic transgenerational inheritance of adult onset diseases. The molecular procedure to identify the DMR was a differential methylated DNA immunoprecipitation (MeDIP) followed by a tiling array analysis (Chip) for a MeDIP-Chip analysis and the details of how each experiment was performed and data was collected is previously described [18, 20, 21]. An additional validation was done using two recently identified sperm DMR data sets. A combination of the DDT [14] and MXC [13] sperm epimutations is used as a positive control (DDT MXC with 76 DMR).

### Active Learning

For active learning each of the datasets used can be described as a collection of examples each containing a number of features X_1_,X_2_…..X_n_ and class label Y. Initially the learner is given a small training set *R* and a set *U* of unlabeled training instances. From this unlabeled training set, the learner can query the Oracle to label these instances. In the following steps we describe the GQAL approach.

1. Initially the learner L is trained on a small set of labeled examples *R*, there is a set *U* of unlabeled training instances, and two separate test sets *T*_*1*_ and *T*_*2*_.
2. The classifier learned by learner *L* is used on the unlabeled training set *U* to find the most uncertain instance [54].
3. GQAL then takes the chosen uncertain instance and finds the most relevant features for that instance and their ranges.
4. The algorithm poses the generalized query to the Oracle, which gives a label and a probability estimation which is the Oracle’s confidence about the query label.
5. GQAL will take this generalized query and match it with existing instances. Such unlabeled instances are labeled and moved from the unlabeled dataset *U* to the labeled training set *R*.
6. The algorithm learns from this updated training set *R* and tests on the set aside test set *T*_*1*_.
7. GQAL goes back to step 2 and repeats this until it reaches a predefined accuracy or iterates a certain number of times.
8. Once learning is complete the final GQAL classifier from learner *L* is evaluated on the set aside test set *T*_*2*_.

The Tree Augmented Naive Bayes (TAN) is used as a base classifier for the GQAL learner. Details of this algorithm is given in the GQAL paper [30].

After running active learning on the entire feature set of 834 features, the features which appeared as don't care or irrelevant features were removed and features that appeared five times or more were selected as the top features for the dataset. This ended up being 149 features for SG and 134 features for DHVPP. The entire list of genomic features is given in S1 Table. They are grouped into CpG information, repeat elements, transcription factors, sequence motifs and mammalian motifs. Once the most important features were chosen, they were used for imbalanced class learning which is the next step in the combined approach.

### Imbalanced Class Learner

The ICL uses a boosting technique called AdaBoost that makes use of the entire dataset. It uses a committee of experts (weighted classifiers) to classify any new instance based on majority voting. For the training initially all instances in the dataset have equal weights. In each iteration AdaBoost increases the weight on the incorrectly classified instances and decreases the weight on the correctly classified instances. After each iteration the classifier which minimizes the error is chosen as a committee expert and used to update all the instances for the next iteration. Similar to GQAL the TAN classifier is used as a base classifier with AdaBoost.

The two-step DMR identification machine learning framework is as shown in Fig 1, starting from the “Dataset” component. Details of each method are presented in earlier reports [30, 31]. In a combined approach first the active learning is used to select the most important features at each iteration and then the imbalanced class learner is used as a boosting method to maximize the accuracy while learning from an imbalanced dataset. This combined approach (GQAL + (TAN+Adaboost)) is a newer technique than other tightly integrated approaches.

Both the GQAL and TAN+AdaBoost approach were trained with 10 fold cross validation with the DHVPP and SG data. The models created from these two training sets were separately tested for validity using the MXC-DDT and Sox9SryTcf21 datasets. Validation results show that both the datasets SG and DHVPP can identify DMR dataset MXC-DDT properly and can identify non-DMR, non-epigenetic dataset Sox9SryTcf21 as non-DMR with some restrictions.

### Clustering

After the potential DMR sites (1,503 for SG and 3,233 for DHVPP) were extracted, further analysis of the data was done to find if these novel potential DMR sites cluster in certain locations in the genome. A previous study with tissue gene expression array data was used in a cluster analysis of transgenerational differentially expressed genes to identify gene clusters with statistically significant over-represented gene expression [35]. These locations were termed Epigenetic Control Regions (ECRs). A similar analysis for DMR sites was done to find whether such ECR regions exist for the predicted epimutation sites. An overlapping sliding window size of 2,000,000 base was used at an interval of 50,000 base to count the number of potential DMR within the sliding windows. Then a Z-test was performed and p-value of 0.05 statistically significant cut-off, including false discovery analysis, was used to find the windows with over-representations of predicted DMR sites. Then consecutive overlapping windows were merged to form the final list of clusters.

### Feature Extraction

The feature extraction included using RepeatMasker, Motif discovery tools and consensus sequences obtained from JASPER and other sources [20]. Features were extracted from the base region, 1k, 5k and 100k upstream and downstream. A non-overlapping region of 1000 bases was used to scan all the chromosomes of the rat to create the testing regions and then features were collected from these regions and around it (having the 1000 bases as a base region). The same features were used for training and testing for each individual dataset.

## Supporting Information

S1 Table Selected features (A) ACL selected features in the germ cell DHVPP final 134 feature list. Up denotes upstream, Dn denotes downstream, features without Up and Dn initial have been extracted from the base region itself. (B) ACL selected features in the somatic cell (SG) (Sertoli-Granulosa) final 149 feature list. Up denotes upstream, Dn denotes downstream, features without Up and Dn initial have been extracted from the base region itself.
(PDF)

S2 Table Predicted (3+ consecutive sites) DMR. (A) Location of sites (3233) germ cell (DHVPP). (B) Location of sites (1503) somatic cell (SG).
(PDF)

S3 Table Clusters from combined datasets and stats (cluster size, number of sites in each cluster). (A) Clusters from germ cell predicted DMRs (from 3+ consecutive sites only) (80 sites). (B) Clusters from somatic cell Sertoli-Granulosa predicted DMRs (from 3+ consecutive sites only) (44 sites).
(PDF)
