# Supplementary material for: Genome-Wide Locations of Potential Epimutations Associated with Environmentally Induced Epigenetic Transgenerational Inheritance of Disease Using a Sequential Machine Learning Prediction Approach
Source: PLoS One. 2015 Nov 16;10(11):e0142274. doi: 10.1371/journal.pone.0142274 (PMC4646459; doi:10.1371/journal.pone.0142274)
Supplement: S1 Table — (A) ACL selected features in the germ cell DHVPP final 134 feature list. Up denotes upstream, Dn denotes downstream, features without Up and Dn initial have been extracted from the base region itself. (B) ACL selected features in the somatic cell (SG) (Sertoli-Granulosa) final 149 feature list. Up denotes upstream, Dn denotes downstream, features without Up and Dn initial have been extracted from the base region itself. (PDF) [file pone.0142274.s001.pdf]

Supplemental Table S1.

(A) ACL selected features in the germ cell DHVPP (Dioxin, Hydrocarbon (Jet Fuel), Vinclozolin, Plastics, Pesticide) final feature list (134). Up denotes upstream, Dn denotes downstream, features without Up and Dn initial have been extracted from the base region itself.

CpG Elements	RepeatElements	Location	TranscriptionFactors	Location	SequenceMotifs	Location	Mammalian Motifs	Location
CpGdensity	A.elements	5kUp, 5kDn, 100kUp	Octob1	100kDn	CACGTG	DMR, 100kDn	MCS10.2	100kDn
	A.elements.count	1kUp	ATF.AC0.2	DMR	CCGG	DMR, 100kDn	MCS10.2.2	100kUp
	Alu.B1	100kUp	ATSequence	DMR	EDM2B1	100kUp, 100kDn	MCS10.2.3	100kUp
	Alu.B1.count	5kDn	ATTTTTTTATTTTTATTTTATTTTTTTTTTTTAAAA	100kUp	GCGC	DMR, 100kDn	MCS10.2.4	100kUp
	DNA.elements.count	100kDn	CCGC+ACOA[GT]G-GG+ACO-GGC	DMR	TCGG	DMR, 100kUp	MCS10.3.1	100kDn
	ERV_classI	1kUp	CTCF.AC0.binding	DMR	TGGAGGGGCAGTCCGGCTCCTGGGGG	DMR	MCS10.4.2	100kUp
	ERV_classI.count	1kUp, 100kUp	Ddit3..Cebpa	DMR			MCS10.7	100kUp
	ERV_classII.count	100kDn	DownMethylation	100kDn			MCS11.0.3	100kDn
	ERVL.count	1kUp, 100kUp,100kDn	E2F1	DMR, 100kDn			MCS11.2.2	100kUp
	ERVL.MaLRs	100kUp	EDM2B1	DMR			MCS11.3.1	100kUp
	HAT.Charlie	5kDn	Foxd3	DMR			MCS11.4	100kUp
	HAT.Charlie.count	5kUp	FOXP1	DMR			MCS11.5	100kUp, 100kDn
	L3.CR1	1kUp	HIF1A..ARNT.AKA.	DMR			MCS11.5.1	100kDn
	LINE2.count	100kUp	InsutorProtein	DMR			MCS11.7	100kUp
	LTR.elements	1kUp, 1kDn	KROX	DMR			MCS11.9	100kDn
	LTR.elements.count	100kDn	MAZ	DMR, 100kUp			MCS12.2	100kUp
	MIRs	5kDn	Nrf2.GABPA	DMR			MCS12.2.2	100kDn
	MIRs.count	1kUp, 100kDn	SOX10	DMR, 100kDn			MCS12.3.2	100kUp
	Simple	1kDn	Sp1	DMR			MCS13.0	100kDn
	SINEs	1kDn	TCTCTGCAG	DMR			MCS13.2.1	100kUp
	SINEs.count	5kUp, 1kDn	TGTCTGCAG	DMR			MCS13.7.2	100kDn
	Total.interspersed.repeats	5kUp, 5kDn, 1kDn	TGTTTGCAG	100kUp			MCS13.9	100kUp
			Zfp423.AKA.	100kDn			MCS14.0	100kUp
			ZNF219	100kDn			MCS14.3.2	100kUp
							MCS15.8	100kDn
							MCS16.1	100kDn
							MCS16.2	100kUp
							MCS17.1.1	100kUp
							MCS17.2	100kDn
							MCS17.3	100kDn
							MCS18.8	100kDn
							MCS21.1.1	100kUp
							MCS22.1	100kDn
							MCS22.5	100kUp, 100kDn
							MCS22.8	100kDn
							MCS23.8	100kUp, 100kDn
							MCS24.3.1	100kUp
							MCS25.2	100kDn
							MCS27.2	100kDn
							MCS30.5	100kDn
							MCS32.3.1	100kDn
							MCS37.4	100kDn
							MCS43.9	100kDn
							MCS47.6	100kDn

							MCS69.5	100kDn
							MCS8.1	100kDn
							MCS9.0	100kUp
							MCS9.5	100kUp
							MCS9.5.1	100kUp, 100kDn
							MCS9.6	100kUp
							MCS9.6.1	100kUp, 100kDn
							MCS9.8	100kUp
							MCS9.8.4	100kDn

(B) ACL selected features in the somatic cell (SG) (Sertoli-Granulosa) final feature list (149). Up denotes upstream, Dn denotes downstream, features without Up and Dn initial have been extracted from the base region itself.

CpG Elements	RepeatElements	Location	TranscriptionFactors	Location	SequenceMotifs	Location	Mammalian Motifs	Location
CpGdensity	A.elements	100kDn	AP2	100kUp	CACGTG	DMR, 100kDn	MCS10.2	100kDn
	A.elements.count	100kDn	ATF.AC0.2	100kUp	TCGG	100kUp, 100kDn	MCS10.2.1	100kDn
	Alu.B1	5kDn	ATSequence	100kDn			MCS10.2.2	100kDn
	Alu.B1.count	5kUp, 5kDn, 100kUp	AZF1	100kUp, 100kDn			MCS10.2.3	100kUp, 100kDn
	B2.B4	5kDn, 100kUp	CHR	DMR, 100kDn			MCS10.2.4	100kDn
	B2.B4.count	100kUp	CREB1	DMR, 100kUp, 100kDn			MCS10.3	100kDn
	ERVL.count	100kUp	CTCF.AC0.binding	100kUp, 100kDn			MCS10.3.1	100kDn
	HAT.Charlie	100kUp	DR.AC0.2	100kDn			MCS10.7	100kUp
	HAT.Charlie.count	100kUp, 100kDn	E2F1	100kUp, 100kDn			MCS10.9	100kUp
	IDS	100kDn	GC	DMR			MCS107.8	100kUp
	IDS.count	100kDn	HIF1A..ARNT.AKA.	100kDn			MCS11.1	100kUp
	LINE1	1kUp, 5kDn	KBS	100kUp			MCS11.1.1	100kUp
	LINE1.count	1kUp	KROX	DMR, 100kUp			MCS11.1.3	100kUp
	LINE2	100kDn	Mafb	DMR			MCS11.3	100kDn
	LINEs	100kUp, 100kDn	MAZ	DMR			MCS11.3.1	100kUp, 100kDn
	LTR.elements.count	100kUp, 100kDn	Methylation	DMR, 100kUp			MCS12.0	100kDn
	MIRs	100kUp	Methylation	100kDn			MCS12.1	100kUp
	MIRs.count	5kUp	Methylation	100kDn			MCS12.2	100kUp
	Simple	100kUp	NFATC2	100kUp, 100kDn			MCS12.2.1	100kUp
	SINEs	5kUp	NFYA	DMR			MCS12.2.2	100kUp
	SINEs.count	5kUp, 5kDn, 100kDn	Nrf2.GABPA	DMR, 100kDn			MCS12.7	100kDn
	Total.interspersed.repeats	1kUp, 5kDn	SOX10	100kDn			MCS12.7.1	100kDn
	A.elements	100kDn	Sp1	100kUp, 100kDn			MCS13.2.1	100kUp
			Sp1.1	DMR, 100kUp			MCS13.9	100kUp
			TGTCTGCAG	100kUp, 100kDn			MCS14.1	100kDn
			USF1.AC0.binding	100kUp, 100kDn			MCS14.3	100kDn
			ZBTB4.AC0.binding	100kDn			MCS14.3.2	100kDn
							MCS14.9	100kDn
							MCS14.9.1	100kDn
							MCS15.0	100kUp
							MCS16.1	100kUp
							MCS17.2	100kDn
							MCS17.4	100kUp, 100kDn
							MCS19.1	100kUp
							MCS19.1.1	100kUp
							MCS19.8	100kDn
							MCS21.6	100kDn
							MCS22.1	100kUp
							MCS22.5	100kUp
							MCS23.4	100kUp, 100kDn
							MCS23.8	100kUp

							MCS24.3.1	100kUp
							MCS25.2	100kUp
							MCS25.7	100kDn
							MCS26.4	100kUp
							MCS30.0	100kDn
							MCS30.5	100kUp, 100kDn
							MCS30.8	100kDn
							MCS32.3.1	100kUp
							MCS33.5	100kDn
							MCS37.3	100kDn
							MCS37.4	100kUp
							MCS40.4	100kDn
							MCS43.9	100kDn
							MCS44.8	100kUp
							MCS46.0	100kUp
							MCS47.6	100kUp
							MCS51.6	100kUp
							MCS64.6	100kUp
							MCS8.1	100kUp
							MCS80.4	100kUp
							MCS9.1	100kDn
							MCS9.1.1	100kUp, 100kDn
							MCS9.8	100kDn
							MCS9.8.1	100kUp
