# Supplementary material for: Genome-Wide Locations of Potential Epimutations Associated with Environmentally Induced Epigenetic Transgenerational Inheritance of Disease Using a Sequential Machine Learning Prediction Approach
Source: PLoS One. 2015 Nov 16;10(11):e0142274. doi: 10.1371/journal.pone.0142274 (PMC4646459; doi:10.1371/journal.pone.0142274)
Supplement: S2 Table — (A) Location of sites (3233) germ cell (DHVPP). (B) Location of sites (1503) somatic cell (SG). (PDF) [file pone.0142274.s002.pdf]

Supplemental Table S2

Predicted DMR/Epimutation Sites

(A) Final list of susceptible DMRs (includes 3+ consecutive sites only) in germ cell (3233)

Potential DMR site	Length	CpG /100bp
chr1:429001-432001	3000	0.5
chr1:2248001-2251001	3000	0.7
chr1:2774364-2778364	4000	0.125
chr1:3983364-3986364	3000	0.66666667
chr1:4073364-4076364	3000	0.76666667
chr1:4379364-4383364	4000	1.075
chr1:4662364-4665364	3000	1.1
chr1:4755364-4761364	6000	0.55
chr1:4854364-4858364	4000	0.375
chr1:5461364-5464364	3000	0.83333333
chr1:5706364-5709364	3000	1.13333333
chr1:5938364-5941364	3000	0.66666667
chr1:6150364-6153364	3000	0.83333333
chr1:6757364-6762364	5000	0.62
chr1:6818364-6821364	3000	0.63333333
chr1:6843364-6846364	3000	1.13333333
chr1:9115364-9118364	3000	1
chr1:9127364-9130364	3000	1.3
chr1:9484364-9487364	3000	0.56666667
chr1:10199364-10202364	3000	0.36666667
chr1:10342364-10345364	3000	0.3
chr1:11547364-11553364	6000	0.28333333
chr1:14526364-14530364	4000	1.325
chr1:14649364-14652364	3000	1.5
chr1:14970364-14973364	3000	1.6
chr1:17003364-17006364	3000	0.56666667
chr1:18263364-18266364	3000	0.8
chr1:18904364-18908364	4000	0.4
chr1:19352364-19355364	3000	0.66666667
chr1:20158364-20162364	4000	0.775
chr1:20273364-20276364	3000	1.2
chr1:20867364-20870364	3000	1.33333333
chr1:20956364-20959364	3000	0.23333333
chr1:22524364-22528364	4000	0.675
chr1:23052364-23057364	5000	0.46
chr1:24126364-24129364	3000	0.93333333
chr1:25458364-25461364	3000	0.5
chr1:27414364-27420364	6000	1.05
chr1:27544364-27547364	3000	0.86666667
chr1:28858364-28862364	4000	0.325
chr1:29211364-29214364	3000	0.46666667
chr1:29983364-29986364	3000	0.4
chr1:30149364-30152364	3000	0.96666667
chr1:31733364-31738364	5000	0.24

chr1:33231364-33234364	3000	0.86666667
chr1:33280364-33283364	3000	1.13333333
chr1:33525364-33531364	6000	0.41666667
chr1:33971364-33975364	4000	0.375
chr1:34123364-34128364	5000	0.5
chr1:34134364-34137364	3000	0.26666667
chr1:34231364-34235364	4000	0.85
chr1:34296364-34301364	5000	0.38
chr1:35597364-35600364	3000	1.03333333
chr1:36202364-36205364	3000	0.9
chr1:37015364-37020364	5000	0.94
chr1:41505862-41508862	3000	1.73333333
chr1:43005862-43009862	4000	1.5
chr1:43032862-43035862	3000	1.23333333
chr1:45212862-45215862	3000	1.4
chr1:45236862-45240862	4000	0.55
chr1:45919862-45922862	3000	1.96666667
chr1:46324862-46327862	3000	1.53333333
chr1:46534862-46537862	3000	1.53333333
chr1:46626862-46633862	7000	0.72857143
chr1:48057862-48060862	3000	1.03333333
chr1:48197862-48200862	3000	1
chr1:48618862-48621862	3000	1.3
chr1:48642862-48645862	3000	0.86666667
chr1:49155862-49158862	3000	0.36666667
chr1:49202862-49206862	4000	0.375
chr1:50450862-50453862	3000	0.53333333
chr1:51114862-51117862	3000	0.26666667
chr1:53183862-53186862	3000	0.56666667
chr1:53429862-53434862	5000	1.28
chr1:53654862-53658862	4000	0.8
chr1:53703862-53706862	3000	0.46666667
chr1:54013862-54017862	4000	0.925
chr1:54262862-54266862	4000	1.025
chr1:54871862-54874862	3000	0.2
chr1:55549862-55552862	3000	0.66666667
chr1:55854862-55858862	4000	0.425
chr1:55932862-55937862	5000	0.58
chr1:56174862-56177862	3000	0.8
chr1:56853862-56856862	3000	0.43333333
chr1:56914862-56917862	3000	0.43333333
chr1:56964862-56967862	3000	0.26666667
chr1:57085862-57088862	3000	0.43333333
chr1:57425862-57428862	3000	0.1
chr1:58616862-58619862	3000	1.06666667
chr1:59363862-59366862	3000	0.7
chr1:59774862-59777862	3000	0.33333333
chr1:60541862-60546862	5000	0.5
chr1:74200549-74204549	4000	0.225
chr1:75581549-75584549	3000	0.6
chr1:77666300-77671300	5000	1.08
chr1:81291561-81294561	3000	1.03333333
chr1:81360561-81363561	3000	0.56666667

chr1:84740637-84743637	3000	0.43333333
chr1:86540444-86543444	3000	1.2
chr1:89644575-89650575	6000	1.23333333
chr1:91342575-91345575	3000	0.9
chr1:91752575-91755575	3000	0.63333333
chr1:92092575-92096575	4000	0.275
chr1:92135575-92139575	4000	0.575
chr1:92307575-92311575	4000	0.9
chr1:92621575-92627575	6000	0.5
chr1:100839655-100842655	3000	0.5
chr1:102372655-102376655	4000	0.5
chr1:103608655-103611655	3000	0.2
chr1:104511655-104514655	3000	0.26666667
chr1:104907655-104910655	3000	0.23333333
chr1:105618655-105621655	3000	0.46666667
chr1:105887655-105891655	4000	0.475
chr1:106383655-106387655	4000	0.475
chr1:107670655-107674655	4000	0.45
chr1:107835655-107838655	3000	0.36666667
chr1:107927655-107931655	4000	0.575
chr1:108071655-108075655	4000	0.425
chr1:108084655-108087655	3000	0.26666667
chr1:108662655-108665655	3000	0.5
chr1:108836655-108839655	3000	1
chr1:109694655-109698655	4000	0.325
chr1:109828655-109833655	5000	0.4
chr1:109989655-109992655	3000	1.3
chr1:110081655-110084655	3000	0.23333333
chr1:110613655-110616655	3000	0.5
chr1:110648655-110651655	3000	0.33333333
chr1:111685655-111688655	3000	0.36666667
chr1:111744655-111747655	3000	0.23333333
chr1:111841655-111844655	3000	0.4
chr1:111877655-111880655	3000	0.16666667
chr1:112792655-112796655	4000	0.25
chr1:112883655-112886655	3000	0.5
chr1:112967655-112970655	3000	0.43333333
chr1:112978655-112981655	3000	0.13333333
chr1:112994655-112997655	3000	0.36666667
chr1:113293655-113296655	3000	0.33333333
chr1:113358655-113362655	4000	0.2
chr1:113526655-113529655	3000	0.63333333
chr1:113579655-113582655	3000	0.53333333
chr1:113737655-113740655	3000	0.53333333
chr1:113753655-113756655	3000	0.36666667
chr1:113803655-113806655	3000	0.23333333
chr1:113870655-113875655	5000	0.36
chr1:113899655-113904655	5000	0.48
chr1:114004655-114007655	3000	0.33333333
chr1:114426655-114429655	3000	0.23333333
chr1:114441655-114444655	3000	0.3
chr1:114476655-114480655	4000	0.3
chr1:116250655-116253655	3000	0.4

chr1:117368655-117371655	3000	0.46666667
chr1:118295655-118298655	3000	1.03333333
chr1:119005655-119009655	4000	0.8
chr1:119384655-119387655	3000	0.8
chr1:119499655-119502655	3000	1.03333333
chr1:121078877-121082877	4000	1.075
chr1:123549877-123552877	3000	0.66666667
chr1:123820877-123823877	3000	0.83333333
chr1:124634877-124637877	3000	0.63333333
chr1:125667929-125670929	3000	1.36666667
chr1:125875929-125881929	6000	0.36666667
chr1:126629929-126633929	4000	0.6
chr1:127778929-127782929	4000	0.525
chr1:128229929-128233929	4000	1.125
chr1:129495929-129499929	4000	1.075
chr1:129548929-129551929	3000	0.66666667
chr1:130032929-130035929	3000	0.9
chr1:130217929-130220929	3000	0.73333333
chr1:131750752-131753752	3000	0.33333333
chr1:133129752-133132752	3000	0.33333333
chr1:133364752-133367752	3000	0.36666667
chr1:139003759-139007759	4000	0.55
chr1:139494759-139499759	5000	0.56
chr1:139527759-139530759	3000	0.5
chr1:142452187-142455187	3000	0.46666667
chr1:143050187-143054187	4000	0.525
chr1:143767187-143770187	3000	0.2
chr1:143978187-143982187	4000	0.525
chr1:144613187-144616187	3000	0.46666667
chr1:144798187-144801187	3000	0.73333333
chr1:144845187-144850187	5000	0.72
chr1:145344187-145348187	4000	0.675
chr1:145594187-145598187	4000	0.45
chr1:147825042-147828042	3000	0.46666667
chr1:151018042-151022042	4000	0.55
chr1:151320042-151325042	5000	0.3
chr1:152375042-152378042	3000	0.3
chr1:163688846-163692846	4000	0.225
chr1:166934550-166937550	3000	0.4
chr1:171642550-171646550	4000	1.05
chr1:172600782-172603782	3000	0.43333333
chr1:182691061-182694061	3000	0.5
chr1:182705061-182708061	3000	0.36666667
chr1:182878061-182881061	3000	0.7
chr1:183743061-183746061	3000	0.46666667
chr1:183822061-183825061	3000	0.66666667
chr1:184073061-184077061	4000	0.625
chr1:184444061-184447061	3000	1.3
chr1:191489919-191493919	4000	0.6
chr1:193835919-193838919	3000	0.7
chr1:194338919-194342919	4000	0.575
chr1:195714919-195718919	4000	0.75
chr1:195787919-195791919	4000	0.425

chr1:195833919-195837919	4000	0.775
chr1:197327432-197330432	3000	1.03333333
chr1:197394432-197397432	3000	0.6
chr1:197617432-197623432	6000	0.75
chr1:197889432-197892432	3000	0.26666667
chr1:198117432-198120432	3000	0.43333333
chr1:198318432-198321432	3000	0.33333333
chr1:199568432-199571432	3000	1.03333333
chr1:202030803-202034803	4000	0.65
chr1:210832677-210835677	3000	0.33333333
chr1:214268321-214271321	3000	0.43333333
chr1:214305321-214308321	3000	0.33333333
chr1:215242080-215245080	3000	0.2
chr1:216845879-216848879	3000	0.4
chr1:217285879-217289879	4000	0.675
chr1:217319879-217322879	3000	0.33333333
chr1:217935879-217938879	3000	0.6
chr1:217983879-217987879	4000	0.3
chr1:218076879-218079879	3000	0.86666667
chr1:218151879-218156879	5000	0.62
chr1:218508879-218515879	7000	0.68571429
chr1:218815879-218819879	4000	1.1
chr1:218842879-218845879	3000	0.73333333
chr1:218848879-218851879	3000	0.46666667
chr1:218901879-218910879	9000	0.45555556
chr1:218959879-218962879	3000	0.53333333
chr1:219042879-219045879	3000	0.23333333
chr1:219451879-219455879	4000	0.65
chr1:221618879-221621879	3000	0.63333333
chr1:222019879-222022879	3000	0.4
chr1:222819879-222822879	3000	0.73333333
chr1:222901879-222904879	3000	0.43333333
chr1:222911879-222914879	3000	0.43333333
chr1:222929879-222932879	3000	0.56666667
chr1:223901879-223905879	4000	0.85
chr1:224124879-224131879	7000	0.78571429
chr1:224246879-224249879	3000	0.6
chr1:224306879-224309879	3000	0.33333333
chr1:224703879-224707879	4000	0.625
chr1:224715879-224718879	3000	1
chr1:225208879-225211879	3000	0.5
chr1:226866879-226870879	4000	0.75
chr1:227012879-227016879	4000	0.525
chr1:228061879-228064879	3000	1.73333333
chr1:229619879-229623879	4000	1.125
chr1:230283056-230286056	3000	0.5
chr1:230381056-230384056	3000	1.13333333
chr1:230613056-230618056	5000	0.5
chr1:230795056-230804056	9000	0.3
chr1:231836056-231839056	3000	0.5
chr1:235850745-235855745	5000	0.68
chr1:237049745-237052745	3000	0.7
chr1:237312745-237315745	3000	0.76666667

chr1:239100745-239104745	4000	0.725
chr1:239812745-239816745	4000	0.75
chr1:239878745-239883745	5000	0.64
chr1:244688808-244691808	3000	0.96666667
chr1:245364808-245368808	4000	1.1
chr1:247662808-247665808	3000	1.1
chr1:247848808-247851808	3000	1.23333333
chr1:247860808-247864808	4000	0.475
chr1:253596776-253599776	3000	0.7
chr1:253632776-253635776	3000	0.93333333
chr1:253663776-253666776	3000	1.46666667
chr1:254570776-254575776	5000	0.82
chr1:254985776-254988776	3000	0.8
chr1:255025776-255028776	3000	0.23333333
chr1:255231776-255234776	3000	0.8
chr1:255957776-255961776	4000	0.85
chr1:256976776-256980776	4000	0.375
chr1:257739776-257743776	4000	0.35
chr1:258394776-258397776	3000	1.23333333
chr1:258443776-258446776	3000	0.5
chr1:258941776-258944776	3000	2.06666667
chr1:260567846-260570846	3000	0.86666667
chr1:261100846-261104846	4000	0.9
chr1:263310846-263315846	5000	1.78
chr1:265280846-265283846	3000	0.83333333
chr1:267073846-267077846	4000	0.35
chr10:2487219-2491219	4000	1.475
chr10:2537219-2540219	3000	0.9
chr10:2777219-2780219	3000	0.5
chr10:2817219-2820219	3000	0.73333333
chr10:2881219-2884219	3000	0.43333333
chr10:3553219-3556219	3000	0.63333333
chr10:3727219-3731219	4000	1.525
chr10:8228219-8231219	3000	0.93333333
chr10:14687487-14690487	3000	1.23333333
chr10:18544714-18547714	3000	1.26666667
chr10:23010714-23013714	3000	0.33333333
chr10:24128714-24131714	3000	0.43333333
chr10:25048714-25051714	3000	0.6
chr10:25395714-25398714	3000	0.83333333
chr10:26476714-26479714	3000	0.36666667
chr10:27859714-27862714	3000	0.33333333
chr10:28040714-28043714	3000	0.6
chr10:28515714-28518714	3000	0.86666667
chr10:30563714-30566714	3000	1.1
chr10:30588714-30591714	3000	0.73333333
chr10:30598714-30602714	4000	0.575
chr10:33089714-33092714	3000	0.76666667
chr10:45042550-45046550	4000	0.75
chr10:49226538-49229538	3000	1.3
chr10:49449538-49454538	5000	0.94
chr10:49564538-49567538	3000	0.66666667
chr10:49584538-49587538	3000	0.43333333

chr10:49744538-49747538	3000	1.06666667
chr10:49924538-49928538	4000	0.825
chr10:49986538-49990538	4000	0.6
chr10:51080538-51083538	3000	0.73333333
chr10:51394538-51397538	3000	0.6
chr10:51401538-51404538	3000	1.46666667
chr10:51408538-51411538	3000	1.16666667
chr10:52421538-52424538	3000	0.4
chr10:52764538-52767538	3000	0.43333333
chr10:52848538-52851538	3000	0.36666667
chr10:53088538-53091538	3000	0.53333333
chr10:53141538-53144538	3000	0.5
chr10:53221538-53224538	3000	0.83333333
chr10:53368538-53371538	3000	0.56666667
chr10:58148804-58151804	3000	0.33333333
chr10:58737804-58740804	3000	0.56666667
chr10:71985127-71989127	4000	1.05
chr10:78239106-78243106	4000	0.875
chr10:79463106-79466106	3000	0.56666667
chr10:80001106-80004106	3000	0.86666667
chr10:80692106-80696106	4000	0.5
chr10:88760611-88763611	3000	0.76666667
chr10:100239097-100242097	3000	1.36666667
chr10:100666097-100669097	3000	1.3
chr11:404001-407001	3000	0.26666667
chr11:1936001-1939001	3000	0.46666667
chr11:1962001-1965001	3000	0.66666667
chr11:1967001-1970001	3000	0.63333333
chr11:2079001-2082001	3000	0.46666667
chr11:2115001-2118001	3000	0.4
chr11:2606001-2609001	3000	0.26666667
chr11:3585001-3588001	3000	0.16666667
chr11:3841001-3848001	7000	0.75714286
chr11:4696001-4699001	3000	0.73333333
chr11:6564456-6568456	4000	0.25
chr11:6785456-6793456	8000	0.325
chr11:6885456-6889456	4000	0.4
chr11:7473456-7477456	4000	0.725
chr11:7687456-7690456	3000	0.4
chr11:9003456-9006456	3000	0.7
chr11:9129456-9135456	6000	0.41666667
chr11:9871456-9874456	3000	0.66666667
chr11:9928456-9931456	3000	0.53333333
chr11:10038456-10041456	3000	0.53333333
chr11:10074456-10077456	3000	0.3
chr11:11273456-11277456	4000	0.9
chr11:12338456-12341456	3000	0.7
chr11:12454456-12458456	4000	0.45
chr11:14437456-14440456	3000	0.36666667
chr11:15041456-15044456	3000	0.53333333
chr11:15058456-15061456	3000	0.5
chr11:15682456-15687456	5000	0.76
chr11:15697456-15701456	4000	0.925

chr11:15726456-15729456	3000	0.53333333
chr11:16015456-16018456	3000	0.4
chr11:16941456-16945456	4000	0.525
chr11:17439456-17442456	3000	1
chr11:18658625-18661625	3000	0.16666667
chr11:18756625-18759625	3000	0.43333333
chr11:19417625-19420625	3000	0.93333333
chr11:20691191-20695191	4000	0.725
chr11:22576191-22579191	3000	1.26666667
chr11:23322191-23327191	5000	0.4
chr11:23513191-23516191	3000	0.43333333
chr11:23536191-23539191	3000	0.43333333
chr11:24101191-24104191	3000	0.7
chr11:24929191-24934191	5000	0.56
chr11:25160191-25165191	5000	0.8
chr11:26768191-26771191	3000	0.83333333
chr11:26909191-26912191	3000	0.53333333
chr11:27786191-27791191	5000	0.52
chr11:28198191-28203191	5000	0.52
chr11:28999191-29003191	4000	0.3
chr11:29210191-29214191	4000	0.675
chr11:29233191-29237191	4000	0.45
chr11:29394191-29397191	3000	0.8
chr11:29442191-29445191	3000	0.56666667
chr11:29499191-29502191	3000	1
chr11:30549191-30554191	5000	0.76
chr11:30565191-30568191	3000	1.83333333
chr11:31397191-31401191	4000	0.9
chr11:32310191-32313191	3000	1.9
chr11:36878191-36881191	3000	1.23333333
chr11:37038191-37043191	5000	1.52
chr11:38444191-38447191	3000	0.36666667
chr11:39013191-39017191	4000	0.8
chr11:40736191-40739191	3000	0.36666667
chr11:41053191-41056191	3000	0.76666667
chr11:41131191-41134191	3000	0.96666667
chr11:41910191-41913191	3000	0.6
chr11:42605191-42608191	3000	0.43333333
chr11:43450191-43454191	4000	0.625
chr11:43760191-43763191	3000	0.53333333
chr11:44766191-44769191	3000	0.46666667
chr11:45121191-45124191	3000	0.8
chr11:45129191-45132191	3000	0.7
chr11:45205191-45208191	3000	0.93333333
chr11:46221191-46224191	3000	0.43333333
chr11:48694191-48697191	3000	0.43333333
chr11:49965191-49968191	3000	0.93333333
chr11:49973191-49977191	4000	0.3
chr11:50785191-50788191	3000	0.63333333
chr11:51235191-51238191	3000	1.26666667
chr11:51315191-51318191	3000	0.7
chr11:52606191-52609191	3000	0.9
chr11:53334191-53339191	5000	0.46

chr11:54097191-54100191	3000	0.63333333
chr11:54224191-54227191	3000	0.46666667
chr11:54345191-54348191	3000	0.5
chr11:54621191-54625191	4000	0.3
chr11:54717191-54721191	4000	0.4
chr11:56220800-56223800	3000	0.86666667
chr11:56847800-56850800	3000	0.5
chr11:59939306-59943306	4000	0.8
chr11:60531306-60534306	3000	0.36666667
chr11:61193306-61196306	3000	0.53333333
chr11:61533306-61537306	4000	0.65
chr11:61597306-61602306	5000	0.62
chr11:62644306-62647306	3000	0.5
chr11:65219306-65222306	3000	0.2
chr11:65528306-65531306	3000	1.5
chr11:73192306-73196306	4000	0.8
chr11:73322306-73325306	3000	0.6
chr11:73468306-73473306	5000	0.66
chr11:73544306-73548306	4000	0.5
chr11:74078306-74081306	3000	0.93333333
chr11:74519306-74523306	4000	0.775
chr11:74538306-74541306	3000	0.8
chr11:74542306-74545306	3000	1.43333333
chr11:74666306-74669306	3000	1.2
chr11:75330306-75333306	3000	0.8
chr11:75373306-75376306	3000	0.76666667
chr11:75432306-75435306	3000	1.16666667
chr11:75735306-75738306	3000	0.53333333
chr11:76597495-76601495	4000	0.925
chr11:76665495-76668495	3000	0.63333333
chr11:78754495-78757495	3000	0.53333333
chr11:79165495-79168495	3000	0.83333333
chr11:79320495-79323495	3000	0.43333333
chr11:79332495-79335495	3000	0.63333333
chr11:79387495-79390495	3000	0.66666667
chr11:80516495-80519495	3000	0.86666667
chr11:80521495-80526495	5000	1.26
chr11:80590495-80594495	4000	1.15
chr11:81750495-81754495	4000	1.4
chr11:82165495-82169495	4000	0.3
chr11:82236495-82240495	4000	0.375
chr11:83287228-83290228	3000	1.33333333
chr11:83391228-83396228	5000	0.74
chr11:83447228-83450228	3000	0.56666667
chr11:83713458-83717458	4000	0.475
chr11:84245458-84251458	6000	0.86666667
chr11:86447794-86450794	3000	0.43333333
chr11:86552794-86555794	3000	0.56666667
chr12:15278837-15288837	10000	1.8
chr12:15535837-15539837	4000	1.025
chr12:15682837-15691837	9000	1.73333333
chr12:15869837-15872837	3000	1.73333333
chr12:16266837-16275837	9000	1.41111111

chr12:17498864-17503864	5000	0.96
chr12:17661864-17665864	4000	0.925
chr12:17669864-17672864	3000	0.86666667
chr12:18002864-18005864	3000	0.36666667
chr12:18070864-18073864	3000	0.66666667
chr12:18618864-18621864	3000	0.63333333
chr12:19536864-19549864	13000	1.50769231
chr12:19854329-19858329	4000	2.075
chr12:20331329-20336329	5000	0.52
chr12:20348329-20351329	3000	0.43333333
chr12:20887329-20890329	3000	0.83333333
chr12:21065303-21075303	10000	1.38
chr12:21236303-21252303	16000	0.7875
chr12:21253303-21262303	9000	0.74444444
chr12:21502303-21514303	12000	0.95
chr12:21745303-21751303	6000	1.81666667
chr12:22054303-22057303	3000	0.7
chr12:22065303-22069303	4000	0.875
chr12:22447303-22453303	6000	1.81666667
chr12:22544303-22562303	18000	1.65
chr12:22608303-22612303	4000	1.3
chr12:22764303-22775303	11000	1.78181818
chr12:22884303-22899303	15000	1.52666667
chr12:22910303-22917303	7000	0.94285714
chr12:22983303-23002303	19000	1.33684211
chr12:23270303-23275303	5000	1.32
chr12:23370303-23376303	6000	1.53333333
chr12:23495303-23503303	8000	1.9125
chr12:24059303-24063303	4000	1.1
chr12:24322303-24326303	4000	0.6
chr12:24350303-24354303	4000	0.775
chr12:24361303-24366303	5000	0.94
chr12:24652303-24657303	5000	0.88
chr12:24904303-24908303	4000	0.65
chr12:30908483-30911483	3000	0.63333333
chr12:32236483-32248483	12000	1.56666667
chr12:32455483-32462483	7000	1.62857143
chr12:32546483-32549483	3000	1.5
chr12:32557483-32560483	3000	1.73333333
chr12:32629483-32633483	4000	2.075
chr12:32802483-32810483	8000	2
chr12:32848483-32855483	7000	1.71428571
chr12:32959483-32962483	3000	2.23333333
chr12:33043483-33046483	3000	1.5
chr12:33173483-33176483	3000	5.36666667
chr12:33221483-33228483	7000	1.67142857
chr12:33409483-33425483	16000	1.6125
chr12:33612929-33621929	9000	2.05555556
chr12:34452929-34461929	9000	2.12222222
chr12:34650929-34664929	14000	1.56428571
chr12:35177929-35183929	6000	1.2
chr12:35497929-35504929	7000	1.4
chr12:35598929-35610929	12000	1.49166667

chr12:36178929-36198929	20000	1.4
chr12:37124929-37128929	4000	1.975
chr12:37302929-37305929	3000	1.43333333
chr12:37955929-37958929	3000	1.9
chr12:37987929-37990929	3000	2.1
chr12:38379929-38385929	6000	1.55
chr12:38883929-38886929	3000	1.23333333
chr12:38887929-38890929	3000	1.43333333
chr12:39468929-39473929	5000	2.46
chr12:39981929-39984929	3000	2.06666667
chr12:40146929-40149929	3000	1.43333333
chr12:45768806-45771806	3000	1.56666667
chr12:45811806-45815806	4000	0.85
chr12:46165806-46169806	4000	1.125
chr13:1032001-1035001	3000	0.23333333
chr13:1242001-1246001	4000	0.225
chr13:1700001-1703001	3000	0.26666667
chr13:1979001-1983001	4000	0.3
chr13:2550001-2553001	3000	0.36666667
chr13:3593001-3596001	3000	0.73333333
chr13:4073001-4076001	3000	0.5
chr13:4129001-4132001	3000	0.63333333
chr13:4703001-4707001	4000	0.525
chr13:5661001-5665001	4000	0.475
chr13:5748001-5752001	4000	0.325
chr13:6062001-6065001	3000	0.26666667
chr13:7395001-7399001	4000	0.4
chr13:7619001-7622001	3000	0.86666667
chr13:8793001-8796001	3000	0.36666667
chr13:8827001-8830001	3000	0.56666667
chr13:11071001-11074001	3000	0.46666667
chr13:11099001-11102001	3000	0.56666667
chr13:11115001-11118001	3000	0.63333333
chr13:11124001-11128001	4000	0.525
chr13:13699001-13702001	3000	0.4
chr13:13986001-13990001	4000	0.275
chr13:14020001-14023001	3000	0.46666667
chr13:14117001-14120001	3000	0.5
chr13:14477001-14480001	3000	2.76666667
chr13:14986001-14989001	3000	0.36666667
chr13:15870001-15874001	4000	0.5
chr13:17825001-17828001	3000	0.63333333
chr13:20091001-20094001	3000	0.13333333
chr13:20612001-20617001	5000	0.46
chr13:20766001-20769001	3000	0.33333333
chr13:20792001-20795001	3000	0.3
chr13:20911001-20914001	3000	0.26666667
chr13:21121001-21124001	3000	0.33333333
chr13:21247001-21250001	3000	0.6
chr13:21594001-21598001	4000	0.5
chr13:21792001-21795001	3000	0.26666667
chr13:22039001-22042001	3000	0.46666667
chr13:22051001-22055001	4000	0.15

chr13:22300001-22303001	3000	0.16666667
chr13:23885001-23889001	4000	0.65
chr13:24614001-24617001	3000	0.33333333
chr13:24897001-24900001	3000	0.23333333
chr13:24980001-24985001	5000	0.24
chr13:25066001-25069001	3000	0.4
chr13:25568001-25571001	3000	0.26666667
chr13:25780001-25783001	3000	0.36666667
chr13:26510001-26513001	3000	0.4
chr13:26580001-26583001	3000	0.7
chr13:27628001-27631001	3000	0.2
chr13:29426001-29429001	3000	0.43333333
chr13:29955001-29958001	3000	0.53333333
chr13:30139001-30142001	3000	0.3
chr13:35492001-35495001	3000	0.53333333
chr13:35521001-35525001	4000	0.275
chr13:43563001-43566001	3000	1.03333333
chr13:43578001-43581001	3000	0.46666667
chr13:43845001-43848001	3000	1.16666667
chr13:45547796-45551796	4000	1.35
chr13:50430711-50433711	3000	1.16666667
chr13:50634711-50638711	4000	0.675
chr13:50965711-50968711	3000	0.96666667
chr13:51162711-51165711	3000	0.6
chr13:51675711-51678711	3000	0.93333333
chr13:51949711-51952711	3000	0.83333333
chr13:52504711-52507711	3000	1.5
chr13:52679711-52683711	4000	1.075
chr13:53100711-53103711	3000	0.26666667
chr13:54858711-54861711	3000	0.36666667
chr13:55132711-55135711	3000	0.3
chr13:55774711-55777711	3000	0.26666667
chr13:57833711-57836711	3000	0.96666667
chr13:58761711-58764711	3000	0.7
chr13:61486711-61489711	3000	0.86666667
chr13:61610711-61614711	4000	0.4
chr13:62039711-62043711	4000	0.275
chr13:62183711-62190711	7000	0.37142857
chr13:62699711-62702711	3000	0.3
chr13:64000711-64003711	3000	1.16666667
chr13:64087711-64090711	3000	0.5
chr13:65747711-65753711	6000	0.5
chr13:67350711-67354711	4000	2.475
chr13:67496711-67499711	3000	1.6
chr13:73012978-73015978	3000	1.16666667
chr13:73408978-73411978	3000	0.76666667
chr13:73452978-73455978	3000	0.66666667
chr13:73614978-73617978	3000	0.93333333
chr13:73834978-73837978	3000	0.36666667
chr13:74396978-74399978	3000	1.36666667
chr13:74448978-74453978	5000	1.24
chr13:74476978-74479978	3000	0.5
chr13:74540978-74543978	3000	0.56666667

chr13:75132978-75135978	3000	0.7
chr13:75264978-75267978	3000	0.4
chr13:75409978-75413978	4000	0.65
chr13:77303978-77307978	4000	0.575
chr13:77490978-77494978	4000	0.825
chr13:78188978-78191978	3000	0.9
chr13:79866978-79869978	3000	0.83333333
chr13:80477978-80480978	3000	0.76666667
chr13:80774978-80778978	4000	0.475
chr13:83708978-83711978	3000	0.86666667
chr13:85205346-85209346	4000	0.725
chr13:85407346-85411346	4000	1
chr13:85552346-85555346	3000	0.93333333
chr13:88632175-88638175	6000	1.76666667
chr13:88644175-88647175	3000	1.13333333
chr13:89070175-89073175	3000	0.8
chr13:89229175-89232175	3000	0.66666667
chr13:89993444-89996444	3000	0.53333333
chr13:90197444-90201444	4000	0.625
chr13:90916444-90919444	3000	0.9
chr13:98664444-98670444	6000	1.2
chr13:98737444-98741444	4000	0.75
chr13:98827444-98830444	3000	1.46666667
chr13:99773291-99777291	4000	1.15
chr13:101704291-101707291	3000	0.83333333
chr13:101718291-101721291	3000	1.1
chr13:105836291-105839291	3000	0.6
chr13:108414850-108417850	3000	1.33333333
chr13:108453850-108456850	3000	0.86666667
chr13:108855850-108858850	3000	0.56666667
chr13:109512850-109515850	3000	1.3
chr13:109580850-109583850	3000	0.33333333
chr13:109619850-109622850	3000	0.76666667
chr13:109924850-109927850	3000	0.3
chr13:110188850-110191850	3000	0.26666667
chr13:110375850-110379850	4000	0.4
chr14:789171-792171	3000	0.26666667
chr14:914171-917171	3000	0.56666667
chr14:4496512-4500512	4000	0.55
chr14:6063512-6066512	3000	1
chr14:8746512-8749512	3000	1.8
chr14:9743512-9746512	3000	0.66666667
chr14:9822512-9825512	3000	1.83333333
chr14:9876512-9880512	4000	0.75
chr14:11454844-11457844	3000	1.1
chr14:12199844-12202844	3000	0.8
chr14:12289844-12294844	5000	0.6
chr14:12628844-12632844	4000	0.925
chr14:13603844-13606844	3000	0.33333333
chr14:14213844-14221844	8000	1.6375
chr14:14730844-14733844	3000	0.36666667
chr14:16310844-16313844	3000	1.13333333
chr14:18368844-18371844	3000	1.2

chr14:19202844-19205844	3000	0.96666667
chr14:20034844-20037844	3000	0.6
chr14:23249844-23255844	6000	0.6
chr14:24238844-24242844	4000	0.325
chr14:25161844-25164844	3000	0.4
chr14:25456844-25459844	3000	0.53333333
chr14:25541844-25544844	3000	0.66666667
chr14:26361844-26364844	3000	0.73333333
chr14:26812844-26815844	3000	0.6
chr14:26855844-26858844	3000	0.4
chr14:27064844-27067844	3000	0.56666667
chr14:27270844-27273844	3000	0.46666667
chr14:27274844-27277844	3000	0.26666667
chr14:27283844-27286844	3000	0.63333333
chr14:27477844-27482844	5000	0.36
chr14:27634844-27637844	3000	0.6
chr14:27902844-27905844	3000	0.86666667
chr14:27996844-27999844	3000	0.36666667
chr14:28145844-28148844	3000	0.63333333
chr14:28418844-28422844	4000	0.475
chr14:28589844-28592844	3000	0.63333333
chr14:30594844-30598844	4000	0.275
chr14:30685844-30688844	3000	0.26666667
chr14:31093844-31096844	3000	0.6
chr14:31315844-31318844	3000	0.4
chr14:32394844-32397844	3000	0.46666667
chr14:32684844-32687844	3000	0.76666667
chr14:32689844-32693844	4000	0.525
chr14:37145617-37148617	3000	1.16666667
chr14:38217704-38220704	3000	0.8
chr14:38532704-38536704	4000	0.6
chr14:38817704-38821704	4000	0.25
chr14:39209704-39212704	3000	0.53333333
chr14:39368704-39372704	4000	0.6
chr14:39446704-39449704	3000	0.46666667
chr14:39761704-39764704	3000	0.33333333
chr14:39948704-39951704	3000	0.9
chr14:42853704-42857704	4000	0.775
chr14:43136704-43139704	3000	1.4
chr14:46574901-46577901	3000	1.06666667
chr14:47835901-47839901	4000	0.35
chr14:50836901-50840901	4000	0.2
chr14:52118901-52121901	3000	0.3
chr14:54412901-54416901	4000	0.25
chr14:54661901-54664901	3000	0.66666667
chr14:54691901-54694901	3000	0.5
chr14:54889901-54892901	3000	0.2
chr14:55111901-55114901	3000	0.4
chr14:55272901-55275901	3000	0.3
chr14:55566901-55569901	3000	0.23333333
chr14:56066901-56070901	4000	0.525
chr14:57432901-57435901	3000	0.76666667
chr14:57575901-57579901	4000	0.55

chr14:58006901-58009901	3000	0.33333333
chr14:58389901-58392901	3000	0.3
chr14:60157901-60160901	3000	0.56666667
chr14:60506901-60509901	3000	0.43333333
chr14:60531901-60535901	4000	0.375
chr14:61351901-61354901	3000	0.7
chr14:61501901-61506901	5000	0.86
chr14:61549901-61552901	3000	0.43333333
chr14:62034901-62037901	3000	1.03333333
chr14:62115901-62119901	4000	0.4
chr14:63159901-63164901	5000	0.64
chr14:63208901-63211901	3000	1.2
chr14:63217901-63220901	3000	1.16666667
chr14:64599901-64605901	6000	0.71666667
chr14:65718901-65727901	9000	0.63333333
chr14:65906901-65910901	4000	0.825
chr14:66120901-66123901	3000	1
chr14:66251901-66254901	3000	0.43333333
chr14:66276901-66281901	5000	0.92
chr14:66445901-66448901	3000	0.5
chr14:66980901-66983901	3000	0.26666667
chr14:67638901-67641901	3000	0.66666667
chr14:68198901-68201901	3000	0.53333333
chr14:68547901-68550901	3000	0.4
chr14:70124901-70127901	3000	0.66666667
chr14:70529901-70533901	4000	0.6
chr14:71282997-71285997	3000	0.36666667
chr14:71982997-71986997	4000	0.875
chr14:72353985-72358985	5000	0.88
chr14:72757985-72760985	3000	0.63333333
chr14:72767985-72770985	3000	0.63333333
chr14:72892985-72895985	3000	0.76666667
chr14:73396985-73399985	3000	0.53333333
chr14:73701985-73704985	3000	0.66666667
chr14:73771985-73775985	4000	0.425
chr14:74267985-74273985	6000	0.56666667
chr14:74279985-74283985	4000	0.575
chr14:74919985-74922985	3000	0.4
chr14:75258985-75264985	6000	0.55
chr14:75404985-75407985	3000	0.46666667
chr14:76332985-76335985	3000	0.36666667
chr14:76449985-76453985	4000	0.675
chr14:76454985-76457985	3000	0.7
chr14:76613985-76616985	3000	0.96666667
chr14:76793985-76797985	4000	0.3
chr14:76852985-76855985	3000	1.76666667
chr14:77132985-77135985	3000	0.6
chr14:77656985-77659985	3000	1.16666667
chr14:78062946-78065946	3000	0.66666667
chr14:78955946-78960946	5000	0.42
chr14:79439946-79443946	4000	1.075
chr14:79500946-79506946	6000	1.43333333
chr14:80805946-80808946	3000	1.06666667

chr14:81115946-81119946	4000	1.025
chr14:83028836-83033836	5000	0.76
chr14:87735747-87738747	3000	1.56666667
chr14:87755747-87759747	4000	1.325
chr14:87983747-87987747	4000	0.375
chr14:88112747-88115747	3000	1.26666667
chr14:88477747-88481747	4000	0.175
chr14:88510747-88513747	3000	0.3
chr14:88669747-88672747	3000	0.73333333
chr14:89265747-89268747	3000	0.53333333
chr14:89717747-89720747	3000	0.63333333
chr14:90508747-90511747	3000	0.6
chr14:91107747-91110747	3000	0.6
chr14:91531747-91535747	4000	0.45
chr14:92930747-92934747	4000	1.425
chr14:92947747-92951747	4000	0.65
chr14:93394747-93397747	3000	0.96666667
chr14:93427747-93431747	4000	0.65
chr14:93445747-93449747	4000	0.55
chr14:93506747-93509747	3000	0.83333333
chr14:93746747-93749747	3000	0.7
chr14:94039747-94042747	3000	0.56666667
chr14:94458747-94461747	3000	0.53333333
chr14:95219747-95223747	4000	0.5
chr14:95784747-95790747	6000	0.48333333
chr14:96317747-96320747	3000	0.43333333
chr14:96458747-96461747	3000	0.43333333
chr14:97450747-97454747	4000	1.15
chr14:97746747-97750747	4000	1.35
chr14:97809747-97812747	3000	0.46666667
chr14:98801632-98804632	3000	0.56666667
chr14:98840632-98843632	3000	0.5
chr14:99092632-99095632	3000	0.36666667
chr14:100010632-100013632	3000	0.96666667
chr14:100189632-100192632	3000	0.93333333
chr14:102510271-102513271	3000	0.86666667
chr14:105468879-105471879	3000	0.46666667
chr14:107155879-107160879	5000	0.64
chr14:108731879-108735879	4000	0.25
chr14:108764879-108767879	3000	0.7
chr15:960001-963001	3000	0.33333333
chr15:1841001-1845001	4000	0.9
chr15:5256911-5259911	3000	0.3
chr15:5491911-5494911	3000	0.76666667
chr15:5704663-5708663	4000	1.75
chr15:7006663-7009663	3000	0.53333333
chr15:7789663-7792663	3000	0.33333333
chr15:8215663-8218663	3000	0.4
chr15:8252663-8255663	3000	0.53333333
chr15:8283663-8286663	3000	0.7
chr15:8486663-8490663	4000	0.75
chr15:8580663-8583663	3000	1.8
chr15:9702663-9707663	5000	1.38

chr15:10229663-10232663	3000	0.46666667
chr15:11181663-11186663	5000	0.52
chr15:11710663-11713663	3000	0.6
chr15:12373663-12376663	3000	0.33333333
chr15:13144663-13148663	4000	0.3
chr15:17504760-17507760	3000	1.13333333
chr15:17572760-17576760	4000	0.525
chr15:18123760-18126760	3000	0.56666667
chr15:18250760-18253760	3000	0.83333333
chr15:19386760-19389760	3000	0.43333333
chr15:19657760-19660760	3000	0.5
chr15:19725760-19728760	3000	0.46666667
chr15:20237760-20240760	3000	0.73333333
chr15:21015760-21018760	3000	1.1
chr15:21729471-21732471	3000	1.16666667
chr15:22031471-22034471	3000	0.46666667
chr15:24125471-24128471	3000	1.76666667
chr15:24342471-24345471	3000	1.8
chr15:26603471-26608471	5000	0.34
chr15:28432794-28435794	3000	0.83333333
chr15:28908794-28911794	3000	0.63333333
chr15:29038794-29041794	3000	0.46666667
chr15:29201794-29205794	4000	0.5
chr15:29274794-29277794	3000	0.33333333
chr15:29467794-29470794	3000	0.53333333
chr15:29586794-29589794	3000	0.26666667
chr15:30402794-30405794	3000	0.46666667
chr15:30882794-30885794	3000	0.33333333
chr15:31009794-31012794	3000	0.83333333
chr15:32064794-32067794	3000	0.6
chr15:32069794-32072794	3000	1.56666667
chr15:32076794-32080794	4000	0.875
chr15:33443794-33447794	4000	1
chr15:37308466-37311466	3000	1.2
chr15:37813466-37816466	3000	1
chr15:38012466-38015466	3000	1.6
chr15:38062466-38066466	4000	0.375
chr15:38180466-38183466	3000	0.6
chr15:38275466-38278466	3000	0.33333333
chr15:39093466-39096466	3000	0.9
chr15:40389466-40392466	3000	0.76666667
chr15:41184466-41188466	4000	0.625
chr15:41799466-41802466	3000	1
chr15:41886466-41890466	4000	0.575
chr15:48068786-48071786	3000	0.63333333
chr15:49003786-49006786	3000	0.63333333
chr15:51514107-51517107	3000	0.63333333
chr15:51680107-51683107	3000	0.76666667
chr15:51776107-51779107	3000	0.23333333
chr15:51842107-51846107	4000	0.925
chr15:51887107-51890107	3000	0.63333333
chr15:52557107-52562107	5000	0.42
chr15:52700107-52703107	3000	0.8

chr15:52796107-52799107	3000	0.46666667
chr15:52895107-52898107	3000	0.63333333
chr15:53090107-53093107	3000	0.9
chr15:53207107-53210107	3000	0.26666667
chr15:53642107-53645107	3000	0.66666667
chr15:53737107-53740107	3000	0.76666667
chr15:55034107-55037107	3000	0.5
chr15:55101107-55104107	3000	0.56666667
chr15:55150107-55154107	4000	0.6
chr15:58615107-58619107	4000	0.825
chr15:58683107-58686107	3000	1.03333333
chr15:58792107-58795107	3000	0.63333333
chr15:59302107-59307107	5000	1.02
chr15:59568107-59571107	3000	0.46666667
chr15:62275478-62278478	3000	0.6
chr15:62315478-62318478	3000	0.43333333
chr15:62325478-62328478	3000	0.6
chr15:62620478-62623478	3000	0.43333333
chr15:62692478-62696478	4000	0.325
chr15:62974478-62978478	4000	0.9
chr15:63755478-63759478	4000	0.4
chr15:63784478-63787478	3000	0.56666667
chr15:64004478-64007478	3000	0.4
chr15:64059478-64062478	3000	0.3
chr15:64069478-64073478	4000	0.525
chr15:64339478-64344478	5000	0.42
chr15:64589478-64593478	4000	0.25
chr15:65647478-65650478	3000	0.3
chr15:66005478-66009478	4000	0.575
chr15:68542478-68545478	3000	0.66666667
chr15:68552478-68555478	3000	0.33333333
chr15:68590478-68594478	4000	0.625
chr15:68623478-68626478	3000	1
chr15:68798478-68801478	3000	0.56666667
chr15:69012478-69015478	3000	0.36666667
chr15:69211478-69214478	3000	0.76666667
chr15:70638478-70641478	3000	0.43333333
chr15:70718478-70721478	3000	0.46666667
chr15:71244478-71247478	3000	0.5
chr15:72101478-72104478	3000	0.3
chr15:72293478-72296478	3000	0.66666667
chr15:72889478-72892478	3000	0.56666667
chr15:73676478-73680478	4000	0.225
chr15:73690478-73693478	3000	0.1
chr15:73806478-73810478	4000	0.575
chr15:74231478-74235478	4000	0.575
chr15:74513478-74516478	3000	0.23333333
chr15:74734478-74738478	4000	0.525
chr15:75252478-75256478	4000	0.3
chr15:75445478-75448478	3000	0.56666667
chr15:80747478-80752478	5000	0.4
chr15:80943478-80946478	3000	0.26666667
chr15:81716478-81719478	3000	1.4

chr15:84742478-84745478	3000	0.36666667
chr15:85024478-85027478	3000	0.76666667
chr15:86222478-86225478	3000	0.86666667
chr15:86524478-86527478	3000	0.8
chr15:86859478-86862478	3000	0.86666667
chr15:86963478-86967478	4000	0.75
chr15:87699478-87703478	4000	1.1
chr15:88070478-88073478	3000	1.36666667
chr15:88792478-88795478	3000	0.53333333
chr15:90225478-90229478	4000	0.525
chr15:90767478-90770478	3000	0.63333333
chr15:90994478-90997478	3000	0.86666667
chr15:91404478-91407478	3000	0.3
chr15:91442478-91446478	4000	0.675
chr15:92859478-92862478	3000	0.56666667
chr15:93384478-93388478	4000	0.6
chr15:93567478-93571478	4000	0.125
chr15:94184478-94187478	3000	1.03333333
chr15:94312478-94316478	4000	0.25
chr15:94539478-94544478	5000	0.36
chr15:94774478-94777478	3000	0.73333333
chr15:96118478-96121478	3000	0.5
chr15:97336478-97339478	3000	0.76666667
chr15:98243478-98246478	3000	0.6
chr15:98298478-98301478	3000	1.13333333
chr15:98866478-98870478	4000	0.95
chr15:99256478-99262478	6000	0.88333333
chr15:100079478-100083478	4000	0.675
chr15:100197478-100200478	3000	0.86666667
chr15:100713478-100720478	7000	0.8
chr15:101206478-101209478	3000	0.6
chr15:101359478-101362478	3000	0.56666667
chr15:101514478-101519478	5000	0.26
chr15:101680478-101684478	4000	1
chr15:102263478-102266478	3000	0.73333333
chr15:104991238-104994238	3000	0.53333333
chr15:105058238-105061238	3000	0.53333333
chr15:105077238-105080238	3000	0.73333333
chr15:105349238-105352238	3000	1.3
chr15:105805093-105808093	3000	1.53333333
chr15:108297927-108301927	4000	0.7
chr16:321001-325001	4000	0.525
chr16:3276001-3279001	3000	0.93333333
chr16:3897001-3900001	3000	2.4
chr16:4630001-4633001	3000	0.9
chr16:4654001-4657001	3000	1.36666667
chr16:4672001-4676001	4000	0.775
chr16:9387607-9390607	3000	1.43333333
chr16:9391607-9394607	3000	1.33333333
chr16:9465607-9469607	4000	1.025
chr16:9472607-9475607	3000	0.7
chr16:9841607-9845607	4000	1.05
chr16:11885607-11888607	3000	0.43333333

chr16:12251607-12255607	4000	0.35
chr16:13592644-13597644	5000	0.78
chr16:13902644-13905644	3000	0.66666667
chr16:14317644-14320644	3000	0.7
chr16:15574644-15577644	3000	0.5
chr16:15656644-15659644	3000	0.46666667
chr16:16706644-16710644	4000	0.45
chr16:16805644-16809644	4000	0.625
chr16:16882644-16887644	5000	0.38
chr16:17030644-17033644	3000	0.8
chr16:23692199-23695199	3000	0.7
chr16:24415199-24420199	5000	0.46
chr16:24978199-24981199	3000	0.5
chr16:25008199-25011199	3000	0.7
chr16:25792199-25796199	4000	0.425
chr16:26189199-26194199	5000	0.36
chr16:27270199-27273199	3000	1.23333333
chr16:27379199-27383199	4000	1.175
chr16:27556199-27560199	4000	0.8
chr16:28624199-28627199	3000	1.3
chr16:28854199-28858199	4000	0.175
chr16:29084199-29089199	5000	0.3
chr16:30310950-30313950	3000	0.83333333
chr16:30844950-30847950	3000	0.9
chr16:31449950-31453950	4000	1.175
chr16:31650950-31653950	3000	0.76666667
chr16:32673950-32676950	3000	0.43333333
chr16:32984950-32988950	4000	0.5
chr16:33010950-33015950	5000	0.3
chr16:33174950-33177950	3000	0.73333333
chr16:33296950-33299950	3000	0.33333333
chr16:38125950-38128950	3000	0.36666667
chr16:38724950-38727950	3000	0.33333333
chr16:38792950-38795950	3000	0.56666667
chr16:38805950-38808950	3000	0.53333333
chr16:39399950-39402950	3000	0.66666667
chr16:41229950-41232950	3000	1.23333333
chr16:41264950-41268950	4000	0.65
chr16:41456950-41459950	3000	0.63333333
chr16:41603950-41606950	3000	0.3
chr16:42013950-42018950	5000	0.54
chr16:42846950-42849950	3000	0.2
chr16:43162950-43165950	3000	0.4
chr16:43195950-43198950	3000	0.7
chr16:44495950-44498950	3000	0.4
chr16:45897950-45902950	5000	0.36
chr16:45989950-45992950	3000	0.46666667
chr16:49701950-49706950	5000	0.68
chr16:49977950-49980950	3000	0.43333333
chr16:49985950-49988950	3000	1.1
chr16:50312950-50315950	3000	1.1
chr16:50418950-50421950	3000	1.6
chr16:50822399-50825399	3000	0.8

chr16:51449399-51453399	4000	0.575
chr16:53296399-53299399	3000	0.36666667
chr16:53450399-53453399	3000	0.26666667
chr16:53469399-53473399	4000	0.65
chr16:53571399-53574399	3000	0.33333333
chr16:54199399-54204399	5000	1.04
chr16:54838399-54841399	3000	0.5
chr16:55770203-55773203	3000	0.9
chr16:56750203-56753203	3000	0.5
chr16:57170203-57174203	4000	0.425
chr16:57244203-57248203	4000	0.575
chr16:58278203-58282203	4000	0.35
chr16:58688203-58691203	3000	0.66666667
chr16:61378203-61382203	4000	1
chr16:61639203-61643203	4000	1.05
chr16:61648203-61651203	3000	0.76666667
chr16:63013203-63019203	6000	1.05
chr16:63244203-63248203	4000	0.7
chr16:63691203-63694203	3000	0.63333333
chr16:64413203-64416203	3000	0.43333333
chr16:64726203-64729203	3000	0.8
chr16:64853203-64856203	3000	1.3
chr16:65308604-65311604	3000	0.83333333
chr16:65407604-65410604	3000	0.93333333
chr16:65849604-65852604	3000	0.53333333
chr16:67467604-67470604	3000	0.53333333
chr16:69774732-69777732	3000	0.63333333
chr16:69858732-69861732	3000	1.26666667
chr16:70235732-70238732	3000	0.73333333
chr16:72211732-72214732	3000	1.23333333
chr16:72262732-72265732	3000	0.66666667
chr16:72724732-72727732	3000	0.96666667
chr16:72996732-73000732	4000	1.575
chr16:73201732-73205732	4000	1.05
chr16:76179732-76184732	5000	1.08
chr16:76533732-76536732	3000	0.46666667
chr16:76773732-76776732	3000	0.83333333
chr16:76888732-76892732	4000	0.725
chr16:77419732-77422732	3000	0.53333333
chr16:78177732-78180732	3000	0.7
chr16:78459732-78462732	3000	0.4
chr16:78733732-78738732	5000	0.62
chr16:78799732-78802732	3000	0.56666667
chr16:79406732-79409732	3000	1.06666667
chr16:79768732-79771732	3000	1.33333333
chr16:79772732-79777732	5000	0.96
chr16:79802732-79806732	4000	0.7
chr16:80093732-80097732	4000	0.55
chr16:80262732-80265732	3000	1.03333333
chr16:80412732-80415732	3000	1.26666667
chr16:80567732-80570732	3000	1.43333333
chr16:80974158-80978158	4000	0.8
chr16:82177158-82180158	3000	1.36666667

chr16:82233158-82238158	5000	0.8
chr16:82490158-82496158	6000	1.1
chr16:82554158-82558158	4000	1.325
chr16:83600319-83604319	4000	1.15
chr16:83719712-83722712	3000	1.36666667
chr16:83999712-84002712	3000	0.8
chr16:84101712-84104712	3000	0.83333333
chr16:84847712-84851712	4000	0.85
chr16:84979712-84982712	3000	0.86666667
chr16:85449712-85452712	3000	1.23333333
chr16:85589712-85592712	3000	1.03333333
chr16:85816712-85820712	4000	0.775
chr16:85827712-85831712	4000	1
chr16:85856712-85859712	3000	0.83333333
chr16:86500712-86503712	3000	1.36666667
chr16:86606712-86609712	3000	0.3
chr16:86777712-86783712	6000	0.78333333
chr16:88812712-88815712	3000	0.26666667
chr16:89023712-89026712	3000	0.43333333
chr16:89876712-89880712	4000	0.4
chr17:164001-167001	3000	0.6
chr17:264001-268001	4000	1.375
chr17:299001-302001	3000	0.8
chr17:407001-410001	3000	0.53333333
chr17:634001-638001	4000	0.55
chr17:853950-856950	3000	1.03333333
chr17:853950-856950	3000	1.03333333
chr17:903950-906950	3000	0.46666667
chr17:950950-953950	3000	1.13333333
chr17:1001950-1004950	3000	0.83333333
chr17:1056950-1061950	5000	0.54
chr17:1151950-1156950	5000	0.5
chr17:1256950-1260950	4000	0.4
chr17:1333950-1337950	4000	0.65
chr17:1483950-1486950	3000	0.6
chr17:1869950-1873950	4000	0.5
chr17:2076950-2079950	3000	0.33333333
chr17:2076950-2079950	3000	0.33333333
chr17:2510950-2516950	6000	0.51666667
chr17:2830950-2833950	3000	0.56666667
chr17:2846950-2853950	7000	1.05714286
chr17:2992950-2995950	3000	0.83333333
chr17:3034950-3037950	3000	0.63333333
chr17:3054950-3057950	3000	1.26666667
chr17:3069950-3072950	3000	0.4
chr17:3102950-3105950	3000	1.03333333
chr17:3444950-3447950	3000	0.36666667
chr17:3444950-3447950	3000	0.36666667
chr17:3490950-3495950	5000	0.68
chr17:3682950-3686950	4000	1.2
chr17:3694950-3699950	5000	1
chr17:3694950-3699950	5000	1
chr17:3721950-3724950	3000	0.9

chr17:4052950-4055950	3000	0.2
chr17:4057950-4060950	3000	0.76666667
chr17:4062950-4065950	3000	0.5
chr17:4062950-4065950	3000	0.5
chr17:4270950-4273950	3000	0.63333333
chr17:5028950-5031950	3000	0.76666667
chr17:5247950-5250950	3000	0.26666667
chr17:5247950-5250950	3000	0.26666667
chr17:5287950-5290950	3000	0.5
chr17:5559950-5563950	4000	0.425
chr17:10449950-10452950	3000	1.23333333
chr17:10696950-10699950	3000	1.6
chr17:11402950-11405950	3000	1.93333333
chr17:11787950-11790950	3000	1.36666667
chr17:11787950-11790950	3000	1.36666667
chr17:12835950-12838950	3000	1.73333333
chr17:12884950-12887950	3000	1.33333333
chr17:12905950-12909950	4000	0.975
chr17:13113950-13116950	3000	1.7
chr17:13162950-13165950	3000	1.46666667
chr17:13310950-13314950	4000	0.675
chr17:13354950-13358950	4000	0.875
chr17:13546950-13552950	6000	1.51666667
chr17:14281950-14284950	3000	1.6
chr17:16465777-16468777	3000	1.36666667
chr17:16745777-16749777	4000	1.525
chr17:16839777-16842777	3000	1.46666667
chr17:16839777-16842777	3000	1.46666667
chr17:16859777-16862777	3000	1.4
chr17:16859777-16862777	3000	1.4
chr17:16892777-16895777	3000	1.5
chr17:16892777-16895777	3000	1.5
chr17:16900777-16904777	4000	1.675
chr17:16984777-16988777	4000	1.775
chr17:16984777-16988777	4000	1.775
chr17:17569777-17573777	4000	0.775
chr17:18612777-18615777	3000	0.8
chr17:22340641-22344641	4000	1.975
chr17:22340641-22344641	4000	1.975
chr17:22729861-22732861	3000	1.63333333
chr17:22833861-22836861	3000	1.1
chr17:23183861-23189861	6000	0.6
chr17:24179861-24183861	4000	0.825
chr17:26455861-26458861	3000	1.06666667
chr17:26455861-26458861	3000	1.06666667
chr17:26631861-26636861	5000	1.08
chr17:27962861-27965861	3000	1.06666667
chr17:28231861-28236861	5000	0.42
chr17:28384775-28387775	3000	0.73333333
chr17:28384775-28387775	3000	0.73333333
chr17:28779775-28783775	4000	1
chr17:28984775-28989775	5000	0.92
chr17:28984775-28989775	5000	0.92

chr17:29060775-29063775	3000	1
chr17:29060775-29063775	3000	1
chr17:29270775-29274775	4000	0.55
chr17:29461775-29465775	4000	1.55
chr17:29489775-29492775	3000	2.73333333
chr17:30503775-30507775	4000	0.575
chr17:30540775-30543775	3000	1.03333333
chr17:31123775-31126775	3000	0.36666667
chr17:31127775-31130775	3000	1.2
chr17:31302775-31306775	4000	1
chr17:31638775-31641775	3000	0.56666667
chr17:31666775-31669775	3000	0.76666667
chr17:31754775-31757775	3000	1
chr17:31781775-31784775	3000	0.63333333
chr17:31938775-31942775	4000	1.125
chr17:31971775-31974775	3000	1.03333333
chr17:32008775-32011775	3000	0.8
chr17:33721775-33724775	3000	1.06666667
chr17:33923775-33928775	5000	0.48
chr17:33929775-33933775	4000	0.725
chr17:34026775-34029775	3000	0.9
chr17:34243775-34246775	3000	0.76666667
chr17:34368775-34371775	3000	0.8
chr17:34483775-34488775	5000	0.42
chr17:34516775-34520775	4000	1.025
chr17:34535775-34538775	3000	1.2
chr17:34535775-34538775	3000	1.2
chr17:35130775-35133775	3000	0.8
chr17:35742775-35745775	3000	0.3
chr17:35742775-35745775	3000	0.3
chr17:35803775-35807775	4000	0.95
chr17:35863775-35866775	3000	0.26666667
chr17:35925775-35929775	4000	0.55
chr17:36014775-36018775	4000	0.65
chr17:36069775-36073775	4000	0.625
chr17:36191775-36194775	3000	1.03333333
chr17:36205775-36208775	3000	1.23333333
chr17:36205775-36208775	3000	1.23333333
chr17:36463775-36466775	3000	0.46666667
chr17:36468775-36472775	4000	0.875
chr17:36537775-36540775	3000	0.83333333
chr17:36605775-36609775	4000	1.225
chr17:36605775-36609775	4000	1.225
chr17:36707775-36711775	4000	0.7
chr17:36759775-36762775	3000	0.56666667
chr17:37603176-37606176	3000	0.26666667
chr17:37732176-37735176	3000	0.63333333
chr17:37740176-37743176	3000	0.63333333
chr17:37740176-37743176	3000	0.63333333
chr17:37782176-37785176	3000	0.7
chr17:37805176-37808176	3000	0.53333333
chr17:37827176-37830176	3000	0.63333333
chr17:37863176-37869176	6000	0.56666667

chr17:38169176-38174176	5000	0.68
chr17:38169176-38174176	5000	0.68
chr17:38189176-38193176	4000	0.975
chr17:38407176-38412176	5000	0.5
chr17:38407176-38412176	5000	0.5
chr17:39344176-39347176	3000	1.03333333
chr17:39344176-39347176	3000	1.03333333
chr17:39609176-39612176	3000	0.9
chr17:39668176-39672176	4000	0.6
chr17:39686176-39689176	3000	1
chr17:39870176-39873176	3000	0.6
chr17:40344176-40347176	3000	0.76666667
chr17:40593176-40598176	5000	0.64
chr17:40754176-40757176	3000	0.43333333
chr17:43645176-43648176	3000	0.46666667
chr17:43771176-43774176	3000	0.6
chr17:44176176-44180176	4000	0.425
chr17:45392176-45395176	3000	0.56666667
chr17:45621176-45626176	5000	0.46
chr17:46469176-46472176	3000	0.23333333
chr17:46548176-46551176	3000	0.2
chr17:46847176-46850176	3000	0.36666667
chr17:46853176-46856176	3000	0.43333333
chr17:46858176-46861176	3000	1.83333333
chr17:51124473-51127473	3000	1.2
chr17:51869473-51872473	3000	0.26666667
chr17:51878473-51882473	4000	0.5
chr17:51883473-51886473	3000	0.26666667
chr17:52106473-52109473	3000	0.36666667
chr17:53164473-53167473	3000	0.53333333
chr17:53351473-53354473	3000	0.66666667
chr17:54029473-54032473	3000	0.66666667
chr17:54468473-54472473	4000	0.35
chr17:54783473-54788473	5000	0.78
chr17:55047473-55050473	3000	0.26666667
chr17:55114473-55117473	3000	0.53333333
chr17:55664473-55668473	4000	0.475
chr17:56680473-56683473	3000	0.8
chr17:56949473-56952473	3000	0.33333333
chr17:57124473-57127473	3000	0.4
chr17:57375473-57378473	3000	0.4
chr17:57486473-57489473	3000	0.33333333
chr17:57919473-57922473	3000	0.53333333
chr17:58485473-58488473	3000	0.3
chr17:58944473-58947473	3000	0.66666667
chr17:62020079-62023079	3000	1.16666667
chr17:62989079-62992079	3000	0.63333333
chr17:62993079-62996079	3000	0.73333333
chr17:63002079-63006079	4000	0.5
chr17:63375079-63378079	3000	0.63333333
chr17:63389079-63392079	3000	0.96666667
chr17:63441079-63444079	3000	1.5
chr17:63951079-63955079	4000	1.275

chr17:65045079-65048079	3000	0.83333333
chr17:66076079-66079079	3000	0.7
chr17:66191079-66195079	4000	0.575
chr17:70033896-70036896	3000	0.5
chr17:73437616-73441616	4000	0.55
chr17:73611616-73614616	3000	0.93333333
chr17:74164616-74167616	3000	0.76666667
chr17:75210616-75215616	5000	0.56
chr17:76371616-76377616	6000	0.63333333
chr17:76395616-76398616	3000	0.73333333
chr17:76699616-76702616	3000	0.3
chr17:77150616-77153616	3000	0.63333333
chr17:80876616-80879616	3000	0.33333333
chr17:81647616-81650616	3000	0.23333333
chr17:81978616-81981616	3000	0.66666667
chr17:82129616-82133616	4000	1.075
chr17:89627279-89630279	3000	0.56666667
chr17:89821279-89824279	3000	1.06666667
chr17:91032279-91037279	5000	0.46
chr17:91128279-91133279	5000	0.38
chr17:91339279-91342279	3000	0.93333333
chr17:91368279-91371279	3000	0.56666667
chr17:95829279-95832279	3000	0.6
chr17:95978279-95981279	3000	0.36666667
chr17:96055279-96058279	3000	1.03333333
chr18:929001-932001	3000	0.23333333
chr18:2794263-2798263	4000	0.425
chr18:5198485-5202485	4000	1.025
chr18:5683485-5687485	4000	0.875
chr18:7017485-7023485	6000	0.53333333
chr18:7199485-7203485	4000	0.85
chr18:8442485-8446485	4000	0.45
chr18:8873485-8876485	3000	0.2
chr18:8950485-8954485	4000	0.525
chr18:9511485-9514485	3000	0.36666667
chr18:9966485-9969485	3000	0.43333333
chr18:11197485-11200485	3000	0.33333333
chr18:13727841-13730841	3000	1
chr18:17804841-17808841	4000	0.5
chr18:18285841-18288841	3000	0.63333333
chr18:18353841-18357841	4000	0.25
chr18:18907841-18910841	3000	0.56666667
chr18:19512841-19515841	3000	0.43333333
chr18:19877841-19880841	3000	0.43333333
chr18:20175841-20179841	4000	0.425
chr18:21319841-21322841	3000	0.7
chr18:21610841-21614841	4000	0.85
chr18:22718841-22721841	3000	0.66666667
chr18:23568841-23571841	3000	0.6
chr18:23775841-23778841	3000	0.56666667
chr18:23983841-23986841	3000	0.6
chr18:25254841-25258841	4000	0.65
chr18:26216841-26219841	3000	1.06666667

chr18:29837571-29840571	3000	0.56666667
chr18:30443571-30446571	3000	0.6
chr18:30497571-30502571	5000	0.28
chr18:33200986-33204986	4000	0.875
chr18:33484986-33488986	4000	0.4
chr18:34179986-34183986	4000	0.225
chr18:34197986-34200986	3000	0.43333333
chr18:34487986-34490986	3000	0.5
chr18:36357986-36360986	3000	0.33333333
chr18:36644986-36647986	3000	1
chr18:36803986-36806986	3000	0.7
chr18:37081986-37084986	3000	0.5
chr18:39445986-39449986	4000	0.35
chr18:39731961-39735961	4000	0.75
chr18:40300961-40304961	4000	0.95
chr18:40318961-40322961	4000	0.575
chr18:41085961-41089961	4000	1.5
chr18:41125961-41128961	3000	0.46666667
chr18:43102961-43105961	3000	0.4
chr18:43683961-43686961	3000	0.5
chr18:44261961-44264961	3000	0.43333333
chr18:44294961-44297961	3000	0.4
chr18:44420961-44424961	4000	0.55
chr18:44631961-44636961	5000	0.58
chr18:46720451-46724451	4000	0.45
chr18:47153451-47156451	3000	0.36666667
chr18:47633451-47636451	3000	0.6
chr18:47818451-47821451	3000	0.96666667
chr18:48033451-48037451	4000	0.65
chr18:48115451-48119451	4000	1.25
chr18:51005451-51010451	5000	0.78
chr18:51947451-51953451	6000	0.45
chr18:53264451-53270451	6000	0.65
chr18:53571451-53574451	3000	1.4
chr18:54077451-54084451	7000	0.87142857
chr18:54895451-54898451	3000	1.06666667
chr18:55010451-55013451	3000	0.5
chr18:55082451-55087451	5000	0.42
chr18:55718451-55722451	4000	0.45
chr18:55928451-55931451	3000	0.86666667
chr18:56023451-56027451	4000	0.575
chr18:56248451-56251451	3000	1
chr18:56344451-56347451	3000	0.3
chr18:58783159-58788159	5000	0.46
chr18:58907159-58910159	3000	1.2
chr18:59683159-59686159	3000	1.4
chr18:59709159-59712159	3000	0.7
chr18:63189418-63192418	3000	0.96666667
chr18:64674418-64677418	3000	1.1
chr18:64707418-64710418	3000	0.8
chr18:64718418-64722418	4000	0.95
chr18:64949418-64953418	4000	1.1
chr18:65352418-65356418	4000	1.15

chr18:67475418-67478418	3000	0.4
chr18:68202418-68206418	4000	0.6
chr18:70114418-70117418	3000	1.43333333
chr18:70263418-70267418	4000	1.775
chr18:70281418-70284418	3000	0.56666667
chr18:70329418-70332418	3000	1.2
chr18:71108418-71111418	3000	0.8
chr18:71205418-71208418	3000	0.33333333
chr18:72581418-72584418	3000	0.76666667
chr18:73578418-73581418	3000	1.03333333
chr18:75578418-75581418	3000	0.63333333
chr18:75727418-75731418	4000	1.15
chr18:76230418-76233418	3000	0.73333333
chr18:76861418-76864418	3000	0.5
chr18:77284418-77287418	3000	1.5
chr18:78190418-78193418	3000	0.86666667
chr18:78391418-78394418	3000	0.66666667
chr18:78401418-78404418	3000	1.23333333
chr18:78576418-78581418	5000	1.08
chr18:80233418-80236418	3000	0.6
chr18:80469418-80472418	3000	1.33333333
chr18:80658418-80661418	3000	1.56666667
chr18:80937418-80940418	3000	0.7
chr18:80949418-80952418	3000	1.46666667
chr18:81147418-81150418	3000	0.6
chr18:81733418-81736418	3000	0.43333333
chr18:81804418-81807418	3000	0.56666667
chr18:82141418-82145418	4000	0.7
chr18:82671418-82674418	3000	0.7
chr18:82706418-82709418	3000	0.73333333
chr18:82880418-82886418	6000	0.36666667
chr18:82933418-82936418	3000	0.3
chr18:84393418-84398418	5000	0.26
chr18:85172418-85175418	3000	0.33333333
chr18:85183418-85188418	5000	0.6
chr18:85362418-85365418	3000	0.46666667
chr18:85589418-85592418	3000	0.66666667
chr18:86208418-86211418	3000	0.43333333
chr18:87055418-87058418	3000	1.03333333
chr19:1675004-1678004	3000	0.4
chr19:1794004-1797004	3000	0.5
chr19:1809004-1812004	3000	0.36666667
chr19:3626004-3632004	6000	0.66666667
chr19:3726004-3730004	4000	0.45
chr19:4377004-4380004	3000	0.46666667
chr19:5119004-5123004	4000	0.65
chr19:6206861-6209861	3000	0.53333333
chr19:6804861-6807861	3000	0.66666667
chr19:6899861-6903861	4000	0.6
chr19:7962861-7965861	3000	0.46666667
chr19:8063861-8066861	3000	0.96666667
chr19:8429861-8432861	3000	0.56666667
chr19:8674861-8678861	4000	0.775

chr19:8723861-8726861	3000	0.2
chr19:9147861-9150861	3000	1.06666667
chr19:12037585-12041585	4000	0.725
chr19:12547585-12550585	3000	1.03333333
chr19:12594585-12597585	3000	0.63333333
chr19:12703585-12706585	3000	0.83333333
chr19:13102585-13105585	3000	0.63333333
chr19:13167585-13170585	3000	0.9
chr19:13186585-13189585	3000	1.3
chr19:13228585-13232585	4000	0.525
chr19:17852414-17857414	5000	1.04
chr19:18021414-18024414	3000	0.46666667
chr19:18236414-18239414	3000	0.43333333
chr19:18651414-18655414	4000	0.725
chr19:18660414-18663414	3000	1.33333333
chr19:18843414-18846414	3000	1.1
chr19:19141414-19144414	3000	0.83333333
chr19:19638414-19641414	3000	0.43333333
chr19:21106563-21110563	4000	0.9
chr19:21309563-21313563	4000	0.95
chr19:21382563-21386563	4000	0.7
chr19:26399352-26402352	3000	1.16666667
chr19:28220352-28223352	3000	0.53333333
chr19:28366352-28369352	3000	0.73333333
chr19:28631352-28634352	3000	0.86666667
chr19:31673352-31676352	3000	1
chr19:32032352-32037352	5000	0.52
chr19:32888352-32891352	3000	0.36666667
chr19:32940352-32944352	4000	0.525
chr19:33118352-33121352	3000	0.23333333
chr19:33197352-33200352	3000	0.56666667
chr19:33412352-33416352	4000	0.4
chr19:33674352-33678352	4000	0.475
chr19:34118352-34121352	3000	0.56666667
chr19:34702352-34706352	4000	0.35
chr19:48099781-48104781	5000	1.28
chr19:48182781-48185781	3000	2.16666667
chr19:48198781-48201781	3000	1.03333333
chr19:56157507-56160507	3000	1.63333333
chr2:166001-169001	3000	0.3
chr2:1180001-1183001	3000	0.43333333
chr2:1242001-1245001	3000	0.46666667
chr2:2047001-2050001	3000	0.2
chr2:3337001-3340001	3000	0.53333333
chr2:6292001-6295001	3000	0.56666667
chr2:7179001-7182001	3000	0.43333333
chr2:7412001-7415001	3000	0.3
chr2:7489001-7494001	5000	0.42
chr2:7626001-7630001	4000	0.7
chr2:7809001-7812001	3000	0.4
chr2:8442001-8445001	3000	0.6
chr2:8789001-8792001	3000	0.7
chr2:9884001-9888001	4000	1.05

chr2:10378001-10381001	3000	1.76666667
chr2:10531001-10536001	5000	0.46
chr2:11276001-11279001	3000	0.5
chr2:12805001-12809001	4000	0.725
chr2:14403001-14406001	3000	0.1
chr2:15217001-15221001	4000	0.425
chr2:15492001-15495001	3000	0.53333333
chr2:18909001-18912001	3000	0.33333333
chr2:19666001-19669001	3000	0.43333333
chr2:19687001-19694001	7000	0.5
chr2:20848001-20854001	6000	0.46666667
chr2:21513001-21516001	3000	1.06666667
chr2:22403001-22407001	4000	0.525
chr2:23312001-23315001	3000	0.73333333
chr2:25489001-25492001	3000	0.7
chr2:27997019-28000019	3000	1.13333333
chr2:28116019-28119019	3000	1.46666667
chr2:30654019-30657019	3000	1.36666667
chr2:30686019-30689019	3000	1.03333333
chr2:32484655-32487655	3000	1.3
chr2:32558655-32561655	3000	0.4
chr2:32951655-32954655	3000	0.56666667
chr2:32997655-33000655	3000	0.86666667
chr2:33395655-33398655	3000	1.13333333
chr2:37769655-37772655	3000	0.3
chr2:39927655-39931655	4000	0.75
chr2:39936655-39939655	3000	0.83333333
chr2:39975655-39978655	3000	0.46666667
chr2:40782655-40785655	3000	0.33333333
chr2:41604655-41608655	4000	0.325
chr2:42317655-42320655	3000	0.76666667
chr2:42533655-42537655	4000	0.675
chr2:44970655-44978655	8000	1.125
chr2:45352655-45355655	3000	0.86666667
chr2:46112655-46115655	3000	0.3
chr2:46782655-46785655	3000	0.36666667
chr2:46806655-46809655	3000	0.6
chr2:46857655-46861655	4000	0.725
chr2:47043655-47046655	3000	0.36666667
chr2:47299655-47302655	3000	0.43333333
chr2:48714655-48717655	3000	0.66666667
chr2:49612655-49615655	3000	0.6
chr2:49733655-49736655	3000	0.7
chr2:50011655-50014655	3000	0.56666667
chr2:50136655-50139655	3000	0.46666667
chr2:50291655-50294655	3000	0.53333333
chr2:50420655-50424655	4000	1.125
chr2:51182655-51185655	3000	0.63333333
chr2:51299655-51303655	4000	0.6
chr2:51445655-51448655	3000	0.66666667
chr2:53094655-53099655	5000	0.42
chr2:53920655-53924655	4000	0.8
chr2:53926655-53930655	4000	1.1

chr2:55535655-55538655	3000	1.26666667
chr2:56178655-56181655	3000	0.53333333
chr2:56198655-56201655	3000	0.63333333
chr2:57053659-57057659	4000	0.375
chr2:60253724-60256724	3000	0.26666667
chr2:60317724-60320724	3000	0.56666667
chr2:60508724-60511724	3000	0.46666667
chr2:61109724-61113724	4000	0.95
chr2:62949724-62952724	3000	0.83333333
chr2:62963724-62968724	5000	0.78
chr2:63761724-63765724	4000	0.175
chr2:64150724-64154724	4000	0.2
chr2:65517724-65520724	3000	0.23333333
chr2:65521724-65524724	3000	0.36666667
chr2:66953724-66957724	4000	0.475
chr2:67811724-67814724	3000	0.63333333
chr2:68806724-68809724	3000	0.26666667
chr2:68961724-68964724	3000	0.4
chr2:69343724-69346724	3000	0.43333333
chr2:69743724-69746724	3000	0.33333333
chr2:69913724-69916724	3000	0.56666667
chr2:70206724-70210724	4000	0.375
chr2:70524724-70527724	3000	1.26666667
chr2:70804724-70807724	3000	0.3
chr2:72206724-72209724	3000	0.43333333
chr2:72299724-72302724	3000	0.66666667
chr2:73073724-73076724	3000	0.53333333
chr2:73309724-73312724	3000	0.56666667
chr2:73539724-73547724	8000	0.35
chr2:73593724-73596724	3000	0.6
chr2:73666724-73670724	4000	0.475
chr2:74035724-74038724	3000	0.43333333
chr2:74292724-74295724	3000	0.7
chr2:75346724-75349724	3000	0.83333333
chr2:75784724-75787724	3000	0.56666667
chr2:75946724-75949724	3000	1
chr2:77710724-77713724	3000	0.3
chr2:77731724-77734724	3000	0.26666667
chr2:77748724-77751724	3000	0.7
chr2:77823724-77826724	3000	0.5
chr2:77885724-77888724	3000	0.3
chr2:78114724-78117724	3000	0.46666667
chr2:78282724-78285724	3000	0.4
chr2:78384724-78387724	3000	0.66666667
chr2:78777724-78781724	4000	0.4
chr2:79022724-79027724	5000	0.38
chr2:79096724-79100724	4000	0.675
chr2:80808724-80812724	4000	0.25
chr2:81204724-81208724	4000	0.55
chr2:81836724-81840724	4000	0.4
chr2:82770724-82773724	3000	0.6
chr2:82888724-82891724	3000	0.5
chr2:83133724-83136724	3000	0.46666667

chr2:83801724-83804724	3000	0.4
chr2:83833724-83836724	3000	1.13333333
chr2:83879724-83882724	3000	0.66666667
chr2:84005724-84008724	3000	0.56666667
chr2:84065724-84069724	4000	0.5
chr2:84648724-84651724	3000	0.53333333
chr2:85539724-85542724	3000	0.26666667
chr2:85736724-85739724	3000	0.26666667
chr2:86113724-86117724	4000	0.25
chr2:86159724-86162724	3000	0.56666667
chr2:86460724-86464724	4000	0.375
chr2:86574724-86577724	3000	0.2
chr2:87479724-87482724	3000	0.53333333
chr2:90145724-90150724	5000	0.56
chr2:90188724-90191724	3000	0.33333333
chr2:90485724-90489724	4000	0.25
chr2:92329724-92332724	3000	0.4
chr2:93878724-93881724	3000	2.63333333
chr2:95030724-95034724	4000	1.325
chr2:95671724-95675724	4000	0.4
chr2:96436724-96440724	4000	0.85
chr2:96754724-96757724	3000	0.2
chr2:97510724-97513724	3000	0.13333333
chr2:99154724-99157724	3000	0.63333333
chr2:100284724-100288724	4000	0.3
chr2:100957724-100960724	3000	0.43333333
chr2:101518724-101521724	3000	0.5
chr2:102303724-102306724	3000	0.36666667
chr2:103299724-103303724	4000	0.375
chr2:103573724-103577724	4000	0.1
chr2:103965724-103969724	4000	0.325
chr2:103996724-103999724	3000	0.43333333
chr2:104613724-104616724	3000	1.06666667
chr2:104689724-104692724	3000	0.53333333
chr2:105074724-105082724	8000	0.6
chr2:105606724-105609724	3000	0.86666667
chr2:105865724-105868724	3000	0.43333333
chr2:105969724-105972724	3000	0.36666667
chr2:106044724-106048724	4000	0.3
chr2:106183724-106187724	4000	0.475
chr2:106680724-106684724	4000	0.35
chr2:106882724-106886724	4000	0.725
chr2:107109724-107115724	6000	0.53333333
chr2:108168724-108171724	3000	0.36666667
chr2:108201724-108204724	3000	0.4
chr2:108320724-108325724	5000	0.28
chr2:108818724-108821724	3000	0.43333333
chr2:108981724-108986724	5000	0.6
chr2:109167724-109170724	3000	0.36666667
chr2:109404724-109408724	4000	0.275
chr2:109504724-109508724	4000	0.425
chr2:109769724-109772724	3000	0.43333333
chr2:109788724-109791724	3000	0.66666667

chr2:111126724-111129724	3000	0.43333333
chr2:111132724-111135724	3000	0.26666667
chr2:111265724-111269724	4000	0.425
chr2:112281724-112284724	3000	1.26666667
chr2:112554724-112557724	3000	1.03333333
chr2:114348724-114352724	4000	0.3
chr2:115179724-115182724	3000	1.03333333
chr2:118032112-118035112	3000	0.6
chr2:118246112-118249112	3000	1.03333333
chr2:119570541-119573541	3000	0.46666667
chr2:119720541-119723541	3000	0.2
chr2:122836541-122840541	4000	0.675
chr2:122973541-122976541	3000	1
chr2:122981541-122984541	3000	0.9
chr2:125347541-125350541	3000	0.56666667
chr2:125653541-125656541	3000	1.16666667
chr2:126050541-126053541	3000	0.96666667
chr2:126227541-126231541	4000	0.45
chr2:126339541-126342541	3000	0.73333333
chr2:127281541-127284541	3000	0.26666667
chr2:129445541-129448541	3000	0.5
chr2:129925541-129930541	5000	0.34
chr2:131820541-131823541	3000	0.6
chr2:132172541-132175541	3000	0.5
chr2:137430541-137433541	3000	0.4
chr2:138411541-138414541	3000	0.53333333
chr2:139029541-139033541	4000	0.525
chr2:139340541-139345541	5000	0.42
chr2:139574541-139579541	5000	0.38
chr2:141636541-141639541	3000	1.26666667
chr2:141740541-141743541	3000	1.06666667
chr2:142222541-142226541	4000	0.975
chr2:143821541-143824541	3000	1.13333333
chr2:144290541-144293541	3000	0.93333333
chr2:145425541-145428541	3000	0.43333333
chr2:145596541-145599541	3000	0.7
chr2:145744541-145749541	5000	0.62
chr2:146033541-146036541	3000	0.7
chr2:146069541-146072541	3000	0.43333333
chr2:147270541-147273541	3000	0.73333333
chr2:147487541-147490541	3000	1.13333333
chr2:147523541-147530541	7000	0.62857143
chr2:148187541-148191541	4000	0.65
chr2:149072110-149075110	3000	0.43333333
chr2:149276110-149279110	3000	0.4
chr2:149691110-149694110	3000	0.63333333
chr2:149695110-149698110	3000	0.46666667
chr2:150105110-150108110	3000	1.23333333
chr2:150290110-150294110	4000	0.5
chr2:150402110-150405110	3000	0.36666667
chr2:150432110-150435110	3000	0.8
chr2:150618110-150621110	3000	0.4
chr2:150674110-150677110	3000	1.16666667

chr2:150722110-150727110	5000	0.5
chr2:151051110-151054110	3000	0.4
chr2:151365110-151368110	3000	0.36666667
chr2:151955110-151960110	5000	0.42
chr2:152654110-152657110	3000	0.53333333
chr2:152856110-152859110	3000	0.36666667
chr2:153307110-153310110	3000	0.7
chr2:153395110-153398110	3000	0.6
chr2:153418110-153422110	4000	0.575
chr2:153533110-153536110	3000	1.1
chr2:154848110-154851110	3000	0.9
chr2:156219110-156222110	3000	1.6
chr2:156248110-156251110	3000	0.6
chr2:156524110-156529110	5000	0.76
chr2:156635110-156638110	3000	0.33333333
chr2:157244110-157249110	5000	0.48
chr2:157287110-157290110	3000	1.73333333
chr2:157629110-157633110	4000	0.575
chr2:157979110-157982110	3000	0.33333333
chr2:159826110-159831110	5000	1.06
chr2:160096110-160100110	4000	0.5
chr2:160179110-160182110	3000	0.43333333
chr2:160394110-160397110	3000	0.53333333
chr2:160613110-160616110	3000	0.46666667
chr2:160803110-160806110	3000	0.63333333
chr2:161329110-161332110	3000	0.26666667
chr2:161844110-161847110	3000	0.66666667
chr2:162159110-162162110	3000	0.16666667
chr2:162182110-162188110	6000	0.43333333
chr2:162950110-162953110	3000	0.36666667
chr2:163091110-163094110	3000	0.53333333
chr2:163156110-163161110	5000	0.56
chr2:164604110-164609110	5000	0.12
chr2:164822110-164825110	3000	0.33333333
chr2:165445110-165449110	4000	0.5
chr2:165495110-165498110	3000	0.5
chr2:165559110-165564110	5000	0.36
chr2:166211110-166214110	3000	0.33333333
chr2:168150110-168155110	5000	0.64
chr2:168901110-168904110	3000	0.2
chr2:169597110-169603110	6000	0.45
chr2:170403110-170409110	6000	0.85
chr2:171792110-171795110	3000	0.83333333
chr2:171885110-171888110	3000	0.2
chr2:172136110-172139110	3000	0.43333333
chr2:172334110-172338110	4000	0.725
chr2:173198110-173201110	3000	0.53333333
chr2:173892110-173896110	4000	0.825
chr2:174137110-174140110	3000	0.23333333
chr2:174588110-174592110	4000	0.625
chr2:174856110-174861110	5000	0.88
chr2:175050110-175053110	3000	0.43333333
chr2:175239110-175242110	3000	0.86666667

chr2:175466110-175471110	5000	1.14
chr2:175604319-175607319	3000	0.83333333
chr2:175636319-175640319	4000	0.625
chr2:176951319-176955319	4000	0.3
chr2:177170319-177173319	3000	0.86666667
chr2:177381319-177384319	3000	0.96666667
chr2:177681319-177685319	4000	0.975
chr2:185021893-185024893	3000	0.86666667
chr2:187134893-187137893	3000	0.66666667
chr2:187782893-187785893	3000	0.76666667
chr2:188566893-188570893	4000	0.575
chr2:188760893-188763893	3000	0.8
chr2:188779893-188782893	3000	1
chr2:189075893-189079893	4000	0.925
chr2:193522286-193525286	3000	0.53333333
chr2:194123286-194126286	3000	0.36666667
chr2:194209286-194213286	4000	0.4
chr2:195071286-195074286	3000	0.5
chr2:195278286-195281286	3000	0.53333333
chr2:195342286-195346286	4000	0.6
chr2:195373286-195376286	3000	0.76666667
chr2:197508286-197514286	6000	0.51666667
chr2:197659286-197662286	3000	0.6
chr2:201629536-201632536	3000	0.36666667
chr2:201670536-201673536	3000	0.56666667
chr2:202046536-202049536	3000	0.53333333
chr2:202068536-202071536	3000	0.4
chr2:202237536-202240536	3000	0.43333333
chr2:202581536-202586536	5000	0.7
chr2:205337186-205340186	3000	0.56666667
chr2:205407186-205414186	7000	0.52857143
chr2:205733186-205736186	3000	1
chr2:206013249-206017249	4000	0.5
chr2:206599249-206602249	3000	0.5
chr2:207056249-207060249	4000	0.225
chr2:207081249-207086249	5000	0.34
chr2:207258249-207261249	3000	0.76666667
chr2:207329249-207333249	4000	0.775
chr2:211269249-211272249	3000	0.53333333
chr2:211353249-211356249	3000	0.63333333
chr2:211447249-211450249	3000	0.46666667
chr2:211624249-211627249	3000	0.66666667
chr2:212480249-212483249	3000	0.6
chr2:213374249-213378249	4000	0.375
chr2:214337249-214340249	3000	0.6
chr2:214450249-214453249	3000	0.76666667
chr2:214748249-214752249	4000	0.925
chr2:216889249-216893249	4000	0.65
chr2:220630205-220633205	3000	0.53333333
chr2:220661205-220664205	3000	0.76666667
chr2:221758205-221763205	5000	0.34
chr2:223721205-223725205	4000	0.875
chr2:224142205-224146205	4000	0.45

chr2:225632205-225635205	3000	0.96666667
chr2:225733205-225736205	3000	0.86666667
chr2:226799205-226802205	3000	0.76666667
chr2:227454205-227457205	3000	0.83333333
chr2:228081205-228084205	3000	0.66666667
chr2:229508205-229512205	4000	1.35
chr2:229932202-229936202	4000	1.2
chr2:231526202-231529202	3000	0.53333333
chr2:231635202-231638202	3000	0.46666667
chr2:231839202-231842202	3000	0.73333333
chr2:232165202-232168202	3000	0.86666667
chr2:232685202-232688202	3000	0.9
chr2:234104202-234107202	3000	0.76666667
chr2:234430202-234433202	3000	0.46666667
chr2:234801202-234804202	3000	0.66666667
chr2:235600202-235603202	3000	1.83333333
chr2:236923202-236926202	3000	1.16666667
chr2:237229202-237232202	3000	0.7
chr2:238180202-238183202	3000	0.26666667
chr2:239901202-239904202	3000	0.7
chr2:242133202-242138202	5000	0.5
chr2:243143202-243146202	3000	1.03333333
chr2:244536202-244539202	3000	2.13333333
chr2:245683202-245687202	4000	0.8
chr2:246021202-246024202	3000	0.4
chr2:246971202-246974202	3000	0.7
chr2:248488202-248491202	3000	0.63333333
chr2:249298202-249301202	3000	0.66666667
chr2:249446202-249451202	5000	0.96
chr2:249914202-249919202	5000	0.64
chr2:250051202-250055202	4000	0.675
chr2:251334202-251337202	3000	1.73333333
chr2:251390202-251393202	3000	1.2
chr2:252502112-252505112	3000	1.33333333
chr2:254415112-254418112	3000	0.53333333
chr2:255398112-255401112	3000	0.23333333
chr2:255517112-255520112	3000	0.93333333
chr2:255564112-255567112	3000	0.4
chr2:257689112-257692112	3000	0.5
chr20:1481929-1485929	4000	1.05
chr20:1662929-1666929	4000	0.775
chr20:2788508-2791508	3000	1.1
chr20:3144397-3153397	9000	1.54444444
chr20:3390397-3394397	4000	1.1
chr20:4087145-4090145	3000	2.7
chr20:5006671-5010671	4000	1.575
chr20:5274938-5277938	3000	2.4
chr20:10448938-10451938	3000	2.2
chr20:10782938-10786938	4000	1.225
chr20:10892938-10895938	3000	1.1
chr20:10903938-10907938	4000	1.05
chr20:11609044-11612044	3000	0.66666667
chr20:12617612-12630612	13000	1.91538462

chr20:12959612-12963612	4000	1.675
chr20:13111612-13115612	4000	2.7
chr20:13184612-13187612	3000	1.26666667
chr20:13529378-13536378	7000	1.38571429
chr20:13602378-13608378	6000	2
chr20:13878378-13881378	3000	1.03333333
chr20:25361900-25367900	6000	1.75
chr20:25530900-25533900	3000	2.13333333
chr20:25788900-25791900	3000	0.9
chr20:25852900-25855900	3000	1.1
chr20:26742900-26745900	3000	0.66666667
chr20:27188900-27197900	9000	1.4
chr20:27221900-27224900	3000	1.5
chr20:28268204-28271204	3000	0.53333333
chr20:28522204-28528204	6000	1.36666667
chr20:28723204-28732204	9000	1.61111111
chr20:28921204-28925204	4000	0.95
chr20:29404204-29407204	3000	1.06666667
chr20:29504204-29507204	3000	1.2
chr20:29721981-29729981	8000	0.9875
chr20:30229981-30232981	3000	1.06666667
chr20:45897981-45902981	5000	1.52
chr20:46106981-46110981	4000	2.075
chr20:46171981-46174981	3000	2
chr20:46690981-46695981	5000	1
chr20:47160981-47169981	9000	2.16666667
chr20:47564981-47567981	3000	1.26666667
chr20:47792981-47799981	7000	1.41428571
chr20:48048981-48051981	3000	1.26666667
chr20:48269981-48272981	3000	1.16666667
chr20:48319981-48326981	7000	1.6
chr20:48748981-48751981	3000	1.16666667
chr20:54869981-54872981	3000	0.53333333
chr20:54898981-54901981	3000	0.8
chr3:314001-317001	3000	0.56666667
chr3:597001-601001	4000	0.725
chr3:846001-849001	3000	0.96666667
chr3:1318001-1321001	3000	0.43333333
chr3:1671001-1674001	3000	0.4
chr3:2367001-2370001	3000	0.26666667
chr3:2582001-2585001	3000	0.63333333
chr3:2883001-2889001	6000	0.55
chr3:5436626-5439626	3000	0.3
chr3:6187626-6190626	3000	1.33333333
chr3:6738626-6741626	3000	0.66666667
chr3:6976626-6979626	3000	0.83333333
chr3:7292626-7295626	3000	1.3
chr3:7310626-7313626	3000	1.5
chr3:20103952-20106952	3000	0.3
chr3:20136952-20140952	4000	0.425
chr3:20280952-20283952	3000	0.36666667
chr3:20553952-20556952	3000	0.3
chr3:20822952-20826952	4000	0.375

chr3:21860952-21863952	3000	0.6
chr3:21900952-21904952	4000	0.325
chr3:22119952-22122952	3000	0.33333333
chr3:23310952-23313952	3000	0.43333333
chr3:23854952-23858952	4000	0.4
chr3:24029952-24032952	3000	0.56666667
chr3:25257952-25261952	4000	0.65
chr3:25413952-25416952	3000	0.6
chr3:25435952-25438952	3000	0.53333333
chr3:25907952-25911952	4000	1.25
chr3:26046952-26051952	5000	0.74
chr3:26265952-26268952	3000	0.9
chr3:26273952-26276952	3000	0.46666667
chr3:27300952-27305952	5000	0.54
chr3:28252952-28255952	3000	0.5
chr3:28304952-28308952	4000	0.35
chr3:28544952-28547952	3000	0.53333333
chr3:28949952-28952952	3000	0.6
chr3:29015952-29018952	3000	0.33333333
chr3:29418952-29422952	4000	0.575
chr3:30144852-30147852	3000	0.56666667
chr3:30989852-30992852	3000	1
chr3:31041852-31044852	3000	0.9
chr3:31392852-31395852	3000	0.3
chr3:31437852-31440852	3000	0.5
chr3:31923852-31928852	5000	1.34
chr3:34854852-34858852	4000	0.375
chr3:34915852-34918852	3000	0.46666667
chr3:34993852-34996852	3000	0.33333333
chr3:35029852-35032852	3000	0.66666667
chr3:35033852-35037852	4000	0.5
chr3:35179852-35182852	3000	0.43333333
chr3:35244852-35247852	3000	0.63333333
chr3:35835852-35838852	3000	0.7
chr3:35987852-35990852	3000	0.46666667
chr3:37316852-37319852	3000	0.53333333
chr3:38166852-38170852	4000	0.7
chr3:39760678-39764678	4000	0.825
chr3:39997678-40001678	4000	0.45
chr3:40603678-40608678	5000	1.16
chr3:41072678-41078678	6000	0.61666667
chr3:42759678-42763678	4000	0.95
chr3:42981678-42985678	4000	0.8
chr3:46722678-46725678	3000	0.6
chr3:49103678-49107678	4000	0.925
chr3:49205678-49208678	3000	0.5
chr3:54726647-54729647	3000	0.96666667
chr3:56355647-56358647	3000	0.86666667
chr3:57018647-57023647	5000	0.78
chr3:57031647-57034647	3000	1.53333333
chr3:57083647-57086647	3000	1.16666667
chr3:58010647-58013647	3000	1.2
chr3:60922116-60925116	3000	0.66666667

chr3:62207116-62210116	3000	0.46666667
chr3:64559116-64562116	3000	0.26666667
chr3:64859116-64863116	4000	1.05
chr3:65212116-65215116	3000	0.43333333
chr3:65400116-65403116	3000	0.2
chr3:65839116-65842116	3000	0.2
chr3:65999116-66002116	3000	0.33333333
chr3:66112116-66115116	3000	0.26666667
chr3:66127116-66130116	3000	0.53333333
chr3:66374116-66377116	3000	0.3
chr3:66491116-66496116	5000	0.34
chr3:67266116-67269116	3000	0.73333333
chr3:70595116-70598116	3000	0.46666667
chr3:70707116-70710116	3000	0.3
chr3:72434116-72437116	3000	0.46666667
chr3:72484116-72487116	3000	0.26666667
chr3:73424116-73427116	3000	0.33333333
chr3:73479116-73482116	3000	1.5
chr3:78984116-78987116	3000	0.4
chr3:79077116-79080116	3000	0.66666667
chr3:79596116-79599116	3000	0.5
chr3:79785116-79788116	3000	0.73333333
chr3:80638116-80641116	3000	0.53333333
chr3:80863116-80866116	3000	0.7
chr3:81078116-81081116	3000	0.43333333
chr3:82063116-82066116	3000	0.53333333
chr3:82505116-82510116	5000	0.46
chr3:85112116-85115116	3000	0.63333333
chr3:86583116-86587116	4000	0.725
chr3:92128901-92132901	4000	0.975
chr3:92662901-92665901	3000	0.6
chr3:92991901-92994901	3000	0.43333333
chr3:93006901-93013901	7000	0.55714286
chr3:93923901-93928901	5000	0.36
chr3:93996901-93999901	3000	0.36666667
chr3:94466901-94469901	3000	0.5
chr3:94915901-94918901	3000	0.6
chr3:99291901-99294901	3000	0.66666667
chr3:99382901-99385901	3000	0.8
chr3:101233901-101236901	3000	0.2
chr3:101803901-101806901	3000	0.83333333
chr3:102958901-102962901	4000	0.65
chr3:103169901-103172901	3000	0.96666667
chr3:103524901-103527901	3000	0.66666667
chr3:109293259-109296259	3000	0.6
chr3:109406259-109409259	3000	0.53333333
chr3:111064259-111067259	3000	0.5
chr3:111420259-111423259	3000	0.6
chr3:111620259-111623259	3000	1.06666667
chr3:111868259-111871259	3000	1
chr3:112100259-112106259	6000	0.5
chr3:112242259-112246259	4000	1.2
chr3:114032259-114035259	3000	0.73333333

chr3:120837278-120840278	3000	0.5
chr3:122418278-122421278	3000	0.36666667
chr3:122517278-122520278	3000	0.3
chr3:123106278-123110278	4000	0.475
chr3:123600278-123603278	3000	0.83333333
chr3:123678278-123681278	3000	0.5
chr3:123688278-123691278	3000	0.56666667
chr3:124360278-124363278	3000	0.66666667
chr3:124409278-124413278	4000	0.675
chr3:124593278-124596278	3000	0.46666667
chr3:124952278-124955278	3000	0.4
chr3:125366278-125369278	3000	0.6
chr3:125787278-125790278	3000	0.76666667
chr3:125846278-125850278	4000	0.4
chr3:125891278-125894278	3000	0.66666667
chr3:126730278-126733278	3000	0.63333333
chr3:126756278-126760278	4000	0.725
chr3:126821278-126824278	3000	0.73333333
chr3:127215278-127218278	3000	0.5
chr3:127608278-127611278	3000	0.66666667
chr3:127670278-127673278	3000	0.43333333
chr3:127933278-127936278	3000	0.43333333
chr3:128053278-128056278	3000	0.46666667
chr3:129568278-129571278	3000	0.3
chr3:129813278-129816278	3000	0.46666667
chr3:130269278-130272278	3000	0.53333333
chr3:130727278-130731278	4000	0.425
chr3:130932278-130935278	3000	0.93333333
chr3:131410278-131413278	3000	0.93333333
chr3:131552278-131557278	5000	0.74
chr3:131946278-131951278	5000	0.52
chr3:133418278-133421278	3000	0.93333333
chr3:133441278-133444278	3000	0.4
chr3:133543278-133547278	4000	0.65
chr3:135399278-135406278	7000	0.92857143
chr3:136373278-136377278	4000	0.775
chr3:136391278-136396278	5000	1.08
chr3:136605278-136608278	3000	0.7
chr3:136933278-136937278	4000	0.425
chr3:137365278-137369278	4000	0.5
chr3:140379278-140383278	4000	0.55
chr3:140440278-140443278	3000	0.56666667
chr3:149924398-149927398	3000	0.36666667
chr3:150597398-150600398	3000	0.8
chr3:151929398-151933398	4000	1.55
chr3:152791398-152795398	4000	0.975
chr3:153411398-153414398	3000	1.5
chr3:153416398-153419398	3000	0.6
chr3:161796801-161799801	3000	0.93333333
chr3:162462801-162466801	4000	0.85
chr3:165049506-165052506	3000	0.73333333
chr4:2169001-2172001	3000	0.63333333
chr4:3184001-3189001	5000	0.78

chr4:3206001-3209001	3000	1.36666667
chr4:3259001-3262001	3000	1.06666667
chr4:4036001-4039001	3000	0.53333333
chr4:4198001-4203001	5000	0.52
chr4:6731703-6734703	3000	2.83333333
chr4:8075703-8079703	4000	0.9
chr4:9501703-9504703	3000	1.33333333
chr4:11562302-11565302	3000	0.56666667
chr4:11997302-12000302	3000	0.26666667
chr4:12275302-12280302	5000	0.52
chr4:13243302-13246302	3000	0.23333333
chr4:14052302-14055302	3000	0.66666667
chr4:15094302-15097302	3000	1.13333333
chr4:15227302-15230302	3000	0.43333333
chr4:15459302-15463302	4000	0.9
chr4:15581302-15584302	3000	0.46666667
chr4:16218302-16221302	3000	0.6
chr4:31374302-31377302	3000	0.7
chr4:32106302-32109302	3000	0.4
chr4:37569302-37572302	3000	0.7
chr4:40300302-40303302	3000	0.56666667
chr4:40485302-40488302	3000	0.8
chr4:41760302-41764302	4000	0.3
chr4:42908302-42911302	3000	0.83333333
chr4:44503302-44506302	3000	0.4
chr4:45422302-45425302	3000	0.8
chr4:45647302-45650302	3000	0.46666667
chr4:48826444-48829444	3000	0.73333333
chr4:49432520-49435520	3000	0.8
chr4:50901520-50904520	3000	0.86666667
chr4:65637941-65641941	4000	0.975
chr4:65809941-65814941	5000	1.04
chr4:65838941-65842941	4000	1.25
chr4:68385941-68388941	3000	0.53333333
chr4:69458941-69462941	4000	0.4
chr4:71490941-71493941	3000	0.6
chr4:72058941-72061941	3000	0.36666667
chr4:75409941-75412941	3000	0.5
chr4:75934941-75937941	3000	0.46666667
chr4:76704299-76707299	3000	1.76666667
chr4:78358299-78361299	3000	1.06666667
chr4:78477299-78481299	4000	1.075
chr4:79619299-79623299	4000	1.6
chr4:85924316-85927316	3000	0.56666667
chr4:87419316-87423316	4000	0.3
chr4:92717316-92720316	3000	0.53333333
chr4:93985316-93988316	3000	0.6
chr4:95916316-95921316	5000	0.56
chr4:97792316-97795316	3000	0.43333333
chr4:101692316-101695316	3000	0.3
chr4:102559316-102562316	3000	0.83333333
chr4:102747316-102751316	4000	0.475
chr4:103686316-103689316	3000	0.53333333

chr4:104973747-104977747	4000	0.9
chr4:105042747-105045747	3000	0.9
chr4:106271472-106275472	4000	0.775
chr4:107303472-107306472	3000	1.3
chr4:107918472-107921472	3000	0.33333333
chr4:112143472-112146472	3000	0.26666667
chr4:112450472-112454472	4000	0.35
chr4:114415472-114420472	5000	0.48
chr4:115041472-115045472	4000	0.275
chr4:115367472-115370472	3000	0.5
chr4:115556472-115560472	4000	0.5
chr4:116481472-116484472	3000	0.76666667
chr4:116510472-116513472	3000	1.2
chr4:116517472-116521472	4000	1.55
chr4:116570472-116574472	4000	1.475
chr4:116871472-116878472	7000	0.72857143
chr4:117002472-117005472	3000	0.73333333
chr4:117181472-117184472	3000	0.93333333
chr4:117220472-117224472	4000	1.2
chr4:117929006-117933006	4000	0.725
chr4:118073006-118077006	4000	0.975
chr4:118093006-118096006	3000	0.56666667
chr4:120038308-120041308	3000	1.16666667
chr4:120490308-120495308	5000	1.2
chr4:122553876-122556876	3000	1.23333333
chr4:122865876-122868876	3000	1.16666667
chr4:123476473-123479473	3000	0.7
chr4:125240436-125243436	3000	0.93333333
chr4:126154436-126157436	3000	1.26666667
chr4:126547436-126550436	3000	1.26666667
chr4:126928436-126931436	3000	0.76666667
chr4:130306436-130309436	3000	0.93333333
chr4:132435436-132438436	3000	0.83333333
chr4:136135436-136138436	3000	0.8
chr4:136276436-136279436	3000	0.43333333
chr4:136878436-136881436	3000	1.23333333
chr4:137113436-137117436	4000	0.55
chr4:138183436-138187436	4000	0.425
chr4:142358436-142361436	3000	0.53333333
chr4:142695436-142698436	3000	0.8
chr4:143510436-143513436	3000	0.4
chr4:144004436-144007436	3000	0.76666667
chr4:146977436-146980436	3000	0.46666667
chr4:148826436-148831436	5000	0.72
chr4:149608818-149612818	4000	0.475
chr4:152625818-152628818	3000	0.8
chr4:154555248-154561248	6000	0.88333333
chr4:157323248-157326248	3000	1.13333333
chr4:160703193-160706193	3000	1.03333333
chr4:160916193-160919193	3000	0.86666667
chr4:162126660-162129660	3000	0.76666667
chr4:163392961-163395961	3000	0.93333333
chr4:163442961-163445961	3000	1.23333333

chr4:165485961-165488961	3000	0.63333333
chr4:166393961-166396961	3000	0.46666667
chr4:167840105-167843105	3000	0.2
chr4:170738105-170742105	4000	0.95
chr4:171956105-171962105	6000	1.41666667
chr4:172210105-172214105	4000	1.3
chr4:172355105-172358105	3000	1.7
chr4:172400105-172404105	4000	0.35
chr4:172596105-172599105	3000	0.66666667
chr4:173288105-173292105	4000	1.025
chr4:173324105-173328105	4000	1.5
chr4:173403105-173406105	3000	1.86666667
chr4:173414105-173417105	3000	1.26666667
chr4:173545105-173549105	4000	0.9
chr4:173760105-173766105	6000	0.95
chr4:174023105-174026105	3000	1.96666667
chr4:180500105-180503105	3000	1.26666667
chr4:180972105-180977105	5000	1.2
chr4:181005105-181009105	4000	1.35
chr4:184085105-184088105	3000	1.1
chr4:186414105-186419105	5000	1.18
chr5:267001-270001	3000	0.33333333
chr5:456001-460001	4000	0.425
chr5:2829991-2832991	3000	0.63333333
chr5:3476991-3479991	3000	0.7
chr5:3482991-3486991	4000	0.9
chr5:3557991-3561991	4000	0.875
chr5:3936991-3939991	3000	0.4
chr5:4293991-4298991	5000	1.14
chr5:4583991-4587991	4000	0.725
chr5:6348991-6353991	5000	0.38
chr5:6434991-6439991	5000	0.54
chr5:6933991-6937991	4000	0.425
chr5:7639991-7643991	4000	0.625
chr5:8032991-8035991	3000	0.46666667
chr5:8176991-8179991	3000	0.7
chr5:8282991-8285991	3000	1
chr5:9916991-9919991	3000	0.33333333
chr5:10324991-10327991	3000	0.7
chr5:10452991-10455991	3000	0.23333333
chr5:10595991-10598991	3000	0.46666667
chr5:10612991-10615991	3000	0.26666667
chr5:10624991-10629991	5000	0.18
chr5:11191991-11195991	4000	0.325
chr5:12332991-12335991	3000	1.06666667
chr5:13082991-13085991	3000	0.5
chr5:13135991-13139991	4000	0.7
chr5:13207991-13210991	3000	0.33333333
chr5:15120991-15124991	4000	0.7
chr5:15546991-15549991	3000	0.5
chr5:15697991-15701991	4000	0.475
chr5:17343991-17347991	4000	0.475
chr5:18916991-18919991	3000	0.63333333

chr5:19002991-19007991	5000	0.64
chr5:19046991-19050991	4000	0.45
chr5:19204991-19208991	4000	0.825
chr5:19274991-19277991	3000	0.53333333
chr5:19394991-19398991	4000	0.3
chr5:19567991-19571991	4000	0.85
chr5:19765846-19768846	3000	1.2
chr5:19774846-19777846	3000	0.56666667
chr5:21700846-21703846	3000	0.83333333
chr5:22088846-22092846	4000	0.475
chr5:23069846-23072846	3000	0.76666667
chr5:23175846-23179846	4000	0.5
chr5:23836846-23839846	3000	0.53333333
chr5:23949846-23952846	3000	0.66666667
chr5:23960846-23964846	4000	0.5
chr5:24169846-24172846	3000	0.33333333
chr5:24345846-24348846	3000	0.8
chr5:25420846-25424846	4000	0.775
chr5:25630846-25633846	3000	0.8
chr5:26244846-26247846	3000	0.96666667
chr5:26584846-26587846	3000	0.6
chr5:26613846-26616846	3000	1.13333333
chr5:27694846-27697846	3000	0.86666667
chr5:27793846-27796846	3000	0.66666667
chr5:27817846-27820846	3000	1.83333333
chr5:28078846-28082846	4000	0.425
chr5:29546846-29549846	3000	0.43333333
chr5:29838846-29842846	4000	0.75
chr5:31138172-31141172	3000	0.5
chr5:32178172-32181172	3000	0.7
chr5:32262172-32266172	4000	0.475
chr5:32416172-32419172	3000	0.66666667
chr5:32977172-32980172	3000	0.43333333
chr5:33063172-33066172	3000	0.6
chr5:33490172-33493172	3000	0.56666667
chr5:33513172-33516172	3000	0.6
chr5:33786172-33790172	4000	0.575
chr5:34661172-34665172	4000	0.5
chr5:34989172-34993172	4000	0.5
chr5:35078172-35081172	3000	1.36666667
chr5:36382172-36385172	3000	0.6
chr5:38174172-38178172	4000	0.475
chr5:39499172-39502172	3000	0.5
chr5:39823172-39826172	3000	0.4
chr5:39849172-39852172	3000	0.93333333
chr5:39856172-39859172	3000	0.23333333
chr5:40399172-40403172	4000	0.525
chr5:40661172-40665172	4000	0.575
chr5:40756172-40759172	3000	0.73333333
chr5:41465545-41468545	3000	0.5
chr5:41469545-41475545	6000	0.41666667
chr5:41973545-41976545	3000	0.46666667
chr5:43610545-43613545	3000	0.43333333

chr5:43947545-43950545	3000	0.46666667
chr5:43970545-43973545	3000	0.33333333
chr5:44050545-44053545	3000	0.46666667
chr5:44422545-44425545	3000	0.36666667
chr5:44710545-44716545	6000	0.48333333
chr5:45791545-45794545	3000	0.46666667
chr5:45993545-45996545	3000	0.73333333
chr5:46979545-46983545	4000	0.475
chr5:47139545-47142545	3000	0.43333333
chr5:47938545-47942545	4000	0.625
chr5:49917545-49921545	4000	0.875
chr5:49963545-49967545	4000	0.95
chr5:50338545-50341545	3000	0.4
chr5:50951545-50954545	3000	0.33333333
chr5:51016545-51023545	7000	0.71428571
chr5:51937545-51944545	7000	0.61428571
chr5:52762545-52765545	3000	0.16666667
chr5:53094545-53098545	4000	0.7
chr5:53991545-53994545	3000	0.46666667
chr5:54930545-54934545	4000	0.35
chr5:55802545-55805545	3000	0.4
chr5:56572545-56575545	3000	0.46666667
chr5:56825545-56829545	4000	0.55
chr5:57239545-57242545	3000	0.8
chr5:57243545-57246545	3000	1.46666667
chr5:57459545-57462545	3000	0.93333333
chr5:57518545-57521545	3000	0.83333333
chr5:64939261-64945261	6000	0.65
chr5:64979261-64982261	3000	0.73333333
chr5:65546261-65551261	5000	0.64
chr5:66845261-66848261	3000	0.86666667
chr5:67180261-67183261	3000	0.63333333
chr5:67417261-67420261	3000	0.5
chr5:67471261-67476261	5000	0.38
chr5:67681261-67684261	3000	0.43333333
chr5:68104261-68107261	3000	0.36666667
chr5:69724261-69727261	3000	0.36666667
chr5:70426261-70429261	3000	0.63333333
chr5:72309261-72312261	3000	0.36666667
chr5:72394261-72399261	5000	0.62
chr5:72453261-72456261	3000	0.83333333
chr5:78688458-78691458	3000	1.46666667
chr5:80825817-80828817	3000	0.66666667
chr5:82329817-82332817	3000	0.56666667
chr5:82848817-82851817	3000	0.66666667
chr5:84614817-84617817	3000	0.23333333
chr5:84739817-84742817	3000	0.56666667
chr5:100506817-100510817	4000	0.25
chr5:100722817-100726817	4000	0.55
chr5:100756817-100759817	3000	0.66666667
chr5:102601817-102605817	4000	0.425
chr5:103014817-103018817	4000	0.75
chr5:103094817-103097817	3000	0.8

chr5:103997817-104002817	5000	0.7
chr5:106712817-106716817	4000	0.5
chr5:106987817-106995817	8000	0.6
chr5:108601817-108605817	4000	0.525
chr5:108853817-108857817	4000	0.75
chr5:109772817-109775817	3000	0.2
chr5:109816817-109819817	3000	0.93333333
chr5:109860817-109865817	5000	0.46
chr5:110115817-110119817	4000	0.35
chr5:110202817-110205817	3000	0.73333333
chr5:110298817-110301817	3000	0.36666667
chr5:110480817-110483817	3000	0.7
chr5:110583817-110588817	5000	0.76
chr5:111113817-111116817	3000	0.76666667
chr5:112105817-112108817	3000	0.33333333
chr5:113575817-113578817	3000	0.56666667
chr5:114148817-114151817	3000	0.36666667
chr5:114152817-114155817	3000	0.43333333
chr5:114285817-114288817	3000	1
chr5:117063817-117066817	3000	0.5
chr5:117155817-117158817	3000	0.43333333
chr5:118522817-118526817	4000	1.225
chr5:118699817-118702817	3000	0.56666667
chr5:120051817-120054817	3000	1.56666667
chr5:122727817-122731817	4000	0.425
chr5:123028817-123032817	4000	0.65
chr5:124375817-124378817	3000	0.7
chr5:124939817-124942817	3000	0.33333333
chr5:125018817-125021817	3000	0.4
chr5:125163817-125166817	3000	0.7
chr5:127172415-127175415	3000	0.73333333
chr5:132359271-132362271	3000	0.4
chr5:132386271-132389271	3000	0.33333333
chr5:133522271-133526271	4000	0.575
chr5:134801271-134804271	3000	0.7
chr5:134862271-134866271	4000	0.675
chr5:135170271-135173271	3000	0.43333333
chr5:135960271-135964271	4000	0.525
chr5:139341039-139345039	4000	0.325
chr5:139457039-139461039	4000	0.325
chr5:147014039-147017039	3000	1.63333333
chr5:150116039-150119039	3000	1.3
chr5:163300332-163303332	3000	0.3
chr5:170129531-170134531	5000	1.22
chr5:170142531-170146531	4000	1.425
chr5:170149531-170152531	3000	2.03333333
chr5:170219531-170222531	3000	1.33333333
chr5:170258531-170262531	4000	0.75
chr5:170315531-170318531	3000	0.93333333
chr5:170517531-170520531	3000	2.46666667
chr5:170717531-170722531	5000	1.38
chr6:840001-843001	3000	0.7
chr6:2316001-2319001	3000	1.2

chr6:2383001-2386001	3000	1
chr6:2601001-2604001	3000	0.53333333
chr6:5321001-5325001	4000	0.175
chr6:6101001-6105001	4000	0.65
chr6:12686041-12689041	3000	0.93333333
chr6:12726041-12729041	3000	0.36666667
chr6:12786041-12789041	3000	0.86666667
chr6:13642041-13645041	3000	0.46666667
chr6:14432041-14435041	3000	0.73333333
chr6:16474041-16478041	4000	0.425
chr6:16549041-16552041	3000	0.2
chr6:16919041-16924041	5000	0.26
chr6:19624041-19628041	4000	0.675
chr6:21334041-21337041	3000	1
chr6:23017520-23021520	4000	0.75
chr6:28893874-28896874	3000	0.86666667
chr6:29882874-29885874	3000	0.53333333
chr6:31393874-31396874	3000	0.43333333
chr6:32667841-32670841	3000	1.03333333
chr6:33051841-33056841	5000	0.54
chr6:34005841-34008841	3000	0.56666667
chr6:34039841-34042841	3000	0.3
chr6:34093841-34096841	3000	0.9
chr6:34165841-34168841	3000	0.7
chr6:34326841-34330841	4000	0.45
chr6:34359841-34362841	3000	0.56666667
chr6:34494841-34498841	4000	0.75
chr6:35797841-35802841	5000	0.72
chr6:37027841-37031841	4000	0.825
chr6:37163841-37166841	3000	0.43333333
chr6:37173841-37176841	3000	1.06666667
chr6:37177841-37180841	3000	0.7
chr6:37227841-37230841	3000	0.76666667
chr6:37470841-37473841	3000	0.83333333
chr6:37641841-37644841	3000	0.33333333
chr6:37725841-37728841	3000	1.16666667
chr6:39113841-39117841	4000	0.475
chr6:39391841-39394841	3000	0.83333333
chr6:39525841-39528841	3000	0.86666667
chr6:39972841-39977841	5000	0.36
chr6:40872841-40875841	3000	1.03333333
chr6:44512420-44515420	3000	0.36666667
chr6:44962420-44965420	3000	0.8
chr6:45050420-45053420	3000	0.7
chr6:45496420-45499420	3000	0.5
chr6:45568420-45571420	3000	0.43333333
chr6:45747420-45750420	3000	0.7
chr6:45820420-45823420	3000	0.76666667
chr6:46094420-46097420	3000	0.76666667
chr6:46198420-46201420	3000	0.53333333
chr6:47041420-47044420	3000	0.6
chr6:47147420-47150420	3000	0.8
chr6:47422420-47426420	4000	0.7

chr6:47639420-47643420	4000	0.725
chr6:48719420-48722420	3000	0.7
chr6:48745420-48748420	3000	0.56666667
chr6:50531420-50534420	3000	0.36666667
chr6:50557420-50562420	5000	0.8
chr6:50798420-50803420	5000	0.22
chr6:50879420-50882420	3000	0.46666667
chr6:50886420-50890420	4000	0.6
chr6:51039420-51042420	3000	0.8
chr6:51862420-51865420	3000	1.1
chr6:51868420-51871420	3000	0.76666667
chr6:52520420-52523420	3000	1.13333333
chr6:52566420-52569420	3000	1.3
chr6:53220420-53223420	3000	0.5
chr6:53497420-53501420	4000	0.975
chr6:54090420-54093420	3000	0.33333333
chr6:54114420-54117420	3000	0.36666667
chr6:54465420-54468420	3000	0.4
chr6:55385420-55388420	3000	0.53333333
chr6:55567420-55571420	4000	0.525
chr6:55781420-55786420	5000	0.68
chr6:57051420-57054420	3000	0.76666667
chr6:59332420-59336420	4000	0.475
chr6:61184420-61187420	3000	0.6
chr6:61237420-61240420	3000	0.26666667
chr6:61470420-61474420	4000	0.625
chr6:62638420-62642420	4000	0.525
chr6:62786420-62789420	3000	0.43333333
chr6:63303420-63306420	3000	0.53333333
chr6:63413420-63416420	3000	0.36666667
chr6:63446420-63449420	3000	0.8
chr6:63468420-63472420	4000	0.775
chr6:64404420-64408420	4000	0.475
chr6:65279420-65283420	4000	0.125
chr6:65386420-65389420	3000	0.4
chr6:66142420-66145420	3000	0.46666667
chr6:66422420-66426420	4000	0.925
chr6:66469420-66472420	3000	0.7
chr6:68022420-68027420	5000	0.42
chr6:68595420-68599420	4000	0.375
chr6:68998420-69001420	3000	0.6
chr6:69984420-69987420	3000	0.83333333
chr6:71310420-71314420	4000	0.975
chr6:72839420-72843420	4000	0.55
chr6:74275358-74278358	3000	1.1
chr6:77801358-77810358	9000	0.55555556
chr6:77950358-77953358	3000	0.63333333
chr6:78914358-78918358	4000	0.75
chr6:79046358-79050358	4000	0.725
chr6:80161898-80164898	3000	0.43333333
chr6:81258898-81262898	4000	0.4
chr6:81616898-81619898	3000	0.53333333
chr6:82235898-82239898	4000	0.35

chr6:82273898-82277898	4000	0.65
chr6:83480898-83483898	3000	0.23333333
chr6:83910898-83913898	3000	0.26666667
chr6:85645898-85648898	3000	0.46666667
chr6:85773898-85776898	3000	0.63333333
chr6:86298898-86301898	3000	0.73333333
chr6:86619898-86622898	3000	1.16666667
chr6:86671898-86675898	4000	1
chr6:86973898-86976898	3000	0.9
chr6:87057898-87060898	3000	0.26666667
chr6:87218898-87221898	3000	0.76666667
chr6:87233898-87236898	3000	0.3
chr6:87581898-87585898	4000	0.4
chr6:89794898-89797898	3000	1.36666667
chr6:90013898-90016898	3000	0.56666667
chr6:90214898-90218898	4000	1
chr6:90321898-90324898	3000	0.43333333
chr6:93662347-93665347	3000	0.56666667
chr6:93669347-93672347	3000	0.96666667
chr6:93811347-93814347	3000	0.86666667
chr6:97010308-97014308	4000	0.825
chr6:97275308-97278308	3000	0.6
chr6:97295308-97299308	4000	0.65
chr6:97306308-97309308	3000	0.5
chr6:100237308-100240308	3000	0.36666667
chr6:105414308-105417308	3000	1.33333333
chr6:106837531-106840531	3000	1.4
chr6:113451075-113454075	3000	0.5
chr6:113963075-113966075	3000	0.56666667
chr6:114733075-114736075	3000	0.93333333
chr6:116166075-116171075	5000	0.18
chr6:118529075-118534075	5000	0.48
chr6:118744075-118748075	4000	0.6
chr6:118791075-118795075	4000	0.7
chr6:119297075-119300075	3000	0.3
chr6:120938075-120942075	4000	0.5
chr6:122073075-122076075	3000	0.8
chr6:122081075-122084075	3000	0.8
chr6:122172075-122175075	3000	0.7
chr6:122250075-122253075	3000	0.36666667
chr6:122267075-122273075	6000	1.05
chr6:122531075-122535075	4000	0.85
chr6:125861075-125865075	4000	0.2
chr6:126008075-126011075	3000	0.8
chr6:127914075-127917075	3000	0.5
chr6:127990075-127994075	4000	1
chr6:129512075-129515075	3000	1.5
chr6:130595075-130599075	4000	1.05
chr6:130887075-130890075	3000	0.66666667
chr6:131090075-131093075	3000	0.73333333
chr6:133894075-133897075	3000	0.76666667
chr6:133936075-133939075	3000	0.6
chr6:134472075-134475075	3000	0.8

chr6:135012075-135015075	3000	0.56666667
chr6:135023075-135026075	3000	0.96666667
chr6:135029075-135033075	4000	1.3
chr6:138763365-138766365	3000	0.33333333
chr6:139994365-139997365	3000	0.2
chr6:143329365-143338365	9000	0.64444444
chr6:143378365-143381365	3000	0.83333333
chr6:143937365-143941365	4000	0.625
chr6:144039365-144043365	4000	0.85
chr6:144332365-144335365	3000	0.46666667
chr6:144488365-144493365	5000	1.22
chr7:2458001-2461001	3000	0.9
chr7:3586364-3589364	3000	0.26666667
chr7:4842364-4845364	3000	0.83333333
chr7:5341364-5345364	4000	0.35
chr7:6406364-6409364	3000	0.33333333
chr7:8199364-8202364	3000	0.53333333
chr7:8349364-8352364	3000	0.5
chr7:8832364-8843364	11000	0.43636364
chr7:9016364-9019364	3000	0.5
chr7:9023364-9026364	3000	0.76666667
chr7:9415364-9418364	3000	0.5
chr7:11976364-11979364	3000	0.6
chr7:21772364-21775364	3000	0.83333333
chr7:26101364-26105364	4000	0.9
chr7:26131364-26135364	4000	1.225
chr7:26794364-26799364	5000	0.36
chr7:27079364-27082364	3000	1.03333333
chr7:27366364-27370364	4000	0.525
chr7:27473364-27477364	4000	0.625
chr7:27591364-27594364	3000	1.06666667
chr7:28633364-28637364	4000	0.575
chr7:28662364-28665364	3000	0.96666667
chr7:28775364-28778364	3000	0.56666667
chr7:33190364-33193364	3000	0.53333333
chr7:33628364-33631364	3000	1.23333333
chr7:33700364-33703364	3000	0.3
chr7:34320364-34323364	3000	0.83333333
chr7:34329364-34332364	3000	0.66666667
chr7:35202364-35207364	5000	0.52
chr7:37388364-37391364	3000	0.5
chr7:37456364-37459364	3000	0.33333333
chr7:37648364-37651364	3000	2
chr7:37857364-37861364	4000	0.375
chr7:38288862-38294862	6000	0.91666667
chr7:38475862-38478862	3000	0.76666667
chr7:39238862-39244862	6000	0.68333333
chr7:39461862-39466862	5000	0.24
chr7:40329862-40333862	4000	0.25
chr7:41276862-41281862	5000	0.6
chr7:42906862-42909862	3000	0.5
chr7:43519862-43522862	3000	0.53333333
chr7:44848862-44851862	3000	0.33333333

chr7:45021862-45027862	6000	0.46666667
chr7:45267862-45270862	3000	0.76666667
chr7:45290862-45293862	3000	0.66666667
chr7:45500862-45503862	3000	1.2
chr7:45751862-45756862	5000	0.72
chr7:47363862-47366862	3000	0.43333333
chr7:47769862-47772862	3000	0.43333333
chr7:47975862-47978862	3000	0.4
chr7:52045862-52048862	3000	0.26666667
chr7:52096862-52101862	5000	0.4
chr7:52287862-52290862	3000	0.36666667
chr7:52678862-52681862	3000	0.4
chr7:53683862-53686862	3000	0.43333333
chr7:53921862-53924862	3000	0.73333333
chr7:53928862-53931862	3000	0.93333333
chr7:54381862-54385862	4000	0.525
chr7:57644862-57647862	3000	0.63333333
chr7:57794862-57798862	4000	1.275
chr7:57828862-57831862	3000	1.46666667
chr7:57969862-57972862	3000	0.63333333
chr7:58943862-58946862	3000	0.96666667
chr7:59058862-59061862	3000	1.13333333
chr7:59292862-59296862	4000	1.2
chr7:59761862-59764862	3000	0.4
chr7:60017862-60021862	4000	0.975
chr7:61136862-61139862	3000	0.7
chr7:61919862-61922862	3000	0.5
chr7:63195862-63198862	3000	0.76666667
chr7:63267862-63270862	3000	0.93333333
chr7:63430862-63433862	3000	0.46666667
chr7:64236862-64239862	3000	0.86666667
chr7:64275862-64283862	8000	0.4375
chr7:64566862-64569862	3000	0.6
chr7:64774862-64779862	5000	0.38
chr7:65645862-65648862	3000	0.53333333
chr7:65782862-65787862	5000	1.06
chr7:66375862-66378862	3000	0.9
chr7:66471862-66474862	3000	1.76666667
chr7:66491862-66494862	3000	1.06666667
chr7:66790862-66794862	4000	0.375
chr7:67774862-67778862	4000	0.325
chr7:68196356-68201356	5000	0.74
chr7:68216356-68219356	3000	0.46666667
chr7:68699356-68703356	4000	0.6
chr7:69202356-69205356	3000	1.86666667
chr7:69215356-69218356	3000	0.56666667
chr7:73433549-73437549	4000	0.875
chr7:75648549-75651549	3000	0.73333333
chr7:75655549-75658549	3000	0.7
chr7:75704549-75708549	4000	1.15
chr7:76808254-76811254	3000	0.63333333
chr7:77283300-77287300	4000	0.325
chr7:77328300-77331300	3000	0.56666667

chr7:77543300-77547300	4000	0.425
chr7:77602300-77605300	3000	0.8
chr7:78008300-78011300	3000	0.63333333
chr7:80294050-80299050	5000	0.82
chr7:80868561-80871561	3000	0.26666667
chr7:80935561-80938561	3000	0.3
chr7:81196561-81199561	3000	0.33333333
chr7:81696561-81699561	3000	0.53333333
chr7:83310561-83313561	3000	0.6
chr7:84265968-84268968	3000	0.66666667
chr7:84767637-84771637	4000	0.575
chr7:85116637-85119637	3000	0.26666667
chr7:85557778-85560778	3000	0.2
chr7:85758778-85765778	7000	0.32857143
chr7:87939575-87942575	3000	0.56666667
chr7:88511575-88514575	3000	0.7
chr7:88532575-88535575	3000	0.56666667
chr7:88638575-88641575	3000	0.6
chr7:88703575-88708575	5000	0.78
chr7:90096575-90099575	3000	0.46666667
chr7:90669575-90672575	3000	0.63333333
chr7:91182575-91185575	3000	0.66666667
chr7:92117575-92120575	3000	0.7
chr7:92267575-92271575	4000	0.45
chr7:92442575-92445575	3000	0.56666667
chr7:93069575-93073575	4000	0.6
chr7:93973305-93976305	3000	0.63333333
chr7:95386806-95389806	3000	0.76666667
chr7:96759655-96763655	4000	0.425
chr7:97385655-97388655	3000	0.5
chr7:97842655-97845655	3000	0.43333333
chr7:98100655-98105655	5000	0.3
chr7:98327655-98330655	3000	0.76666667
chr7:99778655-99782655	4000	0.575
chr7:99817655-99821655	4000	0.375
chr7:101714655-101717655	3000	1.1
chr7:102156655-102159655	3000	0.43333333
chr7:102425655-102429655	4000	0.45
chr7:102931655-102935655	4000	0.575
chr7:103003655-103007655	4000	0.425
chr7:103403655-103406655	3000	0.56666667
chr7:103619655-103622655	3000	0.3
chr7:103630655-103634655	4000	0.275
chr7:103646655-103649655	3000	0.8
chr7:103745655-103748655	3000	0.56666667
chr7:103753655-103756655	3000	0.4
chr7:103880655-103886655	6000	0.33333333
chr7:104245655-104248655	3000	0.46666667
chr7:104298655-104301655	3000	0.5
chr7:105591655-105594655	3000	1.06666667
chr7:105598655-105601655	3000	0.46666667
chr7:105775655-105778655	3000	0.66666667
chr7:106205655-106211655	6000	0.75

chr7:106281655-106284655	3000	0.6
chr7:107424655-107427655	3000	0.5
chr7:108432655-108437655	5000	0.38
chr7:108456655-108459655	3000	0.66666667
chr7:108469655-108472655	3000	0.53333333
chr7:108566655-108569655	3000	0.93333333
chr7:108739655-108742655	3000	0.5
chr7:108961655-108964655	3000	0.63333333
chr7:108977655-108981655	4000	0.625
chr7:109033655-109036655	3000	0.26666667
chr7:109283655-109286655	3000	0.7
chr7:109340655-109343655	3000	0.6
chr7:109587655-109590655	3000	0.43333333
chr7:110183655-110186655	3000	0.7
chr7:111664655-111667655	3000	1.56666667
chr7:111812655-111815655	3000	1.33333333
chr7:112083655-112086655	3000	0.96666667
chr7:112757655-112760655	3000	1.16666667
chr7:112897655-112900655	3000	1.33333333
chr7:113057655-113060655	3000	0.8
chr7:116341655-116346655	5000	1.12
chr7:116472655-116475655	3000	1.43333333
chr7:124385877-124389877	4000	0.975
chr7:124600877-124603877	3000	1.2
chr7:124988877-124991877	3000	1.23333333
chr7:125042877-125045877	3000	0.93333333
chr7:125260877-125264877	4000	1.525
chr7:126177929-126180929	3000	0.86666667
chr7:126300929-126303929	3000	0.53333333
chr7:126466929-126471929	5000	1.32
chr7:126536929-126539929	3000	0.93333333
chr7:126834929-126839929	5000	1
chr7:126869929-126873929	4000	0.55
chr7:128770929-128773929	3000	0.9
chr7:128786929-128790929	4000	0.65
chr7:128870929-128874929	4000	1.225
chr7:129687929-129690929	3000	1.36666667
chr7:130087929-130092929	5000	0.82
chr7:130100929-130103929	3000	0.5
chr7:130554929-130559929	5000	0.8
chr7:131082752-131085752	3000	0.9
chr7:131329752-131333752	4000	1.025
chr7:132216752-132219752	3000	1.16666667
chr7:132289752-132293752	4000	1.25
chr7:132480752-132483752	3000	0.8
chr7:132533752-132536752	3000	0.5
chr7:132791752-132794752	3000	1.03333333
chr7:132983752-132986752	3000	1.7
chr7:133595752-133598752	3000	0.9
chr7:133744752-133747752	3000	1.9
chr7:134558752-134561752	3000	1.33333333
chr7:136327759-136330759	3000	2
chr8:398252-401252	3000	0.33333333

chr8:448252-451252	3000	0.43333333
chr8:558252-561252	3000	0.2
chr8:1008252-1011252	3000	0.5
chr8:1541252-1544252	3000	0.4
chr8:1602252-1605252	3000	0.66666667
chr8:2306252-2309252	3000	0.46666667
chr8:3369252-3376252	7000	0.3
chr8:3821252-3825252	4000	0.475
chr8:3920252-3923252	3000	0.5
chr8:4279252-4284252	5000	0.76
chr8:5079252-5082252	3000	0.8
chr8:5218252-5221252	3000	1.2
chr8:5218252-5221252	3000	1.2
chr8:5265252-5268252	3000	0.16666667
chr8:5280252-5283252	3000	0.26666667
chr8:6268252-6272252	4000	0.3
chr8:6268252-6272252	4000	0.3
chr8:6703252-6708252	5000	0.66
chr8:7098252-7101252	3000	0.5
chr8:7285252-7288252	3000	0.8
chr8:7911252-7914252	3000	0.73333333
chr8:8157252-8160252	3000	0.46666667
chr8:8642252-8645252	3000	0.66666667
chr8:8903252-8906252	3000	0.63333333
chr8:8923252-8926252	3000	0.83333333
chr8:8923252-8926252	3000	0.83333333
chr8:9270252-9273252	3000	0.4
chr8:9348252-9351252	3000	0.26666667
chr8:9687252-9690252	3000	0.66666667
chr8:11062252-11067252	5000	0.72
chr8:12344252-12347252	3000	2
chr8:12766252-12769252	3000	1.03333333
chr8:12815252-12818252	3000	0.56666667
chr8:12866252-12871252	5000	0.54
chr8:12866252-12871252	5000	0.54
chr8:13021252-13025252	4000	0.75
chr8:13536252-13540252	4000	0.5
chr8:13592252-13595252	3000	0.53333333
chr8:14340252-14343252	3000	0.93333333
chr8:14650252-14653252	3000	0.6
chr8:14650252-14653252	3000	0.6
chr8:17001252-17006252	5000	0.48
chr8:18129252-18132252	3000	0.2
chr8:18624252-18629252	5000	0.42
chr8:22594159-22598159	4000	0.675
chr8:23292159-23296159	4000	0.45
chr8:23488159-23494159	6000	0.63333333
chr8:24056159-24060159	4000	0.425
chr8:24631159-24634159	3000	1.5
chr8:25440159-25443159	3000	0.6
chr8:25590159-25594159	4000	0.9
chr8:25853159-25856159	3000	0.6
chr8:25953159-25959159	6000	0.5

chr8:26069159-26072159	3000	0.4
chr8:26144159-26147159	3000	0.56666667
chr8:26177159-26180159	3000	0.53333333
chr8:26523159-26526159	3000	0.6
chr8:27193159-27198159	5000	1.08
chr8:27228159-27231159	3000	0.43333333
chr8:27317159-27320159	3000	0.73333333
chr8:27913159-27916159	3000	0.3
chr8:28643159-28646159	3000	0.83333333
chr8:29098159-29101159	3000	0.93333333
chr8:29212159-29216159	4000	0.875
chr8:29947159-29950159	3000	0.63333333
chr8:29952159-29957159	5000	0.92
chr8:30134159-30137159	3000	0.86666667
chr8:30502159-30505159	3000	0.63333333
chr8:32933659-32937659	4000	0.65
chr8:33617659-33621659	4000	0.3
chr8:33899659-33902659	3000	0.76666667
chr8:34011659-34015659	4000	0.825
chr8:34276659-34279659	3000	0.73333333
chr8:35946659-35950659	4000	0.3
chr8:36745659-36748659	3000	0.46666667
chr8:39456865-39459865	3000	0.4
chr8:39926865-39929865	3000	0.46666667
chr8:40254865-40257865	3000	0.46666667
chr8:41843865-41847865	4000	0.575
chr8:42073865-42076865	3000	0.4
chr8:43192661-43195661	3000	1.3
chr8:44790034-44793034	3000	0.53333333
chr8:53659813-53663813	4000	0.75
chr8:54627813-54630813	3000	1.13333333
chr8:55245813-55248813	3000	1.03333333
chr8:55943813-55946813	3000	0.53333333
chr8:55989813-55992813	3000	1.03333333
chr8:56000813-56003813	3000	0.4
chr8:56065813-56069813	4000	0.525
chr8:80661726-80665726	4000	0.675
chr8:80903726-80907726	4000	0.525
chr8:81054726-81058726	4000	0.4
chr8:81188726-81191726	3000	0.73333333
chr8:81329726-81332726	3000	0.46666667
chr8:81799726-81803726	4000	0.45
chr8:81823726-81826726	3000	0.4
chr8:81881726-81884726	3000	0.76666667
chr8:82020726-82023726	3000	0.16666667
chr8:82355726-82358726	3000	1.23333333
chr8:82390726-82393726	3000	0.96666667
chr8:84053726-84057726	4000	0.5
chr8:85783726-85787726	4000	0.65
chr8:86903726-86906726	3000	0.73333333
chr8:87187726-87190726	3000	0.53333333
chr8:87593726-87596726	3000	0.23333333
chr8:87622726-87625726	3000	0.4

chr8:87772726-87776726	4000	0.65
chr8:87874726-87877726	3000	1.06666667
chr8:89360726-89363726	3000	1.4
chr8:91249726-91252726	3000	0.8
chr8:91612726-91615726	3000	0.5
chr8:93103726-93107726	4000	0.55
chr8:94386288-94389288	3000	0.36666667
chr8:95657288-95660288	3000	0.5
chr8:96236288-96239288	3000	0.46666667
chr8:97674288-97677288	3000	0.63333333
chr8:97735288-97739288	4000	0.425
chr8:98053288-98057288	4000	0.925
chr8:98795288-98799288	4000	0.425
chr8:99032288-99035288	3000	0.46666667
chr8:99165288-99169288	4000	0.575
chr8:99179288-99182288	3000	1.16666667
chr8:99388288-99391288	3000	0.46666667
chr8:99409288-99413288	4000	0.325
chr8:99468288-99472288	4000	0.575
chr8:100086288-100089288	3000	0.93333333
chr8:100399288-100402288	3000	1.03333333
chr8:102562288-102566288	4000	0.875
chr8:102767288-102770288	3000	0.63333333
chr8:103267288-103270288	3000	0.36666667
chr8:103494288-103499288	5000	0.72
chr8:103528288-103531288	3000	0.73333333
chr8:104855882-104858882	3000	1.23333333
chr8:105010882-105015882	5000	1.1
chr8:105156882-105159882	3000	0.96666667
chr8:106602882-106605882	3000	1
chr8:109631882-109635882	4000	1.4
chr8:110352882-110355882	3000	0.96666667
chr8:117661123-117664123	3000	0.7
chr8:120049123-120052123	3000	1.5
chr8:121972123-121976123	4000	1.5
chr9:125001-128001	3000	0.23333333
chr9:2681001-2684001	3000	0.86666667
chr9:2752001-2755001	3000	0.4
chr9:3214001-3218001	4000	0.8
chr9:3765001-3768001	3000	0.4
chr9:3956001-3959001	3000	0.43333333
chr9:4013001-4017001	4000	0.35
chr9:4609001-4613001	4000	0.7
chr9:4742001-4745001	3000	0.83333333
chr9:4976001-4980001	4000	0.725
chr9:14263948-14268948	5000	0.44
chr9:14822948-14825948	3000	0.46666667
chr9:14965948-14968948	3000	0.16666667
chr9:15114948-15118948	4000	0.9
chr9:15306948-15312948	6000	0.63333333
chr9:16748948-16751948	3000	0.4
chr9:16757948-16761948	4000	0.7
chr9:16901948-16904948	3000	0.3

chr9:19373948-19377948	4000	0.725
chr9:21784948-21787948	3000	0.6
chr9:21795948-21799948	4000	0.775
chr9:21828948-21831948	3000	0.5
chr9:21849948-21852948	3000	0.7
chr9:22365948-22369948	4000	0.475
chr9:23412948-23415948	3000	1.23333333
chr9:23532948-23535948	3000	1.4
chr9:23658948-23661948	3000	0.7
chr9:24504948-24509948	5000	0.66
chr9:24730948-24733948	3000	0.5
chr9:25091948-25094948	3000	0.46666667
chr9:25568948-25571948	3000	0.4
chr9:25768948-25773948	5000	0.62
chr9:26129948-26132948	3000	0.73333333
chr9:26147948-26150948	3000	0.43333333
chr9:26419948-26423948	4000	0.525
chr9:28402948-28405948	3000	0.26666667
chr9:29011948-29015948	4000	0.575
chr9:30344948-30348948	4000	0.525
chr9:30350948-30353948	3000	0.7
chr9:30481948-30484948	3000	0.46666667
chr9:30543948-30547948	4000	0.525
chr9:30679948-30682948	3000	0.4
chr9:30975948-30979948	4000	0.325
chr9:31134948-31137948	3000	0.9
chr9:31527948-31530948	3000	0.73333333
chr9:31540948-31543948	3000	0.6
chr9:31562948-31565948	3000	0.3
chr9:31633948-31636948	3000	0.7
chr9:32116948-32119948	3000	0.8
chr9:32129948-32132948	3000	1.23333333
chr9:32153948-32157948	4000	1
chr9:32368948-32371948	3000	1.5
chr9:33966948-33970948	4000	0.675
chr9:34018948-34021948	3000	0.53333333
chr9:37795948-37798948	3000	2.2
chr9:38136948-38139948	3000	0.9
chr9:38834948-38838948	4000	0.85
chr9:39678948-39681948	3000	0.6
chr9:39943948-39947948	4000	0.975
chr9:40102948-40106948	4000	0.6
chr9:40754948-40757948	3000	1.1
chr9:40809948-40812948	3000	0.53333333
chr9:40863948-40866948	3000	0.96666667
chr9:41605948-41608948	3000	0.8
chr9:41735948-41739948	4000	0.875
chr9:42219948-42223948	4000	1.275
chr9:42501948-42504948	3000	1.13333333
chr9:42618948-42621948	3000	1.36666667
chr9:44080948-44083948	3000	0.2
chr9:44320948-44323948	3000	0.46666667
chr9:44625948-44628948	3000	0.5

chr9:44814948-44817948	3000	0.26666667
chr9:45474948-45477948	3000	0.63333333
chr9:45497948-45501948	4000	1.025
chr9:45595948-45598948	3000	0.5
chr9:45918948-45921948	3000	0.5
chr9:47387948-47391948	4000	0.775
chr9:47561948-47564948	3000	1
chr9:48293948-48296948	3000	0.6
chr9:48746948-48749948	3000	0.7
chr9:50301948-50304948	3000	0.7
chr9:50669948-50672948	3000	0.43333333
chr9:50790948-50793948	3000	0.4
chr9:50978948-50981948	3000	0.46666667
chr9:51746948-51749948	3000	0.26666667
chr9:51769948-51773948	4000	0.575
chr9:52105948-52108948	3000	0.5
chr9:52326948-52329948	3000	0.86666667
chr9:52807948-52810948	3000	1.2
chr9:54214043-54217043	3000	0.43333333
chr9:54564043-54567043	3000	0.26666667
chr9:55039043-55042043	3000	0.93333333
chr9:55351043-55354043	3000	0.63333333
chr9:56087683-56090683	3000	1.2
chr9:56265683-56268683	3000	0.6
chr9:56502683-56505683	3000	0.7
chr9:56651683-56657683	6000	0.65
chr9:59629879-59632879	3000	0.73333333
chr9:60054879-60057879	3000	1.2
chr9:61561879-61566879	5000	0.28
chr9:64039879-64042879	3000	0.56666667
chr9:64087879-64090879	3000	0.4
chr9:64209879-64212879	3000	0.23333333
chr9:64566879-64571879	5000	0.34
chr9:66311879-66314879	3000	0.63333333
chr9:66667879-66671879	4000	0.525
chr9:67469879-67472879	3000	0.86666667
chr9:68032879-68035879	3000	0.83333333
chr9:68038879-68042879	4000	0.6
chr9:68755879-68758879	3000	0.83333333
chr9:72363876-72368876	5000	0.82
chr9:72370876-72374876	4000	0.975
chr9:72618876-72622876	4000	0.825
chr9:72686876-72693876	7000	0.75714286
chr9:72753876-72756876	3000	0.56666667
chr9:73017876-73020876	3000	0.43333333
chr9:73091876-73094876	3000	0.63333333
chr9:75483290-75486290	3000	1.16666667
chr9:75860290-75864290	4000	0.825
chr9:78672290-78675290	3000	0.23333333
chr9:79391290-79394290	3000	0.63333333
chr9:79420290-79424290	4000	1.5
chr9:79796290-79799290	3000	0.7
chr9:80103290-80106290	3000	1.03333333

chr9:80172290-80175290	3000	0.7
chr9:80377290-80380290	3000	0.6
chr9:80676290-80679290	3000	0.63333333
chr9:81250290-81253290	3000	0.13333333
chr9:83071290-83074290	3000	0.43333333
chr9:83244290-83247290	3000	0.63333333
chr9:83702290-83705290	3000	0.6
chr9:89768290-89771290	3000	1.16666667
chr9:89920290-89923290	3000	0.8
chr9:91823290-91826290	3000	0.76666667
chr9:91891290-91895290	4000	1.05
chr9:93424290-93427290	3000	1.1
chr9:93942290-93946290	4000	1.375
chr9:94423290-94426290	3000	0.56666667
chr9:94741290-94745290	4000	0.525
chr9:95580290-95586290	6000	0.56666667
chr9:95678290-95681290	3000	0.8
chr9:95699290-95702290	3000	0.73333333
chr9:95849290-95853290	4000	0.4
chr9:98722290-98725290	3000	0.5
chr9:99382290-99385290	3000	1.06666667
chr9:99522290-99525290	3000	0.83333333
chr9:99658290-99661290	3000	0.43333333
chr9:99764290-99767290	3000	0.46666667
chr9:99813290-99816290	3000	0.86666667
chr9:99947290-99950290	3000	0.4
chr9:99985290-99988290	3000	0.4
chr9:100729290-100735290	6000	0.51666667
chr9:100826290-100829290	3000	0.8
chr9:102953290-102956290	3000	0.93333333
chr9:103269290-103272290	3000	1.43333333
chr9:103991290-103994290	3000	1.46666667
chr9:104095290-104098290	3000	1.63333333
chr9:105337290-105342290	5000	0.7
chr9:106797290-106801290	4000	2
chr9:107328290-107331290	3000	0.6
chr9:107361290-107364290	3000	1.1
chr9:107447290-107451290	4000	1.5
chr9:107605290-107609290	4000	0.975
chr9:107832290-107835290	3000	0.53333333
chr9:108214290-108218290	4000	0.625
chr9:108623290-108627290	4000	0.725
chr9:108917290-108921290	4000	0.425
chr9:108966290-108970290	4000	0.65
chr9:109587290-109590290	3000	0.6
chr9:111035290-111038290	3000	0.36666667
chr9:111133290-111136290	3000	0.36666667
chr9:111680290-111685290	5000	0.32
chrX:2647001-2650001	3000	0.6
chrX:2904001-2907001	3000	0.4
chrX:3161001-3164001	3000	1.03333333
chrX:3944001-3948001	4000	0.2
chrX:8087999-8091999	4000	0.725

chrX:9754999-9757999	3000	0.36666667
chrX:10974999-10983999	9000	0.44444444
chrX:14568999-14573999	5000	0.3
chrX:14843999-14848999	5000	0.3
chrX:15826999-15830999	4000	0.425
chrX:16433999-16438999	5000	0.32
chrX:16447999-16451999	4000	0.5
chrX:16794999-16797999	3000	0.43333333
chrX:17297999-17300999	3000	0.43333333
chrX:20593999-20596999	3000	0.36666667
chrX:21866999-21869999	3000	0.63333333
chrX:21938999-21941999	3000	1.16666667
chrX:22493999-22497999	4000	0.6
chrX:22590999-22593999	3000	1.2
chrX:22606999-22609999	3000	1.26666667
chrX:23011999-23014999	3000	0.8
chrX:23168999-23171999	3000	1.06666667
chrX:23242999-23248999	6000	0.65
chrX:23350999-23355999	5000	0.68
chrX:23483999-23486999	3000	0.7
chrX:23885999-23889999	4000	0.45
chrX:24236999-24239999	3000	0.7
chrX:24754999-24758999	4000	0.35
chrX:25712999-25718999	6000	0.71666667
chrX:26084999-26087999	3000	0.36666667
chrX:27719077-27723077	4000	0.525
chrX:28246077-28249077	3000	0.6
chrX:34263077-34266077	3000	0.66666667
chrX:34521077-34524077	3000	0.93333333
chrX:35062093-35065093	3000	0.76666667
chrX:36041093-36044093	3000	0.86666667
chrX:37749093-37752093	3000	0.8
chrX:41065093-41068093	3000	0.66666667
chrX:41184093-41188093	4000	0.9
chrX:45928093-45931093	3000	0.66666667
chrX:46087093-46090093	3000	1.13333333
chrX:46565093-46570093	5000	0.72
chrX:47949361-47952361	3000	0.63333333
chrX:49494361-49497361	3000	0.43333333
chrX:49549361-49553361	4000	0.4
chrX:50616361-50619361	3000	0.46666667
chrX:50940361-50943361	3000	0.63333333
chrX:51390361-51393361	3000	0.66666667
chrX:52414361-52417361	3000	0.93333333
chrX:54803361-54806361	3000	0.43333333
chrX:55010361-55013361	3000	0.8
chrX:56603361-56606361	3000	0.5
chrX:61473209-61476209	3000	0.4
chrX:62970209-62973209	3000	0.7
chrX:63518209-63521209	3000	0.73333333
chrX:64531209-64534209	3000	0.73333333
chrX:66718209-66721209	3000	0.53333333
chrX:67043209-67046209	3000	0.2

chrX:67054209-67057209	3000	0.36666667
chrX:72499209-72504209	5000	0.42
chrX:74306209-74311209	5000	0.28
chrX:79719209-79722209	3000	0.4
chrX:80268209-80271209	3000	0.33333333
chrX:81744209-81747209	3000	0.83333333
chrX:82552209-82555209	3000	0.4
chrX:82901209-82905209	4000	0.875
chrX:85892209-85895209	3000	0.76666667
chrX:86796209-86800209	4000	0.6
chrX:87009209-87013209	4000	0.85
chrX:89246209-89249209	3000	0.5
chrX:89710430-89713430	3000	0.7
chrX:90613430-90618430	5000	0.66
chrX:90632430-90635430	3000	0.43333333
chrX:91176430-91180430	4000	0.75
chrX:93286430-93290430	4000	0.525
chrX:93810430-93813430	3000	0.16666667
chrX:94046430-94049430	3000	1.16666667
chrX:95621430-95625430	4000	0.55
chrX:97315430-97319430	4000	0.5
chrX:98140430-98143430	3000	0.73333333
chrX:98785430-98788430	3000	0.46666667
chrX:98916430-98919430	3000	0.46666667
chrX:99790430-99793430	3000	0.43333333
chrX:100817430-100820430	3000	0.7
chrX:101612430-101615430	3000	0.5
chrX:101686430-101689430	3000	0.43333333
chrX:108192430-108195430	3000	0.16666667
chrX:108513430-108517430	4000	0.25
chrX:108609430-108612430	3000	0.36666667
chrX:111708430-111711430	3000	0.26666667
chrX:113775430-113778430	3000	0.36666667
chrX:119290430-119294430	4000	0.4
chrX:119779430-119782430	3000	0.5
chrX:122769470-122772470	3000	0.53333333
chrX:125774510-125777510	3000	0.36666667
chrX:126328510-126331510	3000	0.56666667
chrX:126936510-126939510	3000	0.23333333
chrX:127582510-127585510	3000	0.63333333
chrX:128440510-128443510	3000	0.96666667
chrX:132579510-132582510	3000	0.36666667
chrX:132737510-132740510	3000	0.3
chrX:133683510-133686510	3000	0.43333333
chrX:133709510-133712510	3000	0.5
chrX:134407510-134410510	3000	0.6
chrX:134412510-134415510	3000	0.46666667
chrX:134870510-134873510	3000	1
chrX:137845510-137848510	3000	0.7
chrX:143820510-143823510	3000	0.83333333
chrX:145264510-145267510	3000	0.6
chrX:146085510-146088510	3000	0.46666667
chrX:146818510-146822510	4000	0.375

chrX:147242510-147245510	3000	0.46666667
chrX:147539510-147542510	3000	0.3
chrX:149811510-149815510	4000	0.5
chrX:152269510-152272510	3000	0.23333333
chrX:154822510-154825510	3000	0.26666667
chrX:155151510-155155510	4000	0.425
chrX:156938510-156941510	3000	0.56666667
chrX:159729510-159733510	4000	0.6
chrX:159989510-159992510	3000	0.66666667

(B) Sertoli-Granulosa final list of susceptible DMRs (includes 3+ consecutive sites only) (1503)

Potential DMR site	Length	CpG/100bp
chr1:401001-405001	4000	2.525
chr1:558001-561001	3000	2.8
chr1:660001-664001	4000	1.025
chr1:846001-849001	3000	2.76666667
chr1:1069001-1073001	4000	3.775
chr1:1127001-1131001	4000	0.725
chr1:2205001-2208001	3000	1.16666667
chr1:2928364-2931364	3000	0.43333333
chr1:4537364-4540364	3000	0.5
chr1:4665364-4668364	3000	1.9
chr1:7018364-7022364	4000	1.75
chr1:7776364-7781364	5000	1.74
chr1:7829364-7832364	3000	0.86666667
chr1:10267364-10271364	4000	1.45
chr1:10393364-10396364	3000	3
chr1:11423364-11427364	4000	1.6
chr1:12036364-12040364	4000	0.475
chr1:13055364-13058364	3000	0.96666667
chr1:13921364-13925364	4000	1.2
chr1:15570364-15576364	6000	1.4
chr1:18837364-18841364	4000	0.725
chr1:19975364-19979364	4000	0.3
chr1:21487364-21492364	5000	1.42
chr1:21843364-21847364	4000	1.95
chr1:21907364-21910364	3000	0.53333333
chr1:21948364-21951364	3000	1.3
chr1:23438364-23442364	4000	0.925
chr1:25061364-25064364	3000	1.76666667
chr1:26744364-26749364	5000	1.32
chr1:27032364-27036364	4000	1.925
chr1:27203364-27207364	4000	0.85
chr1:29318364-29321364	3000	0.36666667
chr1:29641364-29644364	3000	1.66666667
chr1:32550364-32553364	3000	1.46666667
chr1:33271364-33275364	4000	0.3
chr1:40087862-40090862	3000	0.86666667
chr1:43617862-43620862	3000	0.3
chr1:45708862-45712862	4000	1
chr1:46273862-46276862	3000	1.5
chr1:47567862-47572862	5000	1.44
chr1:48163862-48168862	5000	1.42
chr1:49864862-49868862	4000	1.15
chr1:54491862-54495862	4000	1.5
chr1:56597862-56601862	4000	0.775
chr1:58235862-58239862	4000	1.675
chr1:58790862-58793862	3000	1.66666667
chr1:59303862-59306862	3000	0.3
chr1:61906862-61910862	4000	1.225
chr1:64995862-64998862	3000	1

chr1:65079862-65083862	4000	1.5
chr1:69328356-69332356	4000	1.325
chr1:70057549-70060549	3000	1.33333333
chr1:70550549-70553549	3000	3.03333333
chr1:70726549-70730549	4000	1.575
chr1:70984549-70987549	3000	0.36666667
chr1:72720549-72723549	3000	1.56666667
chr1:72918549-72923549	5000	0.8
chr1:73099549-73102549	3000	1.96666667
chr1:74268549-74271549	3000	0.56666667
chr1:74387549-74391549	4000	2.325
chr1:77996300-78000300	4000	0.375
chr1:79118686-79123686	5000	1.22
chr1:80800095-80804095	4000	1.35
chr1:80811095-80814095	3000	1.1
chr1:82989561-82995561	6000	1.6
chr1:83028561-83033561	5000	0.4
chr1:83328561-83331561	3000	1.2
chr1:84721637-84726637	5000	1.28
chr1:84813637-84816637	3000	0.56666667
chr1:84903637-84906637	3000	1.56666667
chr1:85334778-85337778	3000	0.73333333
chr1:85906444-85909444	3000	1.46666667
chr1:86344444-86347444	3000	0.96666667
chr1:86348444-86353444	5000	0.32
chr1:92795575-92798575	3000	0.43333333
chr1:93516575-93519575	3000	1.33333333
chr1:94181305-94188305	7000	0.64285714
chr1:94502305-94506305	4000	0.85
chr1:98268655-98271655	3000	0.7
chr1:98392655-98395655	3000	1.66666667
chr1:102346655-102349655	3000	0.5
chr1:104577655-104581655	4000	0.45
chr1:105393655-105397655	4000	0.675
chr1:110606655-110610655	4000	0.6
chr1:111342655-111345655	3000	1.86666667
chr1:114350655-114353655	3000	1
chr1:115038655-115041655	3000	1.1
chr1:116811655-116814655	3000	0.96666667
chr1:117534655-117539655	5000	1.5
chr1:120997877-121000877	3000	0.56666667
chr1:129088929-129093929	5000	1.22
chr1:130585929-130588929	3000	0.63333333
chr1:130968752-130973752	5000	1.36
chr1:133066752-133069752	3000	1.63333333
chr1:137155759-137158759	3000	0.43333333
chr1:142653187-142658187	5000	0.36
chr1:145462187-145465187	3000	1.7
chr1:146549187-146552187	3000	0.6
chr1:146645042-146649042	4000	1.05
chr1:155264042-155268042	4000	0.575
chr1:155478042-155482042	4000	0.4
chr1:157187211-157190211	3000	1.03333333

chr1:157520211-157524211	4000	0.65
chr1:159078211-159081211	3000	0.96666667
chr1:160151975-160157975	6000	1.23333333
chr1:162596803-162599803	3000	0.56666667
chr1:163659846-163664846	5000	2.56
chr1:163758846-163761846	3000	1.56666667
chr1:168047550-168053550	6000	1.38333333
chr1:169830550-169833550	3000	0.73333333
chr1:171985550-171992550	7000	1.04285714
chr1:178016061-178023061	7000	0.84285714
chr1:179875061-179878061	3000	0.63333333
chr1:180614061-180618061	4000	1.35
chr1:185107061-185110061	3000	1.43333333
chr1:186018919-186021919	3000	1.33333333
chr1:186634919-186638919	4000	0.8
chr1:186986919-186989919	3000	0.96666667
chr1:189876919-189879919	3000	0.96666667
chr1:199976432-199979432	3000	1.06666667
chr1:202547803-202550803	3000	0.73333333
chr1:202981545-202984545	3000	1.33333333
chr1:208257267-208261267	4000	0.65
chr1:211208677-211212677	4000	1.525
chr1:213382321-213385321	3000	0.83333333
chr1:216963879-216969879	6000	0.26666667
chr1:220379879-220382879	3000	1.7
chr1:220551879-220554879	3000	0.33333333
chr1:224078879-224081879	3000	1.13333333
chr1:232616745-232619745	3000	1
chr1:233174745-233180745	6000	1.51666667
chr1:233188745-233195745	7000	0.9
chr1:237857745-237860745	3000	0.66666667
chr1:238428745-238431745	3000	0.66666667
chr1:241016745-241020745	4000	0.35
chr1:246124808-246127808	3000	0.53333333
chr1:247915808-247919808	4000	0.325
chr1:247931808-247934808	3000	1.03333333
chr1:249015403-249021403	6000	1.01666667
chr1:250020054-250023054	3000	1.3
chr1:251511850-251516850	5000	1.04
chr1:254375776-254378776	3000	1.26666667
chr1:263143846-263146846	3000	0.63333333
chr1:265218846-265222846	4000	0.675
chr2:668001-674001	6000	0.95
chr2:2306001-2309001	3000	1.5
chr2:2820001-2824001	4000	1.075
chr2:2825001-2828001	3000	1.53333333
chr2:3110001-3113001	3000	0.46666667
chr2:6948001-6952001	4000	1.15
chr2:7050001-7054001	4000	0.35
chr2:8092001-8097001	5000	2.7
chr2:8305001-8309001	4000	1.775
chr2:8871001-8875001	4000	0.675
chr2:9881001-9884001	3000	0.3

chr2:10063001-10067001	4000	0.875
chr2:13932001-13935001	3000	0.6
chr2:14425001-14428001	3000	0.46666667
chr2:14476001-14479001	3000	1
chr2:14610001-14613001	3000	1.83333333
chr2:16427001-16433001	6000	1.3
chr2:17166001-17169001	3000	1.5
chr2:17772001-17776001	4000	0.4
chr2:18680001-18683001	3000	2.5
chr2:22365001-22369001	4000	0.7
chr2:23168001-23173001	5000	1.58
chr2:23196001-23200001	4000	0.5
chr2:23295001-23299001	4000	1.35
chr2:24116001-24119001	3000	0.8
chr2:32311655-32314655	3000	1.13333333
chr2:33059655-33062655	3000	0.2
chr2:37745655-37750655	5000	1.56
chr2:38250655-38253655	3000	1.26666667
chr2:43617655-43620655	3000	0.56666667
chr2:44468655-44474655	6000	1.28333333
chr2:45222655-45225655	3000	1.16666667
chr2:51878655-51881655	3000	1.2
chr2:54527655-54531655	4000	0.875
chr2:55522655-55525655	3000	0.93333333
chr2:58927311-58932311	5000	1.6
chr2:64933724-64936724	3000	1.63333333
chr2:65310724-65313724	3000	0.33333333
chr2:66039724-66042724	3000	1.9
chr2:67059724-67062724	3000	0.53333333
chr2:70511724-70514724	3000	1.7
chr2:70725724-70728724	3000	1.03333333
chr2:76478724-76481724	3000	1.43333333
chr2:77225724-77230724	5000	0.76
chr2:80215724-80218724	3000	0.93333333
chr2:84246724-84249724	3000	0.46666667
chr2:86012724-86015724	3000	1.4
chr2:86304724-86307724	3000	1.13333333
chr2:86316724-86319724	3000	0.5
chr2:86452724-86456724	4000	2.2
chr2:86548724-86552724	4000	1.525
chr2:87103724-87106724	3000	0.5
chr2:87465724-87468724	3000	1.63333333
chr2:93428724-93431724	3000	1.1
chr2:94894724-94899724	5000	1.12
chr2:96849724-96852724	3000	0.66666667
chr2:100470724-100473724	3000	0.53333333
chr2:103883724-103888724	5000	0.96
chr2:104219724-104222724	3000	0.36666667
chr2:105053724-105056724	3000	0.3
chr2:105616724-105619724	3000	0.83333333
chr2:105875724-105878724	3000	0.43333333
chr2:107518724-107521724	3000	0.5
chr2:108655724-108659724	4000	1.15

chr2:112712724-112716724	4000	0.85
chr2:113766724-113769724	3000	0.73333333
chr2:116128724-116131724	3000	0.96666667
chr2:118163112-118166112	3000	0.3
chr2:119346541-119352541	6000	1.95
chr2:122591541-122595541	4000	0.525
chr2:122870541-122873541	3000	0.76666667
chr2:123816541-123819541	3000	1
chr2:124536541-124539541	3000	0.2
chr2:128827541-128831541	4000	2.1
chr2:130214541-130217541	3000	0.3
chr2:136832541-136838541	6000	1.06666667
chr2:138591541-138594541	3000	0.76666667
chr2:140088541-140091541	3000	1.2
chr2:144315541-144318541	3000	0.56666667
chr2:145350541-145354541	4000	0.225
chr2:145926541-145929541	3000	0.4
chr2:149231110-149234110	3000	0.7
chr2:149361110-149364110	3000	0.53333333
chr2:150664110-150670110	6000	1.16666667
chr2:159734110-159737110	3000	1.6
chr2:160530110-160536110	6000	1.46666667
chr2:165119110-165122110	3000	0.56666667
chr2:165811110-165814110	3000	0.5
chr2:166303110-166306110	3000	1.43333333
chr2:167409110-167412110	3000	0.4
chr2:169449110-169452110	3000	1.3
chr2:169492110-169495110	3000	0.33333333
chr2:172245110-172250110	5000	1.32
chr2:180801275-180804275	3000	0.7
chr2:180942275-180946275	4000	1.25
chr2:183649893-183652893	3000	1.06666667
chr2:184632893-184636893	4000	1.475
chr2:189570893-189573893	3000	0.7
chr2:189700893-189707893	7000	1
chr2:189790893-189793893	3000	0.86666667
chr2:190307893-190310893	3000	1.3
chr2:191071186-191075186	4000	1.55
chr2:191208186-191212186	4000	0.975
chr2:191905508-191909508	4000	0.6
chr2:193219286-193223286	4000	1.175
chr2:193753286-193758286	5000	1.42
chr2:197157286-197162286	5000	1.18
chr2:198538652-198541652	3000	0.3
chr2:199714652-199717652	3000	0.53333333
chr2:200247536-200251536	4000	1.2
chr2:201700536-201705536	5000	1.34
chr2:201999536-202002536	3000	0.4
chr2:204701186-204705186	4000	0.375
chr2:206941249-206944249	3000	0.33333333
chr2:207182249-207185249	3000	0.66666667
chr2:208301249-208304249	3000	2.66666667
chr2:209843249-209846249	3000	0.96666667

chr2:209898249-209902249	4000	0.375
chr2:210310249-210313249	3000	0.4
chr2:210518249-210522249	4000	0.875
chr2:213317249-213323249	6000	1.61666667
chr2:216034249-216037249	3000	2.66666667
chr2:216955249-216958249	3000	0.76666667
chr2:218012249-218015249	3000	1.4
chr2:218508249-218511249	3000	1.06666667
chr2:222001205-222004205	3000	1.76666667
chr2:223388205-223391205	3000	0.56666667
chr2:226761205-226764205	3000	1.53333333
chr2:228189205-228192205	3000	1.03333333
chr2:229937202-229942202	5000	1.34
chr2:230805202-230813202	8000	1
chr2:235556202-235560202	4000	1.375
chr2:236227202-236230202	3000	1.33333333
chr2:237456202-237459202	3000	1.06666667
chr2:240106202-240112202	6000	1.4
chr2:240433202-240440202	7000	1.34285714
chr2:249965202-249968202	3000	0.63333333
chr2:251721202-251725202	4000	3.025
chr2:255923112-255927112	4000	0.95
chr2:256990112-256993112	3000	0.76666667
chr2:257401112-257407112	6000	1.46666667
chr3:1193001-1197001	4000	0.25
chr3:2130001-2134001	4000	0.775
chr3:2737001-2740001	3000	0.7
chr3:5614626-5619626	5000	0.86
chr3:12913943-12916943	3000	1.16666667
chr3:14015943-14018943	3000	0.8
chr3:14848699-14851699	3000	1.3
chr3:16461851-16464851	3000	0.23333333
chr3:16959388-16962388	3000	0.93333333
chr3:17408388-17412388	4000	0.8
chr3:19107952-19111952	4000	0.525
chr3:19211952-19214952	3000	0.23333333
chr3:19614952-19617952	3000	0.56666667
chr3:20442952-20445952	3000	0.73333333
chr3:21728952-21731952	3000	1.46666667
chr3:22221952-22224952	3000	1.66666667
chr3:25324952-25327952	3000	0.5
chr3:27411952-27415952	4000	1.05
chr3:27854952-27857952	3000	1.73333333
chr3:28468952-28472952	4000	2.05
chr3:28488952-28491952	3000	0.53333333
chr3:30974852-30979852	5000	1.1
chr3:34262852-34266852	4000	0.15
chr3:34267852-34270852	3000	0.6
chr3:37365852-37368852	3000	1.3
chr3:38485852-38489852	4000	1.225
chr3:44010678-44013678	3000	0.16666667
chr3:44026678-44030678	4000	0.9
chr3:50242678-50248678	6000	0.33333333

chr3:51222678-51226678	4000	0.65
chr3:52770647-52773647	3000	0.96666667
chr3:53706647-53709647	3000	0.36666667
chr3:55157647-55160647	3000	0.76666667
chr3:58399116-58402116	3000	0.93333333
chr3:59320116-59324116	4000	1.25
chr3:66643116-66646116	3000	0.83333333
chr3:66741116-66745116	4000	1.575
chr3:67671116-67674116	3000	1.33333333
chr3:68746116-68749116	3000	0.4
chr3:68929116-68932116	3000	0.26666667
chr3:73798116-73802116	4000	1.1
chr3:77250116-77253116	3000	1.36666667
chr3:80734116-80737116	3000	0.56666667
chr3:83780116-83783116	3000	0.46666667
chr3:84416116-84420116	4000	1.075
chr3:85064116-85067116	3000	1.2
chr3:89119116-89122116	3000	1
chr3:90002375-90012375	10000	0.67
chr3:90807901-90810901	3000	0.8
chr3:92022901-92026901	4000	0.5
chr3:93752901-93755901	3000	0.3
chr3:95492901-95496901	4000	0.575
chr3:97463901-97466901	3000	1.6
chr3:100088901-100092901	4000	1.05
chr3:105313577-105316577	3000	0.3
chr3:109285259-109288259	3000	2.26666667
chr3:109317259-109320259	3000	1.1
chr3:109323259-109326259	3000	0.43333333
chr3:110134259-110138259	4000	2.4
chr3:116208278-116211278	3000	0.53333333
chr3:117045278-117048278	3000	0.4
chr3:117128278-117132278	4000	0.55
chr3:118704278-118710278	6000	0.7
chr3:124394278-124397278	3000	1
chr3:133079278-133082278	3000	0.73333333
chr3:135125278-135129278	4000	0.475
chr3:137391278-137394278	3000	0.66666667
chr3:137837278-137842278	5000	1.5
chr3:140649278-140653278	4000	0.725
chr3:142855186-142859186	4000	0.9
chr3:145390571-145396571	6000	1.43333333
chr3:149085174-149088174	3000	1.46666667
chr3:155313826-155317826	4000	0.925
chr3:155396074-155399074	3000	0.83333333
chr3:156549074-156552074	3000	1.33333333
chr3:158406074-158411074	5000	1.24
chr3:158567074-158571074	4000	1.625
chr3:159603824-159609824	6000	1.16666667
chr3:160575824-160578824	3000	0.83333333
chr3:161859801-161863801	4000	1.35
chr3:163988506-163991506	3000	0.9
chr4:75525941-75533941	8000	0.825

chr4:38575302-38578302	3000	0.93333333
chr4:154671248-154686248	15000	1.48666667
chr4:121872876-121882876	10000	1.27
chr4:181026105-181033105	7000	1.45714286
chr4:134664436-134671436	7000	1.37142857
chr4:142194436-142204436	10000	0.62
chr4:108153472-108157472	4000	1.275
chr4:165698961-165701961	3000	1.16666667
chr4:102273316-102282316	9000	0.36666667
chr4:2656001-2665001	9000	0.87777778
chr4:65379941-65384941	5000	1.18
chr4:78767299-78777299	10000	0.94
chr4:8999703-9007703	8000	1.75
chr4:113984472-113988472	4000	0.225
chr4:157700248-157704248	4000	1.45
chr4:186587105-186590105	3000	0.5
chr4:114003472-114013472	10000	0.64
chr4:110158472-110173472	15000	0.44666667
chr4:155601248-155606248	5000	1.28
chr4:118075006-118083006	8000	0.9125
chr4:6412703-6417703	5000	1.32
chr4:155864248-155871248	7000	1.2
chr4:116857472-116871472	14000	1.1
chr4:48696444-48701444	5000	0.56
chr4:144226436-144229436	3000	0.83333333
chr4:76312941-76327941	15000	0.59333333
chr4:106272472-106278472	6000	0.93333333
chr4:182947105-182952105	5000	1.34
chr4:95471316-95478316	7000	0.45714286
chr4:65164941-65174941	10000	0.31
chr4:184835105-184840105	5000	0.84
chr4:134741436-134750436	9000	0.6
chr4:179530105-179547105	17000	0.71176471
chr4:105406747-105415747	9000	1.17777778
chr4:147750436-147753436	3000	0.93333333
chr4:116808472-116816472	8000	0.925
chr4:173724105-173735105	11000	1.25454545
chr4:151219818-151224818	5000	0.46
chr4:186420105-186437105	17000	0.91176471
chr4:185551105-185563105	12000	0.93333333
chr4:65235941-65249941	14000	1.02142857
chr4:186594105-186600105	6000	0.53333333
chr4:4952001-4963001	11000	0.45454545
chr4:116368472-116382472	14000	1.45714286
chr4:65220941-65233941	13000	0.59230769
chr4:581001-587001	6000	0.4
chr4:116787472-116802472	15000	1.08
chr4:106295472-106303472	8000	1.15
chr4:154435248-154439248	4000	0.975
chr4:2637001-2642001	5000	1
chr4:116821472-116833472	12000	1.2
chr4:175761105-175770105	9000	1.18888889
chr4:6477703-6487703	10000	1.42

chr4:6239703-6249703	10000	1.01
chr4:135207436-135222436	15000	0.96
chr4:1004001-1007001	3000	0.63333333
chr4:4160001-4165001	5000	0.64
chr4:134862436-134865436	3000	0.8
chr4:77807299-77814299	7000	0.78571429
chr4:173318105-173324105	6000	1.1
chr4:78502299-78513299	11000	1.27272727
chr4:139284436-139292436	8000	0.6875
chr4:1080001-1083001	3000	0.66666667
chr4:105294747-105307747	13000	1.23076923
chr4:143195436-143206436	11000	0.69090909
chr4:174027105-174043105	16000	1.49375
chr4:183022105-183038105	16000	1.7625
chr4:76477941-76482941	5000	1.42
chr4:77818299-77826299	8000	0.7375
chr4:88472316-88479316	7000	0.4
chr4:156819248-156825248	6000	0.85
chr4:171891105-171898105	7000	1.1
chr4:126987436-126991436	4000	1.05
chr4:65209941-65213941	4000	1.1
chr4:92092316-92107316	15000	0.45333333
chr4:177964105-177978105	14000	1.42857143
chr4:122144876-122160876	16000	0.93125
chr4:135334436-135341436	7000	0.8
chr4:185098105-185115105	17000	1.78823529
chr4:78975299-78988299	13000	1.2
chr4:186380105-186388105	8000	0.825
chr4:46753302-46759302	6000	0.61666667
chr4:115593472-115596472	3000	0.46666667
chr4:126553436-126556436	3000	0.76666667
chr4:120483308-120493308	10000	0.72
chr4:6295703-6301703	6000	1.53333333
chr5:134001-137001	3000	1.4
chr5:910001-915001	5000	1.44
chr5:1273001-1276001	3000	0.66666667
chr5:1335001-1340001	5000	2.6
chr5:2205991-2210991	5000	1.4
chr5:2751991-2755991	4000	1.075
chr5:5367991-5371991	4000	1.375
chr5:5386991-5389991	3000	1.2
chr5:6173991-6177991	4000	1.1
chr5:6229991-6234991	5000	0.62
chr5:6809991-6812991	3000	0.33333333
chr5:7872991-7876991	4000	1.4
chr5:10528991-10532991	4000	2.175
chr5:11077991-11082991	5000	1.82
chr5:11563991-11567991	4000	0.5
chr5:13248991-13252991	4000	1.8
chr5:15772991-15776991	4000	2.275
chr5:15880991-15884991	4000	1.7
chr5:18381991-18385991	4000	2.1
chr5:18487991-18490991	3000	0.66666667

chr5:21601846-21605846	4000	2.025
chr5:22457846-22460846	3000	0.4
chr5:22469846-22472846	3000	0.9
chr5:24490846-24493846	3000	0.46666667
chr5:24786846-24789846	3000	0.76666667
chr5:25283846-25286846	3000	1.3
chr5:25433846-25436846	3000	0.4
chr5:28817846-28821846	4000	0.625
chr5:29772846-29775846	3000	1.36666667
chr5:30127846-30130846	3000	0.53333333
chr5:30778172-30781172	3000	1.43333333
chr5:31801172-31805172	4000	2.625
chr5:33322172-33326172	4000	1.4
chr5:35790172-35793172	3000	1.93333333
chr5:36212172-36216172	4000	0.5
chr5:37728172-37733172	5000	1.36
chr5:40078172-40082172	4000	0.95
chr5:41866545-41869545	3000	1.4
chr5:42889545-42893545	4000	1.025
chr5:44115545-44120545	5000	0.46
chr5:46323545-46326545	3000	1.56666667
chr5:49397545-49400545	3000	1.8
chr5:49747545-49750545	3000	0.8
chr5:50742545-50745545	3000	1.16666667
chr5:51890545-51895545	5000	1.4
chr5:51997545-52000545	3000	0.53333333
chr5:55466545-55470545	4000	1.35
chr5:55545545-55549545	4000	1.575
chr5:57182545-57185545	3000	1.23333333
chr5:58163545-58166545	3000	2
chr5:62518261-62524261	6000	1.28333333
chr5:63686261-63690261	4000	0.8
chr5:63761261-63764261	3000	0.83333333
chr5:65380261-65384261	4000	1.975
chr5:65847261-65851261	4000	0.475
chr5:71572261-71575261	3000	2.03333333
chr5:73242261-73247261	5000	1.1
chr5:73333261-73338261	5000	1.48
chr5:76961458-76964458	3000	0.96666667
chr5:77239458-77243458	4000	0.575
chr5:78443458-78447458	4000	1.225
chr5:78507458-78511458	4000	1.775
chr5:78551458-78554458	3000	1.4
chr5:78647458-78651458	4000	1.2
chr5:78725458-78728458	3000	2.1
chr5:78749458-78753458	4000	0.95
chr5:78773458-78777458	4000	1.35
chr5:78939458-78943458	4000	1.225
chr5:79033458-79037458	4000	1.975
chr5:79174458-79178458	4000	0.975
chr5:79479060-79483060	4000	1.025
chr5:79547825-79550825	3000	0.83333333
chr5:81442817-81448817	6000	1.03333333

chr5:83269817-83273817	4000	0.55
chr5:105582817-105585817	3000	0.66666667
chr5:105655817-105659817	4000	1.6
chr5:110003817-110007817	4000	0.525
chr5:113394817-113397817	3000	0.9
chr5:114908817-114911817	3000	0.7
chr5:127571415-127575415	4000	0.775
chr5:129273271-129276271	3000	1.43333333
chr5:129951271-129954271	3000	0.33333333
chr5:130901271-130907271	6000	1.11666667
chr5:135522271-135527271	5000	1.38
chr5:139394039-139397039	3000	0.93333333
chr5:142006039-142009039	3000	1.03333333
chr5:142112039-142117039	5000	1.2
chr5:142133039-142136039	3000	0.53333333
chr5:149144039-149147039	3000	0.8
chr5:151031039-151038039	7000	0.98571429
chr5:154780131-154783131	3000	1.26666667
chr5:155278131-155283131	5000	0.9
chr5:162249332-162255332	6000	1.25
chr5:163771855-163774855	3000	0.53333333
chr5:163811855-163817855	6000	1.45
chr5:164168855-164172855	4000	0.725
chr5:164652855-164655855	3000	1.5
chr5:172737591-172740591	3000	0.86666667
chr5:172860571-172865571	5000	0.86
chr6:3005001-3008001	3000	1.06666667
chr6:5401001-5405001	4000	1.65
chr6:5482001-5485001	3000	0.6
chr6:5939001-5942001	3000	1.33333333
chr6:7862001-7865001	3000	0.86666667
chr6:9427001-9431001	4000	0.425
chr6:10351001-10354001	3000	0.6
chr6:10683041-10687041	4000	1.85
chr6:10706041-10716041	10000	1.01
chr6:10927041-10931041	4000	0.675
chr6:11125041-11128041	3000	1.03333333
chr6:11301041-11304041	3000	0.5
chr6:12887041-12890041	3000	0.5
chr6:17644041-17647041	3000	1.86666667
chr6:18110041-18113041	3000	0.36666667
chr6:18214041-18217041	3000	0.13333333
chr6:18512041-18516041	4000	1.875
chr6:19168041-19171041	3000	0.43333333
chr6:19214041-19217041	3000	0.66666667
chr6:21326041-21332041	6000	1.26666667
chr6:22262520-22268520	6000	1.61666667
chr6:26602658-26605658	3000	0.7
chr6:28221874-28224874	3000	0.76666667
chr6:34398841-34401841	3000	0.83333333
chr6:40776841-40781841	5000	1.14
chr6:42478420-42484420	6000	0.96666667
chr6:48950420-48954420	4000	1.05

chr6:50396420-50399420	3000	0.63333333
chr6:50523420-50529420	6000	1.15
chr6:51885420-51889420	4000	1.725
chr6:57178420-57182420	4000	1.2
chr6:58957420-58961420	4000	0.425
chr6:61555420-61558420	3000	0.7
chr6:64070420-64073420	3000	0.93333333
chr6:64518420-64522420	4000	0.4
chr6:64819420-64824420	5000	0.36
chr6:67713420-67716420	3000	0.86666667
chr6:68135420-68139420	4000	1.35
chr6:68578420-68584420	6000	1.61666667
chr6:69030420-69035420	5000	1.52
chr6:70791420-70797420	6000	1.25
chr6:75017358-75020358	3000	1.26666667
chr6:75546358-75549358	3000	1
chr6:76487358-76492358	5000	1.48
chr6:79410358-79414358	4000	0.675
chr6:81767898-81770898	3000	0.36666667
chr6:81812898-81815898	3000	2.26666667
chr6:82005898-82008898	3000	0.4
chr6:84691898-84694898	3000	0.63333333
chr6:86533898-86538898	5000	1.12
chr6:86543898-86547898	4000	1.85
chr6:87337898-87341898	4000	1.825
chr6:89369898-89373898	4000	0.275
chr6:89603898-89606898	3000	0.63333333
chr6:91626898-91630898	4000	1.025
chr6:93897347-93900347	3000	1.93333333
chr6:94443347-94447347	4000	1.325
chr6:94785308-94788308	3000	1.06666667
chr6:100780308-100787308	7000	1.71428571
chr6:101129308-101132308	3000	0.23333333
chr6:103494308-103499308	5000	1
chr6:103531308-103535308	4000	0.875
chr6:103581308-103584308	3000	0.46666667
chr6:103604308-103607308	3000	0.6
chr6:103638308-103641308	3000	0.66666667
chr6:103676308-103679308	3000	0.63333333
chr6:103903308-103907308	4000	0.65
chr6:103964308-103969308	5000	0.56
chr6:103972308-103976308	4000	0.55
chr6:103978308-103981308	3000	0.66666667
chr6:104013308-104018308	5000	0.8
chr6:104165308-104168308	3000	0.53333333
chr6:104172308-104175308	3000	0.63333333
chr6:104190308-104193308	3000	0.93333333
chr6:104214308-104217308	3000	1.06666667
chr6:105666531-105671531	5000	1.14
chr6:105749531-105753531	4000	0.6
chr6:106274531-106278531	4000	0.925
chr6:106497531-106503531	6000	1.86666667
chr6:108126844-108130844	4000	1.325

chr6:109098703-109102703	4000	1.275
chr6:109654703-109657703	3000	1.33333333
chr6:111910075-111913075	3000	1.03333333
chr6:118638075-118643075	5000	1.5
chr6:127721075-127724075	3000	0.46666667
chr6:133040075-133046075	6000	1.11666667
chr6:136260075-136263075	3000	1.43333333
chr6:140332365-140335365	3000	0.6
chr6:141706365-141710365	4000	0.8
chr6:142317365-142320365	3000	0.26666667
chr6:142457365-142461365	4000	1.6
chr6:143729365-143734365	5000	1.24
chr6:145001365-145007365	6000	0.81666667
chr6:145104365-145108365	4000	0.9
chr7:977001-980001	3000	1.03333333
chr7:1104001-1108001	4000	1.075
chr7:2600364-2603364	3000	1.66666667
chr7:2825364-2829364	4000	0.425
chr7:3243364-3247364	4000	0.55
chr7:3781364-3785364	4000	1.6
chr7:4391364-4394364	3000	0.5
chr7:5239364-5242364	3000	0.63333333
chr7:5505364-5508364	3000	2.8
chr7:5777364-5780364	3000	0.53333333
chr7:5866364-5869364	3000	0.3
chr7:5987364-5992364	5000	1.74
chr7:6223364-6228364	5000	1.68
chr7:7055364-7059364	4000	1.125
chr7:7154364-7158364	4000	1.525
chr7:8027364-8031364	4000	0.35
chr7:9298364-9302364	4000	0.525
chr7:9318364-9321364	3000	0.73333333
chr7:9401364-9404364	3000	0.4
chr7:9481364-9485364	4000	0.65
chr7:11710364-11714364	4000	1.25
chr7:11942364-11945364	3000	1.73333333
chr7:12256364-12260364	4000	0.475
chr7:13213364-13217364	4000	1.325
chr7:13224364-13228364	4000	1.925
chr7:13262364-13265364	3000	1.4
chr7:15037364-15040364	3000	1.33333333
chr7:15051364-15055364	4000	1.575
chr7:15578364-15581364	3000	0.76666667
chr7:16569364-16572364	3000	1.36666667
chr7:17011364-17015364	4000	1.5
chr7:17267364-17270364	3000	1.73333333
chr7:17566364-17569364	3000	0.23333333
chr7:17881364-17884364	3000	1.8
chr7:18031364-18034364	3000	0.6
chr7:18910364-18913364	3000	2.36666667
chr7:18995364-18999364	4000	1.6
chr7:19094364-19098364	4000	1.475
chr7:20446364-20451364	5000	1.24

chr7:21714364-21719364	5000	1.98
chr7:21770364-21773364	3000	0.6
chr7:22386364-22390364	4000	3.5
chr7:24103364-24106364	3000	2.5
chr7:28753364-28756364	3000	0.8
chr7:30701364-30704364	3000	0.73333333
chr7:38054862-38057862	3000	1.6
chr7:39719862-39722862	3000	0.5
chr7:41480862-41486862	6000	1.15
chr7:42642862-42645862	3000	1.2
chr7:48547862-48550862	3000	1.96666667
chr7:49662862-49665862	3000	1.73333333
chr7:49882862-49885862	3000	0.56666667
chr7:50022862-50028862	6000	1.31666667
chr7:57395862-57398862	3000	1.83333333
chr7:58637862-58641862	4000	0.725
chr7:65666862-65670862	4000	0.45
chr7:68075356-68078356	3000	1.36666667
chr7:68112356-68115356	3000	0.43333333
chr7:72897549-72903549	6000	0.41666667
chr7:73241549-73245549	4000	1.05
chr7:73646549-73649549	3000	1.13333333
chr7:74278549-74282549	4000	1.125
chr7:75063549-75069549	6000	1.6
chr7:77287300-77290300	3000	0.66666667
chr7:77531300-77536300	5000	0.72
chr7:78667323-78670323	3000	1.43333333
chr7:79787711-79792711	5000	1.18
chr7:83253561-83256561	3000	1.53333333
chr7:83799968-83802968	3000	0.43333333
chr7:83967968-83970968	3000	1.9
chr7:86119444-86122444	3000	1
chr7:86328444-86331444	3000	0.6
chr7:86692444-86695444	3000	0.8
chr7:88205575-88211575	6000	1.45
chr7:88255575-88259575	4000	1.025
chr7:92596575-92599575	3000	1.3
chr7:93824305-93828305	4000	1.625
chr7:96015819-96019819	4000	2.475
chr7:97478655-97483655	5000	1.68
chr7:104901655-104904655	3000	0.26666667
chr7:107450655-107454655	4000	0.375
chr7:110170655-110173655	3000	0.16666667
chr7:110700655-110704655	4000	1.775
chr7:113283655-113287655	4000	1.325
chr7:113302655-113305655	3000	0.8
chr7:113415655-113419655	4000	1.6
chr7:113454655-113458655	4000	1.1
chr7:128822929-128826929	4000	0.85
chr7:129155929-129158929	3000	0.96666667
chr7:129776929-129779929	3000	1
chr7:134993752-134996752	3000	1.1
chr7:137168759-137171759	3000	0.5

chr7:139209759-139212759	3000	0.7
chr7:139579759-139583759	4000	1.75
chr7:140342759-140345759	3000	0.63333333
chr7:140378759-140382759	4000	0.725
chr8:240252-244252	4000	0.675
chr8:820252-824252	4000	1.625
chr8:1662252-1665252	3000	0.16666667
chr8:2999252-3002252	3000	0.26666667
chr8:3360252-3365252	5000	1.26
chr8:6263252-6267252	4000	0.9
chr8:6425252-6428252	3000	0.8
chr8:7826252-7830252	4000	0.7
chr8:9090252-9095252	5000	1.48
chr8:9811252-9814252	3000	0.86666667
chr8:11205252-11208252	3000	1.06666667
chr8:11644252-11647252	3000	0.76666667
chr8:13705252-13710252	5000	0.4
chr8:13988252-13991252	3000	0.43333333
chr8:14319252-14323252	4000	3.35
chr8:16335252-16339252	4000	1
chr8:17829252-17834252	5000	1.16
chr8:18603252-18606252	3000	0.53333333
chr8:18772252-18777252	5000	1.16
chr8:19162252-19165252	3000	1.8
chr8:19313252-19316252	3000	2.06666667
chr8:19370252-19374252	4000	1.25
chr8:23514159-23517159	3000	0.36666667
chr8:24005159-24008159	3000	1.4
chr8:29186159-29190159	4000	1.175
chr8:30214159-30217159	3000	2.03333333
chr8:31795659-31799659	4000	0.925
chr8:35751659-35754659	3000	1.3
chr8:35893659-35898659	5000	1.54
chr8:35937659-35943659	6000	1.18333333
chr8:35977659-35980659	3000	3.23333333
chr8:36087659-36090659	3000	1.36666667
chr8:36707659-36710659	3000	0.43333333
chr8:36731659-36735659	4000	0.575
chr8:36805659-36809659	4000	1.625
chr8:36920659-36923659	3000	0.43333333
chr8:36944659-36948659	4000	0.825
chr8:37002659-37006659	4000	1.575
chr8:37108659-37112659	4000	0.525
chr8:37261659-37264659	3000	0.46666667
chr8:37944659-37947659	3000	0.76666667
chr8:39600865-39604865	4000	1.825
chr8:40908865-40912865	4000	0.425
chr8:40933865-40936865	3000	0.33333333
chr8:42877865-42880865	3000	1.06666667
chr8:46987034-46990034	3000	0.9
chr8:47627555-47631555	4000	0.75
chr8:53613813-53616813	3000	0.5
chr8:56187813-56192813	5000	1.5

chr8:57596813-57599813	3000	0.26666667
chr8:61097400-61101400	4000	0.45
chr8:67157121-67161121	4000	0.825
chr8:69012121-69015121	3000	1.53333333
chr8:69595121-69598121	3000	0.33333333
chr8:70319121-70322121	3000	1.16666667
chr8:75666726-75669726	3000	0.46666667
chr8:76906726-76909726	3000	1.06666667
chr8:80729726-80733726	4000	0.825
chr8:81601726-81604726	3000	0.4
chr8:81622726-81625726	3000	0.5
chr8:83232726-83237726	5000	0.88
chr8:83262726-83267726	5000	0.86
chr8:83277726-83281726	4000	0.9
chr8:84355726-84358726	3000	0.53333333
chr8:86501726-86505726	4000	1.525
chr8:89001726-89004726	3000	0.6
chr8:92756726-92759726	3000	0.6
chr8:93924288-93928288	4000	2.125
chr8:94362288-94365288	3000	2.13333333
chr8:94410288-94414288	4000	1.4
chr8:94908288-94911288	3000	0.6
chr8:95404288-95408288	4000	1.05
chr8:99601288-99606288	5000	1.62
chr8:109129882-109132882	3000	1.1
chr8:110258882-110262882	4000	1.4
chr8:114363565-114366565	3000	0.33333333
chr8:114844532-114849532	5000	0.98
chr8:116126123-116129123	3000	1.33333333
chr8:119820123-119823123	3000	0.56666667
chr8:124203123-124207123	4000	1.075
chr8:124765123-124768123	3000	1.56666667
chr8:126551576-126554576	3000	0.7
chr9:398001-401001	3000	0.96666667
chr9:1053001-1057001	4000	2.175
chr9:1551001-1555001	4000	1.275
chr9:3675001-3678001	3000	0.86666667
chr9:4747001-4751001	4000	1.575
chr9:7224001-7228001	4000	0.95
chr9:10170001-10173001	3000	1.06666667
chr9:10178001-10182001	4000	0.95
chr9:10183001-10186001	3000	1.8
chr9:13553948-13556948	3000	0.7
chr9:16724948-16727948	3000	0.53333333
chr9:25886948-25890948	4000	1.675
chr9:27391948-27394948	3000	0.46666667
chr9:27553948-27556948	3000	1.2
chr9:29562948-29565948	3000	1.2
chr9:33147948-33153948	6000	1.16666667
chr9:36794948-36799948	5000	2.06
chr9:39973948-39976948	3000	0.43333333
chr9:41586948-41593948	7000	1.5
chr9:43317948-43320948	3000	0.5

chr9:44855948-44858948	3000	0.56666667
chr9:45192948-45195948	3000	1.83333333
chr9:46069948-46072948	3000	0.3
chr9:49947948-49950948	3000	0.23333333
chr9:52096948-52100948	4000	1.075
chr9:54640043-54643043	3000	1.4
chr9:54796043-54799043	3000	1.8
chr9:54925043-54928043	3000	0.36666667
chr9:59200879-59203879	3000	0.83333333
chr9:70592879-70596879	4000	1.275
chr9:70887879-70890879	3000	0.66666667
chr9:71393879-71396879	3000	0.96666667
chr9:73986178-73990178	4000	0.6
chr9:74844290-74847290	3000	1.5
chr9:75032290-75035290	3000	1.36666667
chr9:76383290-76387290	4000	0.925
chr9:77376290-77380290	4000	1.5
chr9:80812290-80816290	4000	1.675
chr9:80840290-80843290	3000	1.8
chr9:90373290-90377290	4000	0.6
chr9:91713290-91716290	3000	0.46666667
chr9:93525290-93528290	3000	0.66666667
chr9:96702290-96705290	3000	0.56666667
chr9:100621290-100625290	4000	0.55
chr9:102581290-102585290	4000	1.7
chr9:104276290-104281290	5000	1.82
chr9:104815290-104818290	3000	0.4
chr9:109118290-109122290	4000	0.625
chr9:109917290-109920290	3000	1.96666667
chr9:111595290-111601290	6000	0.36666667
chr9:111604290-111608290	4000	0.4
chr10:150001-154001	4000	0.3
chr10:521001-524001	3000	0.8
chr10:1664802-1667802	3000	0.63333333
chr10:1960219-1964219	4000	1.65
chr10:4331219-4334219	3000	1.7
chr10:6273219-6277219	4000	0.9
chr10:6570219-6573219	3000	1.66666667
chr10:6576219-6580219	4000	1.025
chr10:8029219-8034219	5000	1.78
chr10:10429422-10433422	4000	1.35
chr10:10791422-10794422	3000	0.56666667
chr10:11866422-11870422	4000	1.325
chr10:11973422-11977422	4000	1.25
chr10:12216422-12220422	4000	1.825
chr10:12303422-12308422	5000	0.5
chr10:12524422-12529422	5000	0.36
chr10:16996364-17003364	7000	1.08571429
chr10:23967714-23970714	3000	0.43333333
chr10:28732714-28735714	3000	1.16666667
chr10:29575714-29580714	5000	1.66
chr10:36766420-36769420	3000	0.66666667
chr10:39367720-39372720	5000	0.5

chr10:44564550-44567550	3000	1
chr10:44573550-44577550	4000	1.35
chr10:44778550-44781550	3000	1.4
chr10:55995850-56000850	5000	1.98
chr10:56002850-56005850	3000	1.43333333
chr10:60876885-60879885	3000	0.23333333
chr10:69418127-69421127	3000	1.56666667
chr10:69490127-69493127	3000	0.46666667
chr10:72313127-72316127	3000	1.16666667
chr10:76143202-76146202	3000	1.26666667
chr10:80518106-80524106	6000	1.46666667
chr10:82946106-82949106	3000	0.73333333
chr10:86614611-86617611	3000	0.5
chr10:87807611-87810611	3000	0.83333333
chr10:88840611-88844611	4000	0.45
chr10:90834611-90837611	3000	1.66666667
chr10:94328097-94333097	5000	0.74
chr10:98389097-98392097	3000	1.13333333
chr10:104767643-104770643	3000	0.66666667
chr10:104819643-104825643	6000	1.3
chr10:105117643-105121643	4000	3.025
chr10:109438636-109441636	3000	1.66666667
chr10:110338636-110341636	3000	0.93333333
chr11:335001-338001	3000	1.53333333
chr11:636001-640001	4000	1.2
chr11:2040001-2046001	6000	1.48333333
chr11:2207001-2212001	5000	1.7
chr11:8179456-8182456	3000	0.5
chr11:9336456-9340456	4000	3.25
chr11:11854456-11857456	3000	0.9
chr11:12320456-12324456	4000	1.325
chr11:12428456-12432456	4000	1.625
chr11:14517456-14520456	3000	0.7
chr11:16031456-16034456	3000	0.56666667
chr11:17682625-17685625	3000	0.56666667
chr11:18386625-18389625	3000	0.4
chr11:20762191-20766191	4000	1.9
chr11:22688191-22691191	3000	0.46666667
chr11:22851191-22855191	4000	2.1
chr11:23156191-23160191	4000	1.525
chr11:23771191-23774191	3000	1.23333333
chr11:23900191-23911191	11000	1.59090909
chr11:31923191-31927191	4000	1.25
chr11:37376191-37379191	3000	0.9
chr11:37848191-37852191	4000	0.825
chr11:38008191-38012191	4000	1.15
chr11:38774191-38777191	3000	0.56666667
chr11:38912191-38916191	4000	0.8
chr11:44426191-44430191	4000	2.15
chr11:45295191-45298191	3000	1.23333333
chr11:48439191-48442191	3000	0.73333333
chr11:50707191-50710191	3000	0.3
chr11:50916191-50919191	3000	0.5

chr11:57642800-57645800	3000	0.96666667
chr11:63606306-63611306	5000	1.26
chr11:64110306-64115306	5000	1.26
chr11:64808306-64811306	3000	0.96666667
chr11:65955306-65959306	4000	0.525
chr11:66393306-66397306	4000	1.15
chr11:66446306-66449306	3000	0.66666667
chr11:66770306-66773306	3000	1.53333333
chr11:67552306-67555306	3000	0.7
chr11:70013306-70016306	3000	1.06666667
chr11:72522306-72525306	3000	1.86666667
chr11:77664495-77667495	3000	0.9
chr11:79326495-79329495	3000	0.43333333
chr11:79976495-79979495	3000	1.1
chr11:80164495-80168495	4000	1.95
chr11:80992495-80996495	4000	1.875
chr11:80997495-81000495	3000	1.56666667
chr11:81516495-81523495	7000	1.52857143
chr11:81611495-81615495	4000	1.025
chr11:82190495-82193495	3000	1.76666667
chr11:82344495-82349495	5000	1.28
chr11:82871228-82877228	6000	1.11666667
chr11:83299228-83302228	3000	0.66666667
chr11:83610228-83613228	3000	2.43333333
chr11:84025458-84029458	4000	1.3
chr11:86211794-86215794	4000	0.8
chr12:15732837-15735837	3000	1
chr12:18091864-18095864	4000	2.6
chr12:18590864-18594864	4000	1.35
chr12:18750864-18753864	3000	1.53333333
chr12:18880864-18888864	8000	1.5875
chr12:19004864-19007864	3000	1.36666667
chr12:19212864-19217864	5000	1.42
chr12:19473864-19476864	3000	1.4
chr12:19932329-19936329	4000	1.825
chr12:20111329-20114329	3000	1.13333333
chr12:20380329-20383329	3000	1.4
chr12:20490329-20493329	3000	1.23333333
chr12:20501329-20505329	4000	1.175
chr12:20581329-20585329	4000	2.025
chr12:20592329-20595329	3000	0.3
chr12:22498303-22501303	3000	0.66666667
chr12:22515303-22518303	3000	1.03333333
chr12:22520303-22523303	3000	0.73333333
chr12:32342483-32345483	3000	2
chr12:34984929-34987929	3000	0.6
chr12:35512929-35516929	4000	0.85
chr12:46148806-46152806	4000	1.2
chr12:46170806-46175806	5000	0.92
chr13:933001-936001	3000	1.43333333
chr13:1345001-1348001	3000	1.46666667
chr13:1596001-1600001	4000	0.375
chr13:1687001-1690001	3000	0.3

chr13:2649001-2652001	3000	1.36666667
chr13:2667001-2671001	4000	1.9
chr13:3384001-3387001	3000	2.53333333
chr13:3497001-3500001	3000	0.56666667
chr13:4393001-4397001	4000	1.2
chr13:4913001-4916001	3000	0.63333333
chr13:5651001-5654001	3000	0.26666667
chr13:5928001-5931001	3000	0.3
chr13:6332001-6335001	3000	1.4
chr13:6368001-6371001	3000	0.13333333
chr13:6742001-6746001	4000	1.125
chr13:6822001-6827001	5000	0.72
chr13:7498001-7504001	6000	1.4
chr13:7512001-7515001	3000	0.33333333
chr13:8574001-8577001	3000	1.46666667
chr13:8717001-8721001	4000	1.725
chr13:9043001-9047001	4000	1.975
chr13:9049001-9052001	3000	0.4
chr13:9104001-9108001	4000	2.75
chr13:9213001-9216001	3000	1.2
chr13:9752001-9755001	3000	0.7
chr13:10157001-10160001	3000	1.53333333
chr13:11287001-11290001	3000	0.76666667
chr13:11537001-11540001	3000	0.5
chr13:12288001-12291001	3000	0.86666667
chr13:13071001-13075001	4000	1.675
chr13:13169001-13173001	4000	1.225
chr13:14590001-14593001	3000	0.26666667
chr13:15838001-15843001	5000	0.56
chr13:16235001-16240001	5000	1.1
chr13:16243001-16246001	3000	1.2
chr13:17368001-17373001	5000	2.52
chr13:17619001-17623001	4000	1
chr13:17638001-17641001	3000	1.26666667
chr13:18320001-18324001	4000	2.075
chr13:19357001-19360001	3000	1.46666667
chr13:19553001-19557001	4000	0.325
chr13:20248001-20251001	3000	0.63333333
chr13:20304001-20309001	5000	1.14
chr13:20546001-20549001	3000	0.96666667
chr13:20884001-20888001	4000	1.275
chr13:21389001-21392001	3000	0.9
chr13:21517001-21522001	5000	2.04
chr13:21990001-21995001	5000	1.34
chr13:22014001-22017001	3000	0.73333333
chr13:22677001-22683001	6000	1.8
chr13:23182001-23185001	3000	0.33333333
chr13:23675001-23681001	6000	0.85
chr13:23889001-23892001	3000	1.06666667
chr13:24780001-24786001	6000	1.13333333
chr13:25348001-25352001	4000	0.375
chr13:25695001-25698001	3000	1.1
chr13:25727001-25730001	3000	0.43333333

chr13:26225001-26230001	5000	1.54
chr13:26234001-26237001	3000	1.2
chr13:26330001-26336001	6000	1.43333333
chr13:26516001-26520001	4000	0.725
chr13:27104001-27108001	4000	2.8
chr13:27536001-27540001	4000	2.075
chr13:27543001-27547001	4000	1.3
chr13:28291001-28295001	4000	0.9
chr13:28401001-28404001	3000	1.3
chr13:28448001-28452001	4000	1.325
chr13:30015001-30019001	4000	1.575
chr13:30270001-30274001	4000	0.725
chr13:34535001-34538001	3000	1.46666667
chr13:34807001-34810001	3000	0.7
chr13:35166001-35169001	3000	0.26666667
chr13:40988001-40991001	3000	1.76666667
chr13:43199001-43203001	4000	1.7
chr13:44716796-44719796	3000	0.83333333
chr13:53951711-53955711	4000	1.225
chr13:56725711-56729711	4000	1.325
chr13:58602711-58605711	3000	0.26666667
chr13:59148711-59152711	4000	0.25
chr13:59373711-59376711	3000	0.46666667
chr13:62525711-62529711	4000	1.95
chr13:63336711-63339711	3000	2.33333333
chr13:63557711-63561711	4000	1.825
chr13:65335711-65338711	3000	1.76666667
chr13:66406711-66409711	3000	1.13333333
chr13:69348506-69351506	3000	1.6
chr13:72445978-72448978	3000	0.43333333
chr13:74948978-74952978	4000	1.8
chr13:76866978-76872978	6000	1.33333333
chr13:78286978-78290978	4000	0.925
chr13:80817978-80820978	3000	0.63333333
chr13:81479978-81483978	4000	0.65
chr13:87877175-87880175	3000	0.7
chr13:89364175-89368175	4000	1.025
chr13:93714444-93720444	6000	0.7
chr13:98203444-98207444	4000	0.9
chr13:98216444-98219444	3000	0.96666667
chr13:98799444-98803444	4000	1.1
chr13:100849291-100852291	3000	0.53333333
chr13:104663291-104666291	3000	0.63333333
chr13:107744850-107747850	3000	0.2
chr14:201001-205001	4000	0.75
chr14:517001-520001	3000	1.56666667
chr14:1502171-1505171	3000	1.06666667
chr14:4838512-4843512	5000	1.92
chr14:5739512-5742512	3000	1.56666667
chr14:5762512-5766512	4000	1.65
chr14:5819512-5824512	5000	1.3
chr14:5894512-5900512	6000	1.66666667
chr14:6410512-6416512	6000	1.75

chr14:11718844-11721844	3000	2.33333333
chr14:15248844-15251844	3000	0.4
chr14:17096844-17102844	6000	1.03333333
chr14:21412844-21415844	3000	1.23333333
chr14:22082844-22086844	4000	2.025
chr14:22093844-22096844	3000	0.9
chr14:22111844-22115844	4000	1.825
chr14:22268844-22273844	5000	1.56
chr14:22666844-22670844	4000	2.5
chr14:25994844-25997844	3000	0.76666667
chr14:30029844-30032844	3000	1.63333333
chr14:34297844-34301844	4000	1.05
chr14:37045617-37048617	3000	1.13333333
chr14:37268617-37273617	5000	1.44
chr14:44631901-44635901	4000	1.525
chr14:44661901-44664901	3000	1.8
chr14:47832901-47835901	3000	0.7
chr14:48489901-48492901	3000	1.43333333
chr14:48522901-48525901	3000	1.5
chr14:48582901-48585901	3000	1.4
chr14:48645901-48648901	3000	1.66666667
chr14:49153901-49156901	3000	1.56666667
chr14:49209901-49212901	3000	1.66666667
chr14:49528901-49532901	4000	2.1
chr14:49569901-49573901	4000	1.15
chr14:49683901-49687901	4000	1.925
chr14:51559901-51564901	5000	1.4
chr14:51787901-51790901	3000	1.06666667
chr14:52175901-52178901	3000	0.96666667
chr14:52468901-52471901	3000	1.03333333
chr14:56106901-56110901	4000	1.35
chr14:57976901-57979901	3000	2.3
chr14:65730901-65733901	3000	0.56666667
chr14:66221901-66225901	4000	1.375
chr14:72602985-72605985	3000	0.86666667
chr14:77398985-77401985	3000	0.66666667
chr14:81965836-81968836	3000	0.7
chr14:82280836-82283836	3000	0.6
chr14:82505836-82510836	5000	0.82
chr14:86209958-86213958	4000	0.85
chr14:87581747-87587747	6000	1.38333333
chr14:87632747-87635747	3000	0.7
chr14:90732747-90736747	4000	0.6
chr14:90892747-90896747	4000	0.6
chr14:94975747-94978747	3000	0.83333333
chr14:98309632-98313632	4000	1.35
chr14:98348632-98351632	3000	0.66666667
chr14:98773632-98776632	3000	1.16666667
chr14:99047632-99050632	3000	0.9
chr14:99637632-99640632	3000	0.46666667
chr14:100540632-100543632	3000	0.13333333
chr14:100564632-100567632	3000	1.2
chr14:100600632-100603632	3000	1.16666667

chr14:101072632-101075632	3000	0.36666667
chr14:102015271-102019271	4000	1.45
chr14:103026271-103029271	3000	1.03333333
chr14:108290879-108294879	4000	0.825
chr14:109476879-109479879	3000	0.53333333
chr15:4139911-4142911	3000	1.26666667
chr15:5573911-5577911	4000	1.525
chr15:5666911-5679663	12752	1.29391468
chr15:6359663-6362663	3000	1.33333333
chr15:6424663-6429663	5000	1.16
chr15:7475663-7479663	4000	0.8
chr15:7755663-7759663	4000	0.45
chr15:8523663-8529663	6000	0.58333333
chr15:9831663-9836663	5000	1.14
chr15:11634663-11638663	4000	0.375
chr15:12836663-12839663	3000	0.93333333
chr15:14106663-14111663	5000	1.72
chr15:15345760-15350760	5000	0.68
chr15:15783760-15786760	3000	0.46666667
chr15:18626760-18629760	3000	0.86666667
chr15:23265471-23270471	5000	1.28
chr15:25585471-25588471	3000	0.86666667
chr15:27170471-27176471	6000	1.21666667
chr15:27215471-27221471	6000	1.58333333
chr15:28213794-28216794	3000	0.9
chr15:29760794-29766794	6000	1.21666667
chr15:30303794-30308794	5000	1.04
chr15:32340794-32343794	3000	0.36666667
chr15:33588794-33593794	5000	1.1
chr15:34263794-34268794	5000	1.36
chr15:34779794-34784794	5000	1.84
chr15:34788794-34791794	3000	0.7
chr15:35840466-35843466	3000	2.03333333
chr15:38697466-38703466	6000	0.75
chr15:40354466-40357466	3000	2.23333333
chr15:42360466-42365466	5000	1.34
chr15:46143972-46146972	3000	0.63333333
chr15:46746786-46750786	4000	1.35
chr15:47573786-47579786	6000	1.21666667
chr15:47676786-47683786	7000	1.4
chr15:47989786-47992786	3000	1.26666667
chr15:48721786-48725786	4000	1.975
chr15:49227786-49230786	3000	1.33333333
chr15:49517786-49520786	3000	0.53333333
chr15:50116786-50120786	4000	2.05
chr15:50797107-50803107	6000	1.1
chr15:53111107-53117107	6000	1.75
chr15:54964107-54969107	5000	1.36
chr15:55188107-55192107	4000	0.5
chr15:55238107-55241107	3000	0.16666667
chr15:61043478-61047478	4000	1.35
chr15:62116478-62119478	3000	0.53333333
chr15:63643478-63646478	3000	1.33333333

chr15:64292478-64298478	6000	1.15
chr15:64867478-64870478	3000	0.6
chr15:65684478-65688478	4000	0.65
chr15:66108478-66112478	4000	1.925
chr15:69194478-69197478	3000	0.7
chr15:71436478-71442478	6000	1.15
chr15:74041478-74046478	5000	1.54
chr15:74629478-74632478	3000	1.63333333
chr15:77203478-77206478	3000	0.5
chr15:79841478-79844478	3000	0.53333333
chr15:86653478-86656478	3000	0.6
chr15:90531478-90535478	4000	2.175
chr15:91016478-91019478	3000	0.76666667
chr15:91983478-91989478	6000	1.65
chr15:93450478-93453478	3000	0.6
chr15:99088478-99091478	3000	1.4
chr15:102838478-102843478	5000	0.94
chr15:105160238-105165238	5000	2.06
chr15:106946093-106952093	6000	1.6
chr15:107080093-107084093	4000	1.025
chr16:1554001-1558001	4000	1.525
chr16:2796001-2800001	4000	1.625
chr16:2817001-2823001	6000	1.4
chr16:3069001-3074001	5000	1.68
chr16:7042607-7046607	4000	1.875
chr16:7503607-7508607	5000	1
chr16:7558607-7561607	3000	1.3
chr16:7578607-7582607	4000	1.525
chr16:8958607-8961607	3000	1.23333333
chr16:13344607-13348607	4000	1.025
chr16:14030644-14033644	3000	0.63333333
chr16:14076644-14081644	5000	1.9
chr16:16368644-16371644	3000	0.26666667
chr16:17738644-17742644	4000	0.425
chr16:18602644-18608644	6000	0.91666667
chr16:20688199-20692199	4000	0.975
chr16:24112199-24115199	3000	2.63333333
chr16:25137199-25142199	5000	1.74
chr16:25855199-25861199	6000	1.25
chr16:27448199-27451199	3000	1.83333333
chr16:28727199-28731199	4000	1.075
chr16:29371199-29374199	3000	1.4
chr16:29411199-29415199	4000	0.425
chr16:30723950-30726950	3000	0.4
chr16:31113950-31117950	4000	1.575
chr16:31398950-31402950	4000	1.5
chr16:31725950-31729950	4000	0.55
chr16:35744950-35748950	4000	1.425
chr16:40251950-40257950	6000	1.53333333
chr16:47285950-47289950	4000	3.325
chr16:50236950-50242950	6000	1.68333333
chr16:54228399-54231399	3000	0.6
chr16:56744203-56747203	3000	1.06666667

chr16:60971203-60975203	4000	1.65
chr16:61722203-61725203	3000	1.46666667
chr16:61982203-61986203	4000	1.625
chr16:62445203-62448203	3000	0.83333333
chr16:66903604-66908604	5000	0.48
chr16:70257732-70260732	3000	2.43333333
chr16:70262732-70265732	3000	2.06666667
chr16:71882732-71886732	4000	1.075
chr16:72823732-72826732	3000	1.06666667
chr16:75621732-75626732	5000	1.18
chr16:77317732-77320732	3000	0.5
chr16:82398158-82403158	5000	1.34
chr16:89232712-89236712	4000	0.425
chr17:2208950-2212950	4000	2.025
chr17:2439950-2442950	3000	0.9
chr17:4010950-4014950	4000	1.2
chr17:7577950-7582950	5000	1.48
chr17:10613950-10616950	3000	0.63333333
chr17:10894950-10897950	3000	1.13333333
chr17:11834950-11837950	3000	1.13333333
chr17:14004950-14009950	5000	1.94
chr17:15889617-15893617	4000	2.025
chr17:17265777-17271777	6000	1.26666667
chr17:17272777-17278777	6000	1.68333333
chr17:17565777-17568777	3000	0.76666667
chr17:17688777-17695777	7000	1.22857143
chr17:19053777-19058777	5000	1.24
chr17:19437641-19443641	6000	1.18333333
chr17:19445641-19448641	3000	0.43333333
chr17:19829641-19832641	3000	1.13333333
chr17:20592641-20595641	3000	1.83333333
chr17:20713641-20717641	4000	1.85
chr17:22159641-22164641	5000	1.4
chr17:22650861-22653861	3000	1.73333333
chr17:24498861-24501861	3000	0.63333333
chr17:26058861-26061861	3000	1
chr17:31957775-31961775	4000	2
chr17:35753775-35756775	3000	1.76666667
chr17:36268775-36272775	4000	1.275
chr17:36408775-36413775	5000	1.02
chr17:37350176-37353176	3000	1.2
chr17:37515176-37519176	4000	1.625
chr17:37698176-37703176	5000	1.32
chr17:37907176-37910176	3000	1.33333333
chr17:37914176-37917176	3000	1.13333333
chr17:39625176-39629176	4000	0.775
chr17:40744176-40748176	4000	1.95
chr17:43010176-43014176	4000	1.6
chr17:50097176-50100176	3000	0.83333333
chr17:50198176-50201176	3000	1.7
chr17:50245176-50249176	4000	1.05
chr17:50634473-50637473	3000	2.56666667
chr17:53392473-53395473	3000	1.93333333

chr17:53581473-53586473	5000	1.6
chr17:53630473-53633473	3000	1.7
chr17:54745473-54749473	4000	0.975
chr17:54855473-54858473	3000	0.86666667
chr17:58569473-58573473	4000	1.45
chr17:61055079-61058079	3000	0.83333333
chr17:61116079-61119079	3000	0.3
chr17:61314079-61317079	3000	1.3
chr17:61811079-61814079	3000	0.73333333
chr17:62844079-62847079	3000	0.8
chr17:62959079-62962079	3000	0.4
chr17:63023079-63026079	3000	1.23333333
chr17:64604079-64607079	3000	1.53333333
chr17:65983079-65986079	3000	0.76666667
chr17:66199079-66202079	3000	1.43333333
chr17:68801896-68804896	3000	1.63333333
chr17:74897616-74901616	4000	1.225
chr17:75880616-75883616	3000	1.2
chr17:77444616-77449616	5000	1.22
chr17:83051616-83054616	3000	1.03333333
chr17:83070616-83073616	3000	2.3
chr17:84504616-84507616	3000	1.73333333
chr17:88189279-88192279	3000	0.43333333
chr17:88392279-88395279	3000	0.56666667
chr17:88689279-88692279	3000	0.6
chr17:95684279-95688279	4000	0.45
chr17:97016429-97019429	3000	0.8
chr18:897001-901001	4000	1.225
chr18:5709485-5712485	3000	0.5
chr18:8342485-8346485	4000	0.45
chr18:8913485-8916485	3000	0.73333333
chr18:9023485-9026485	3000	0.63333333
chr18:9645485-9648485	3000	1.6
chr18:11376485-11380485	4000	0.55
chr18:11998485-12001485	3000	0.36666667
chr18:12372485-12375485	3000	0.9
chr18:12718485-12721485	3000	0.76666667
chr18:12976485-12981485	5000	1.44
chr18:14437841-14441841	4000	1.7
chr18:16921841-16924841	3000	0.4
chr18:21035841-21039841	4000	0.25
chr18:21096841-21100841	4000	0.925
chr18:25337841-25340841	3000	0.66666667
chr18:27480841-27484841	4000	1.05
chr18:27650841-27654841	4000	1
chr18:29378135-29382135	4000	1.5
chr18:29532037-29535037	3000	1.13333333
chr18:29538037-29541037	3000	0.93333333
chr18:30036571-30040571	4000	1.425
chr18:32003986-32009986	6000	1.21666667
chr18:35808986-35813986	5000	1.52
chr18:38979986-38982986	3000	1.6
chr18:40053961-40056961	3000	1.6

chr18:42532961-42536961	4000	1.725
chr18:47826451-47829451	3000	1.8
chr18:48404451-48410451	6000	1.11666667
chr18:49680451-49683451	3000	0.43333333
chr18:49705451-49708451	3000	0.53333333
chr18:51710451-51713451	3000	0.2
chr18:55547451-55550451	3000	1.2
chr18:58101159-58104159	3000	0.53333333
chr18:58486159-58489159	3000	1.53333333
chr18:58805159-58811159	6000	1.3
chr18:62051418-62056418	5000	1.3
chr18:63123418-63128418	5000	1.84
chr18:63235418-63238418	3000	0.4
chr18:67948418-67951418	3000	0.6
chr18:71014418-71019418	5000	0.8
chr18:71181418-71184418	3000	1
chr18:76324418-76327418	3000	1.76666667
chr18:77912418-77915418	3000	0.36666667
chr18:82792418-82796418	4000	1.25
chr18:84519418-84522418	3000	0.73333333
chr18:84686418-84689418	3000	0.4
chr19:621004-626004	5000	0.36
chr19:9589861-9597861	8000	1.925
chr19:11004861-11007861	3000	1.43333333
chr19:11991585-11995585	4000	0.5
chr19:13664585-13669585	5000	1.64
chr19:14115939-14118939	3000	2.26666667
chr19:15902414-15908414	6000	0.8
chr19:17640414-17643414	3000	0.43333333
chr19:19601414-19607414	6000	1.01666667
chr19:22773563-22778563	5000	1.22
chr19:22793563-22796563	3000	2.33333333
chr19:23531553-23535553	4000	2.025
chr19:24205553-24210553	5000	1.78
chr19:24218553-24222553	4000	1.075
chr19:24378553-24381553	3000	1.36666667
chr19:24512553-24517553	5000	1.68
chr19:24535553-24539553	4000	1.125
chr19:25997352-26000352	3000	0.86666667
chr19:26270352-26276352	6000	0.66666667
chr19:26288352-26291352	3000	1.83333333
chr19:26337352-26341352	4000	0.475
chr19:27691352-27694352	3000	1.23333333
chr19:29535352-29538352	3000	0.8
chr19:29912352-29917352	5000	1.3
chr19:31552352-31555352	3000	1
chr19:41998670-42001670	3000	1.9
chr19:42417670-42420670	3000	1.2
chr19:55027507-55030507	3000	1.1
chr20:225001-231001	6000	0.63333333
chr20:254001-258001	4000	0.625
chr20:328001-333001	5000	0.84
chr20:1247929-1250929	3000	0.3

chr20:1440929-1444929	4000	0.65
chr20:2171508-2176508	5000	0.72
chr20:2686508-2691508	5000	0.82
chr20:2913508-2916508	3000	1.56666667
chr20:3454093-3457093	3000	1.5
chr20:3474093-3479093	5000	1.38
chr20:3531093-3536093	5000	1.46
chr20:4353723-4356723	3000	1.5
chr20:4385723-4389723	4000	1.7
chr20:5035815-5041815	6000	1.36666667
chr20:14434378-14438378	4000	2.025
chr20:25367900-25370900	3000	0.8
chr20:25396900-25400900	4000	1.325
chr20:26832900-26835900	3000	0.6
chr20:29984981-29987981	3000	2
chr20:30056981-30060981	4000	1.875
chr20:30311981-30315981	4000	1.125
chr20:47151981-47155981	4000	1.275
chr20:49229981-49232981	3000	0.86666667
chr20:50680981-50684981	4000	2.1
chr20:52126981-52130981	4000	0.675
chr20:52165981-52169981	4000	0.5
chr20:52523981-52526981	3000	0.3
chr20:52581981-52584981	3000	0.56666667
chr20:52995981-52999981	4000	0.35
chr20:53956981-53960981	4000	1.35
chrX:68206209-68222209	16000	0.70625
chrX:23301999-23317999	16000	0.95625
chrX:25497999-25506999	9000	0.55555556
chrX:157047510-157057510	10000	0.74
chrX:51616361-51621361	5000	0.88
chrX:29670077-29677077	7000	0.95714286
chrX:147979510-147982510	3000	0.43333333
chrX:29679077-29686077	7000	1.02857143
chrX:20121999-20127999	6000	0.13333333
chrX:154828510-154831510	3000	0.4
chrX:51420361-51428361	8000	0.7125
chrX:91355430-91369430	14000	0.64285714
chrX:125504510-125508510	4000	0.325
chrX:69030209-69043209	13000	0.30769231
chrX:27533077-27550077	17000	0.68235294
chrX:152444510-152451510	7000	0.41428571
chrX:16440999-16450999	10000	0.61
chrX:157029510-157041510	12000	0.91666667
chrX:18342999-18347999	5000	0.4
chrX:27346077-27363077	17000	0.81176471
chrX:23439999-23452999	13000	0.8
chrX:94059430-94065430	6000	0.78333333
chrX:20107999-20118999	11000	0.56363636
chrX:101142430-101145430	3000	0.56666667
chrX:26133999-26136999	3000	0.53333333
chrX:149858510-149863510	5000	0.9
chrX:23129999-23135999	6000	0.75

chrX:23501999-23510999	9000	0.72222222
chrX:27443077-27455077	12000	0.525
chrX:17794999-17807999	13000	0.52307692
chrX:58677361-58684361	7000	0.41428571
chrX:106716430-106719430	3000	0.56666667
chrX:68223209-68237209	14000	0.37142857
chrX:97498430-97504430	6000	0.4
chrX:114385430-114389430	4000	3.35
chrX:18146999-18159999	13000	0.52307692
chrX:27896077-27909077	13000	0.66153846
chrX:134417510-134424510	7000	0.75714286
chrX:23426999-23438999	12000	0.9
chrX:7015001-7018001	3000	0.23333333
chrX:145959510-145974510	15000	0.54
chrX:23727999-23733999	6000	0.71666667
chrX:106686430-106690430	4000	0.4
chrX:22052999-22066999	14000	0.81428571
chrX:28830077-28833077	3000	0.56666667
chrX:106700430-106715430	15000	0.34
chrX:23121999-23126999	5000	0.88
chrX:22394999-22398999	4000	0.6
chrX:7525001-7535001	10000	0.77
chrX:51697361-51701361	4000	0.525
