# Supplementary material for: Genome-Wide Locations of Potential Epimutations Associated with Environmentally Induced Epigenetic Transgenerational Inheritance of Disease Using a Sequential Machine Learning Prediction Approach
Source: PLoS One. 2015 Nov 16;10(11):e0142274. doi: 10.1371/journal.pone.0142274 (PMC4646459; doi:10.1371/journal.pone.0142274)
Supplement: S3 Table — (A) Clusters from germ cell predicted DMRs (from 3+ consecutive sites only) (80 sites). (B) Clusters from somatic cell Sertoli-Granulosa predicted DMRs (from 3+ consecutive sites only) (44 sites). (PDF) [file pone.0142274.s003.pdf]

Supplemental Table S3

Clusters from combined datasets and stats (cluster size, number of sites in each cluster)

(A) Clusters from predicted germ cell DMRs (from 3+ consecutive sites only) (80)

	Chromosome	cSTART	cSTOP	Length
1	chr1	32350000	35200000	2850000
2	chr1	55100000	57850000	2750000
3	chr1	109900000	115750000	5850000
4	chr1	216550000	220500000	3950000
5	chr1	222350000	224900000	2550000
6	chr10	49100000	53400000	4300000
7	chr11	1850000	3900000	2050000
8	chr11	27550000	30950000	3400000
9	chr11	72550000	76500000	3950000
10	chr11	78600000	80750000	2150000
11	chr12	19300000	25350000	6050000
12	chr12	30900000	34950000	4050000
13	chr13	19600000	22900000	3300000
14	chr13	24600000	26600000	2000000
15	chr13	72500000	75800000	3300000
16	chr13	108200000	110450000	2250000
17	chr14	25300000	29250000	3950000
18	chr14	3.80E+007	40200000	2200000
19	chr14	54100000	56400000	2300000
20	chr14	60150000	63350000	3200000
21	chr14	65650000	67700000	2050000
22	chr14	71800000	78400000	6600000
23	chr14	87300000	89750000	2450000
24	chr14	92050000	95350000	3300000
25	chr15	28900000	30900000	2000000
26	chr15	50800000	54550000	3750000
27	chr15	61800000	65750000	3950000
28	chr15	73450000	75650000	2200000
29	chr16	41200000	43200000	2000000
30	chr16	77850000	87400000	9550000
31	chr17	50000	6050000	6000000
32	chr17	11200000	18850000	7650000
33	chr17	27100000	41300000	14200000
34	chr17	6.20E+007	6.40E+007	2000000
35	chr18	54350000	56850000	2500000
36	chr18	79850000	83100000	3250000
37	chr19	11250000	1.40E+007	2750000
38	chr19	17650000	19850000	2200000
39	chr19	32750000	34850000	2100000
40	chr2	49350000	51700000	2350000
41	chr2	72050000	74200000	2150000
42	chr2	76400000	79800000	3400000
43	chr2	82100000	85800000	3700000

44	chr2	104200000	110300000	6100000
45	chr2	148450000	152400000	3950000
46	chr2	1.56E+008	158200000	2200000
47	chr2	173650000	176100000	2450000
48	chr2	205300000	207400000	2100000
49	chr20	28250000	30250000	2000000
50	chr3	33850000	36900000	3050000
51	chr3	64150000	67400000	3250000
52	chr3	1.23E+008	128700000	5700000
53	chr4	114900000	118550000	3650000
54	chr4	171450000	174550000	3100000
55	chr5	17800000	2.10E+007	3200000
56	chr5	31800000	34150000	2350000
57	chr5	39500000	41800000	2300000
58	chr5	108350000	111800000	3450000
59	chr5	168750000	172100000	3350000
60	chr6	32400000	39150000	6750000
61	chr6	44100000	47700000	3600000
62	chr6	49900000	52750000	2850000
63	chr6	85250000	88250000	3000000
64	chr7	101650000	105600000	3950000
65	chr7	1.07E+008	110700000	3700000
66	chr7	124550000	1.27E+008	2450000
67	chr7	1.31E+008	134200000	3200000
68	chr8	3300000	10150000	6850000
69	chr8	11600000	14850000	3250000
70	chr8	24200000	28050000	3850000
71	chr8	79900000	83150000	3250000
72	chr8	97400000	1.01E+008	3600000
73	chr9	21700000	26500000	4800000
74	chr9	29550000	33500000	3950000
75	chr9	40650000	42750000	2100000
76	chr9	43950000	46050000	2100000
77	chr9	79300000	81350000	2050000
78	chr9	9.80E+007	101500000	3500000
79	chr9	106650000	109350000	2700000
80	chrX	21250000	2.50E+007	3750000

(B) Clusters from Sertoli-Granulosa predicted DMRs (from 3+ consecutive sites only) (44)

	Chromosome	cSTART	cSTOP	Length
1	chr1	21450000	23450000	2000000
2	chr1	6.90E+007	71300000	2300000
3	chr1	72400000	74700000	2300000
4	chr1	82850000	86900000	4050000
5	chr10	10250000	13850000	3600000
6	chr11	21950000	24650000	2700000
7	chr11	36950000	39350000	2400000
8	chr11	64800000	67950000	3150000
9	chr11	79050000	84300000	5250000
10	chr12	17050000	22550000	5500000
11	chr13	7.00E+005	13250000	12550000
12	chr13	15650000	29500000	13850000
13	chr14	3950000	7700000	3750000
14	chr14	20300000	24050000	3750000
15	chr14	46650000	51150000	4500000
16	chr14	97650000	102500000	4850000
17	chr15	5500000	7550000	2050000
18	chr15	4.60E+007	49650000	3650000
19	chr16	7.00E+006	9.00E+006	2000000
20	chr17	15700000	21400000	5700000
21	chr17	35550000	39350000	3800000
22	chr17	52900000	55350000	2450000
23	chr17	60850000	63300000	2450000
24	chr18	1.10E+007	13350000	2350000
25	chr19	22250000	27950000	5700000
26	chr2	6900000	8900000	2000000
27	chr2	22150000	24350000	2200000
28	chr2	84600000	88300000	3700000
29	chr2	189100000	191700000	2600000
30	chr20	50000	5450000	5400000
31	chr20	50600000	54150000	3550000
32	chr4	184600000	187550000	2950000
33	chr5	8.00E+005	2900000	2100000
34	chr5	4850000	7350000	2500000
35	chr5	76600000	80900000	4300000
36	chr6	8950000	12650000	3700000
37	chr6	17200000	20100000	2900000
38	chr6	101650000	106150000	4500000
39	chr7	2400000	1.00E+007	7600000
40	chr7	11250000	19850000	8600000
41	chr8	17350000	20600000	3250000
42	chr8	34100000	38900000	4800000
43	chr8	81300000	83600000	2300000
